# Supplementary material for: A platform for blue-luminescent carbon-centered radicals
Source: Nat Commun. 2022 Sep 13;13:5367. doi: 10.1038/s41467-022-33130-1 (PMC9470563; doi:10.1038/s41467-022-33130-1)
Supplement: Supplementary file 1 — Supplementary Information [file 41467_2022_33130_MOESM1_ESM.pdf]

Supplementary Information

A Platform for Blue-Luminescent Carbon-Centered Radicals

Xin Li[1], Yi-Lin Wang[1], Chan Chen[1], Yan-Yan Ren[1] & Ying-Feng Han[1]*

[1]Key Laboratory of Synthetic and Natural Functional Molecule of the Ministry of Education, College of Chemistry and Materials Science, Northwest University, Xi'an 710127, P. R. China

*Corresponding author: Prof. Y.-F. Han, E-mail: yfhan@nwu.edu.cn

Table of Contents

1. General information **S2**

2. Synthesis and characterization of 1a-c and 2a-c **S4**

3. Synthesis and characterization of 3a-c and 4a-c **S30**

4. Details of single crystal X-ray diffraction studies **S66**

5. Cartesian coordinates of the optimized geometries by DFT calculations **S76**

6. Abstracted results of TD-DFT calculations **S106**

7. References **S115**

1. General information

Experiments were performed under an inert gas (Ar or N_2) atmosphere using standard Schlenk techniques and glovebox. THF, dioxane and hexane were dried by standard methods and distilled under nitrogen. ^1H, $^{13}C\{^1H\}$ were recorded on Bruker AVANCE III 400. Chemical shifts (*δ*) are expressed in ppm downfield from tetramethylsilane using the residual protonated solvent as an internal standard. Mass spectra were obtained with a Bruker microTOF-Q II mass spectrometer (Bruker Daltonics Corp., USA) in the electrospray ionization (ESI) mode. IR spectra were recorded in Nujol oil using KBr plates as the infrared transmission window on a Nicolet AVATAR 360 FT-IR spectrometer. Elemental analyses were carried out on an Elementar VarioEL III instrument. UV−visible spectra were recorded using an Agilent Technologies Evolution 300 spectrophotometer. 4-Iodo-N,N-diphenylaniline[1], free carbenes **a**−**c**[2,3] were prepared according to the reported methods.

Cyclic voltammetry (CV) experiments were carried out using a CHI660E electrochemical workstation (CH Instruments, Inc). All experiments were carried out under an atmosphere of nitrogen in degassed and anhydrous acetonitrile solution containing Bu_4NPF_6 (0.1 M) at a scan rate of 100 mV s^{-1}. The setup consisted of a glassy carbon working electrode, a platinum wire counter electrode, and a silver wire inserted in a small glass tube fitted with a porous Vycor frit and filled with a $AgNO_3$ solution in acetonitrile (0.01 M). Ferrocene was used as a standard, and all reduction potentials are reported with respect to the $E_{1/2}$ of the Fc^+/Fc redox couple. The continuous wave (CW) EPR spectra were obtained using an X-band Bruker E500 spectrometer at room temperature. The microwave frequency was 9.8 GHz and the modulation amplitude was 0.1 mT. Simulations of the EPR spectra have been performed with Easyspin v5.2.11[4].

The fluorescence experiments were performed on a Horiba Fluorolog-3 spectrometer. Fluorescence decay profiles were recorded on a FLS920 instrument. The experimental quantum yields were determined by recording the emission signals within an integrating light sphere on a FLS980 Photoluminescence Spectrometer (Edinburgh Instruments) equipped with an ozone-free Xenon Arc Lamp (450 W), photomultiplier R928P and double grating excitation and emission monochromators (Czerny-Turner type).

Diffraction data of compounds were collected with a Bruker APEX-II CCD diffractometer. Using Olex2, the structure was solved with the olex2.solve structure solution program using Charge

Flipping and refined with the ShelXL refinement package using Least Squares minimisation[5-7]. All non-hydrogen atoms were refined anisotropically.

DFT calculations were executed using the Gaussian 09 program package[8]. The geometries of the compounds were optimized without symmetry constraints using the crystal structure coordinate as the starting structure. Calculations were performed using the unrestricted Becke three-parameter hybrid functional with Lee–Yang–Parr correlation functional (B3LYP)[9] with the 6-31G(d) basis set. Frequency calculations were carried out to ensure that the optimized geometries were minima on the potential energy surface, in which no imaginary frequencies were observed in any of the compounds. The explicitly spin-adapted TDDFT (X-TDDFT) is performed using the Beijing Density Functional (BDF) package[10].

2. Synthesis and characterization of 1a-c and 2a-c

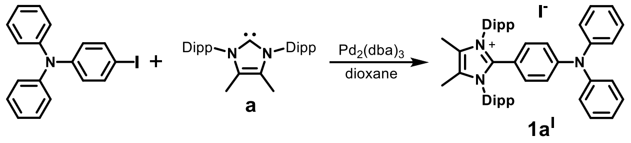

1a^{I}: $Pd_2(dba)_3$ (18.3 mg, 0.02 mmol) was added to a stirred solution of free carbene **a** (416.5 mg, 1.0 mmol) and 4-Iodo-N,N-diphenylaniline (371.0 mg, 1.0 mmol) in dioxane (30 mL). The resulting brown solution was refluxed for overnight. The reaction mixture was cooled to ambient temperature. Then, the solution was concentrated, and hexane was added to precipitate out the solid. The resulting solid was washed with diethyl ether, and dried under vacuum. Yield: 747.6 mg, 95%. m.p. 335 °C. Elemental analysis (%) for $C_{47}H_{54}N_3I$: calcd: C 71.65, H 6.91, N 5.33; found: C 70.78, H 6.54, N 5.28. ^{1}H NMR (400 MHz, CD_3CN): 7.64 (t, J = 7.8 Hz, 2H), 7.45 (d, J = 7.8 Hz, 4H), 7.30 (t, J = 7.6 Hz, 4H), 7.16 (t, J = 6.8 Hz, 2H), 6.95 (d, J = 8.4 Hz, 4H), 6.69 (d, J = 9.1 Hz, 2H), 6.52 (d, J = 9.1 Hz, 2H), 2.49 (sept, J = 6.8 Hz, 4H, C*H*(CH_3)), 2.17 (s, 6H, NCC*H*$_3$), 1.22 (d, J = 6.8 Hz, 12H, CH(C*H*$_3$)), 0.98 (d, J = 6.8 Hz, 12H, CH(C*H*$_3$)) ppm. $^{13}C\{^{1}H\}$ NMR (100 MHz, CD_3CN): 151.8, 146.4, 146.3, 144.1, 133.1, 131.0, 130.8, 130.3, 129.9, 127.2, 126.8, 126.5, 118.8, 112.5, 29.6 (*C*H(CH_3)$_2$), 24.8 (CH(*C*H$_3$)$_2$), 23.5, 10.3 (NC*C*H$_3$) ppm. IR (cm^{-1}): λ = 2975, 2930, 2873, 1645, 1592, 1533, 1490, 1466, 1331, 1301, 1272, 1196, 1061, 808, 759, 700, 521. HRMS (ESI, positive ions): m/z = 660.4285 (calcd for $[C_{47}H_{54}N_3]^+$ 660.4312).

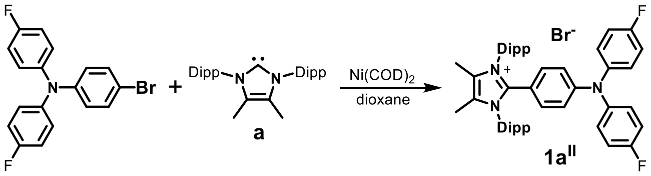

1a^{II}: Free carbene **a** (416.5 mg, 1 mmol), $Ni(COD)_2$ (27.5 mg, 0.1 mmol) and 4-bromo-N,N-bis(4-fluorophen-1-yl)benzeneamine[11] (383 mg, 1 mmol) were dissolved in 25 mL of dry dioxane and stirred at 120 °C for overnight. And a lot of green precipitate was formed. After cooling the reaction mixture to room temperature and this precipitate was filtered, washed with diethyl ether. And dried under vacuum. Yield: 715.1 mg, 92%. m.p. 325 °C. Elemental analysis (%) for $C_{47}H_{52}N_3F_2Br$: calcd: C 72.67, H 6.75, N 5.41; found: C 71.34, H 6.16, N 4.98. ^{1}H NMR (400 MHz, CD_3CN): 7.67 (t, J = 7.8 Hz, 2H), 7.48 (d, J = 7.8 Hz, 4H), 7.15–6.98 (m, 8H), 6.73 (d, J = 9.1 Hz, 2H), 6.49 (d, J = 9.1 Hz, 2H), 2.51 (sept, J = 6.8 Hz, 4H, C*H*(CH_3)), 2.28(s, 6H, C*H*$_3$), 1.26 (d, J = 6.8 Hz, 12H,

CH(CH_3)), 1.01 (d, J = 6.8 Hz, 12H, CH(CH_3)) ppm. $^{13}C\{^1H\}$ NMR (100 MHz, CD_3CN): 162.4, 159.9, 151.8, 146.2, 144.1, 142.3, 133.1, 131.0, 130.2, 129.9, 129.6, 129.5, 126.8, 117.7, 117.6, 117.5, 112.2, 29.6 (CH(CH_3)$_2$), 24.8, 23.5 (CH(CH$_3$)$_2$), 10.2 (NCCH$_3$) ppm. IR (cm^{-1}): λ = 2970, 2928, 2871, 1639, 1594, 1531, 1494, 1461, 1334, 1274, 1193, 1065, 810, 760, 703, 522. HRMS (ESI, positive ions): m/z = 696.4052 (calcd for $[C_{47}H_{52}N_3F_2]^+$ 696.4124).

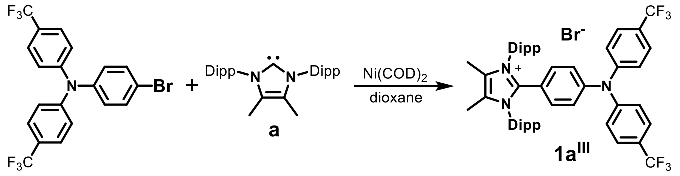

1a^{III}: 4-bromo-N,N-bis(4-trifluoromethyl)benzeneamine[11] (459.01 mg, 1.0 mmol), carbene **a** (416.5 mg, 1 mmol) and Ni(COD)$_2$ (27.5 mg, 0.1 mmol) were used following the previous procedure. Yield: 842.2 mg, 96%. m.p. 332 °C. Elemental analysis (%) for $C_{49}H_{52}N_3F_6Br$: calcd: C 67.12, H 5.98, N 4.79; found: C 66.32, H 5.78, N 4.68. ^1H NMR (400 MHz, CD_3CN): 7.65 (t, J = 7.8 Hz, 2H), 7.57 (d, J = 8.4 Hz, 4H), 7.47 (d, J = 7.8 Hz, 4H), 7.03 (d, J = 8.4 Hz, 4H), 6.86–6.76 (m, 4H), 2.49 (sept, J = 6.8 Hz, 4H, CH(CH$_3$)), 2.23(s, 6H, CH_3), 1.24 (d, J = 6.8 Hz, 12H, CH(CH_3)), 1.00 (d, J = 6.8 Hz, 12H, CH(CH_3)) ppm. $^{13}C\{^1H\}$ NMR (100 MHz, CD_3CN): 150.3, 149.8, 146.3, 143.4, 133.2, 131.4, 130.9, 129.7, 127.9, 127.9, 127.8, 127.8, 126.9, 126.6, 126.5, 126.2, 123.0, 116.0, 29.6 (CH(CH$_3$)$_2$), 24.8, 23.5 (CH(CH$_3$)$_2$), 10.2 (NCCH$_3$) ppm. IR (cm^{-1}): λ = 2979, 2932, 2871, 1643, 1591, 1539, 1489, 1463, 1332, 1306, 1271, 1195, 1063, 810, 761, 703, 520. HRMS (ESI, positive ions): m/z = 796.4012 (calcd for $[C_{49}H_{52}N_3F_6]^+$ 796.4060).

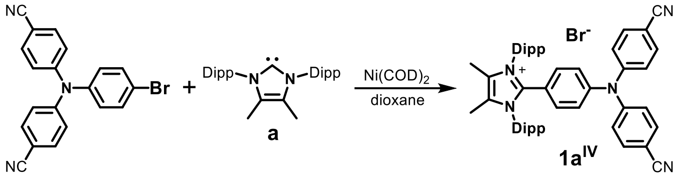

1a^{IV}: 4,4'-[(4-Bromophenyl)imino]bisbenzonitrile[12] (373 mg, 1.0 mmol), carbene **a** (416.5 mg, 1 mmol) and Ni(COD)$_2$ (27.5 mg, 0.1 mmol) were used following the previous procedure. Yield: 750.8 mg, 95%. m.p. 312 °C. Elemental analysis (%) for $C_{49}H_{52}N_5Br$: calcd: C 74.41, H 6.63, N 8.86; found: C 73.14, H 6.23, N 8.73. ^1H NMR (400 MHz, CD_3CN): 7.66 (t, J = 7.8 Hz, 2H), 7.59 (d, J = 8.7 Hz, 4H), 7.47 (d, J = 7.8 Hz, 4H), 6.96 (d, J = 8.7 Hz, 4H), 6.85 (s, 4H), 2.49 (sept, J = 6.8 Hz, 4H, CH(CH$_3$)), 2.19 (s, 2H), 2.15 (s, 4H), 1.24 (d, J = 6.8 Hz, 12H, CH(CH_3)), 1.00 (d, J =

6.8 Hz, 12H, CH(CH_3)) ppm. $^{13}C\{^1H\}$ NMR (100 MHz, CD_3CN): 150.3, 149.8, 146.3, 143.4, 133.2, 131.4, 130.9, 129.7, 127.9, 127.9, 127.9, 127.8, 126.9, 126.6, 126.5, 126.2, 123.0, 116.0, 29.6 (*C*H(CH_3)$_2$), 24.8, 23.5 (CH(*C*H_3)$_2$), 10.2 (NC*C*H_3) ppm. IR (cm^{-1}): λ = 2975, 2932, 2871, 1643, 1590, 1531, 1493, 1464, 1332, 1301, 1275, 1197, 1060, 806, 755, 704, 519. HRMS (ESI, positive ions): *m*/*z* = 710.4171 (calcd for $[C_{49}H_{52}N_5]^+$ 710.4217).

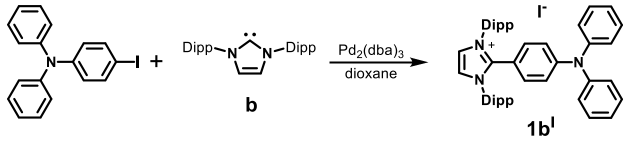

1b^{I}: 4-Iodo-N,N-diphenylaniline (371.0 mg, 1.0 mmol), carbene **b** (388.5 mg, 1.0 mmol) and $Pd_2(dba)_3$ (18.3 mg, 0.02 mmol) were used following the previous procedure. Yield: 577.4 mg, 76%. m.p. 295 °C. Elemental analysis (%) for $C_{45}H_{50}N_3I$: calcd: C 71.13, H 6.63, N 5.53; found: C 70.75, H 6.34, N 5.26. ^1H NMR (400 MHz, CD_3CN): 7.82 (s, 2H, NC*H*), 7.61 (t, *J* = 7.8 Hz, 2H), 7.42 (d, *J* = 7.8 Hz, 4H), 7.32 (t, *J* = 7.8 Hz, 4H), 7.18 (t, *J* = 7.5 Hz, 2H), 6.98 (d, *J* = 7.5 Hz, 4H), 6.69 (d, *J* = 9.0 Hz, 2H), 6.62 (d, *J* = 9.0 Hz, 2H), 2.47 (sept, *J* = 6.8 Hz, 4H, C*H*(CH_3)), 1.23 (d, *J* = 6.8 Hz, 12H, CH(CH_3)), 1.05 (d, *J* = 6.8 Hz, 12H, CH(CH_3)) ppm. $^{13}C\{^1H\}$ NMR (100 MHz, CD_3CN): 152.4, 146.8, 146.3, 145.9, 133.0, 131.3, 130.9, 127.3, 126.7, 126.4, 126.3, 119.0, 111.1, 30.0 (*C*H(CH_3)), 25.3 (CH(*C*H_3)), 22.7 ppm. IR (cm^{-1}): λ = 2969, 2924, 2867, 1589, 1527, 1477, 1443, 1341, 1301, 1268, 1189, 1064, 934, 804, 759, 702, 521, 453. HRMS (ESI, positive ions): *m*/*z* = 632.3977 (calcd for $[C_{45}H_{50}N_3]^+$ 632.3999).

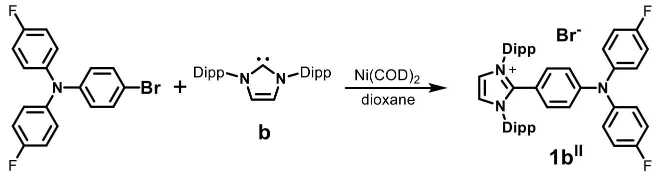

1b^{II}: 4-bromo-N,N-bis(4-fluorophen-1-yl)benzeneamine (383 mg, 1 mmol), carbene **b** (388.5 mg, 1 mmol) and $Ni(COD)_2$ (27.5 mg, 0.1 mmol) were used following the previous procedure. Yield: 696.8 mg, 93%. m.p. 287 °C. Elemental analysis (%) for $C_{45}H_{48}N_3F_2Br$: calcd: C 72.18, H 6.46, N 5.61; found: C 71.23, H 6.28, N 5.14. ^1H NMR (400 MHz, CD_3CN): 7.85 (s, 2H, NC*H*), 7.61 (t, *J* = 7.8 Hz, 2H), 7.42 (d, *J* = 7.8 Hz, 4H), 7.13–7.00 (m, 8H), 6.69 (d, *J* = 9.1 Hz, 2H), 6.54 (d, *J* = 9.1 Hz, 2H), 2.46 (sept, *J* = 6.8 Hz, 4H, C*H*(CH_3)), 1.23 (d, *J* = 6.8 Hz, 12H, CH(CH_3)), 1.04 (d, *J* = 6.8 Hz, 12H, CH(CH_3)) ppm. $^{13}C\{^1H\}$ NMR (100 MHz, CD_3CN): 162.5, 160.1, 152.5, 146.7,

145.9, 142.2, 132.9, 131.3, 129.7, 129.7, 126.4, 126.2, 117.8, 117.8, 117.6, 110.9, 30.0 (*C*H(CH_3)$_2$), 25.3, 22.7 (CH(*C*H_3)$_2$) ppm. IR (cm^{-1}): λ = 2948, 2921, 2875, 1638, 1600, 1534, 1451, 1364, 1332, 1224, 1171, 1060, 840, 812, 755, 728, 655, 451. HRMS (ESI, positive ions): *m*/*z* = 668.3762 (calcd for $[C_{45}H_{48}N_3F_2]^+$ 668.3811).

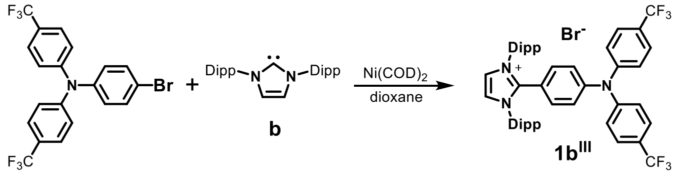

1b^{III}: 4-bromo-N,N-bis(4-trifluoromethyl)benzeneamine (459.01 mg, 1.0 mmol), carbene **b** (388.5 mg, 1 mmol) and Ni(COD)$_2$ (27.5 mg, 0.1 mmol) were used following the previous procedure. Yield: 806.8 mg, 95%. m.p. 305 °C. Elemental analysis (%) for $C_{47}H_{48}N_3F_6Br$: calcd: C 66.51, H 5.70, N 4.95; found: C 65.45, H 5.12, N 4.65. ^{1}H NMR (400 MHz, CD_3CN): 7.97 (s, 2H, NC*H*), 7.65–7.57 (m, 6H), 7.44 (d, *J* = 7.8 Hz, 4H), 7.05 (d, *J* = 8.4 Hz, 4H), 6.89 (d, *J* = 8.9 Hz, 2H), 6.82 (d, *J* = 8.9 Hz, 2H), 2.48 (sept, *J* = 6.8 Hz, 4H, C*H*(CH_3)), 1.25 (d, *J* = 6.8 Hz, 12H, CH(C*H*$_3$)), 1.06 (d, *J* = 6.8 Hz, 12H, CH(C*H*$_3$)) ppm. ^{13}C{^{1}H} NMR (100 MHz, CD_3CN): 150.9, 149.7, 146.1, 145.9, 133.1, 131.7, 131.1, 127.9, 127.0, 126.9, 126.7, 126.4, 126.3, 123.8, 123.2, 30.1 (*C*H(CH_3)$_2$), 25.4, 22.8 (CH(*C*H_3)$_2$) ppm. IR (cm^{-1}): λ = 2960, 2921, 2874, 1637, 1608, 1527, 1445, 1372, 1330, 1226, 1175, 1061, 838, 755, 733, 654, 455. HRMS (ESI, positive ions): *m*/*z* = 768.3675 (calcd for $[C_{47}H_{48}N_3F_6]^+$ 768.3747).

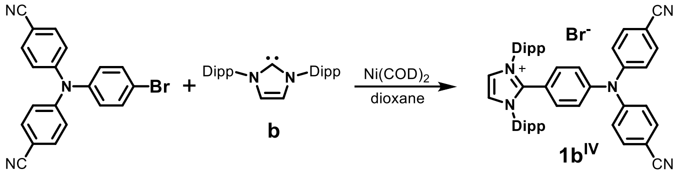

1b^{IV}: 4,4'-[(4-Bromophenyl)imino]bisbenzonitrile (373 mg, 1.0 mmol), carbene **b** (388.5 mg, 1 mmol) and Ni(COD)$_2$ (27.5 mg, 0.1 mmol) were used following the previous procedure. Yield: 732.7 mg, 96%. m.p. 295 °C. Elemental analysis (%) for $C_{47}H_{48}N_5Br$: calcd: C 74.00, H 6.34, N 9.18; found: C 72.28, H 5.87, N 8.35. ^{1}H NMR (400 MHz, CD_3CN): 7.95 (s, 2H, NC*H*), 7.61 (m, 6H), 7.44 (d, *J* = 7.8 Hz, 4H), 7.01–6.89 (m, 6H), 6.85 (d, *J* = 7.8 Hz, 2H), 2.47 (sept, *J* = 6.8 Hz, 4H, C*H*(CH_3)), 1.25 (d, *J* = 6.8 Hz, 12H, CH(C*H*$_3$)), 1.06 (d, *J* = 6.8 Hz, 12H, CH(C*H*$_3$)) ppm. ^{13}C{^{1}H} NMR (100 MHz, CD_3CN): 150.15, 149.96, 145.84, 134.80, 133.09, 131.74, 130.92, 126.87,

126.28, 125.98, 124.67, 119.28, 115.83, 108.33, 29.98 ($CH(CH_3)_2$), 25.36, 22.71 ($CH(CH_3)_2$) ppm. IR (cm^{-1}): λ = 2958, 2922, 2882, 1639, 1535, 1454, 1364, 1336, 1230, 1171, 1063, 836, 804, 753, 730, 654, 452. HRMS (ESI, positive ions): *m*/*z* = 682.3847 (calcd for $[C_{47}H_{48}N_5]^+$ 682.3904).

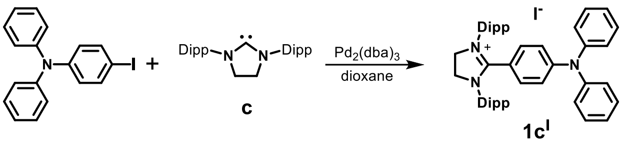

1c^I

1c^I: 4-Iodo-N,N-diphenylaniline (371.0 mg, 1.0 mmol), carbene **c** (390.5 mg, 1.0 mmol) and $Pd_2(dba)_3$ (18.3 mg, 0.02 mmol) were used following the previous procedure. Yield: 646.8 mg, 85%. m.p. 298 °C. Elemental analysis (%) for $C_{45}H_{52}N_3I$: calcd: C 70.95, H 6.88, N 5.52; found: C 68.97, H 6.57, N 5.38. ^1H NMR (400 MHz, CD_3CN): 7.48 (t, *J* = 7.8 Hz, 2H), 7.33 (d, *J* = 7.8 Hz, 4H), 7.30 (t, *J* = 7.7 Hz, 2H), 7.20 (t, *J* = 7.4 Hz, 2H), 6.98 (d, *J* = 7.4 Hz, 4H), 6.76 (d, *J* = 9.2 Hz, 2H), 6.52 (d, *J* = 9.2 Hz, 2H), 4.38 (s, 4H, NCH_2), 3.06 (sept, *J* = 6.8 Hz, 4H, $CH(CH_3)$), 1.33 (d, *J* = 6.8 Hz, 12H, $CH(CH_3)$), 1.02 (d, *J* = 6.8 Hz, 12H, $CH(CH_3)$) ppm. $^{13}C\{^1H\}$ NMR (100 MHz, CD_3CN): 166.9, 153.6, 146.7, 145.9, 132.9, 132.3, 131.9, 130.9, 127.6, 127.1, 126.6, 117.8, 110.9, 54.3, 29.7 ($CH(CH_3)_2$), 25.9 ($CH(CH_3)_2$), 23.4 ppm. IR (cm^{-1}): λ = 2958, 2867, 1589, 1527, 1448, 1330, 1279, 1189, 1046, 934, 804, 759, 691, 526, 442. HRMS (ESI, positive ions): *m*/*z* = 634.4110 (calcd for $[C_{45}H_{52}N_3]^+$ 634.4156).

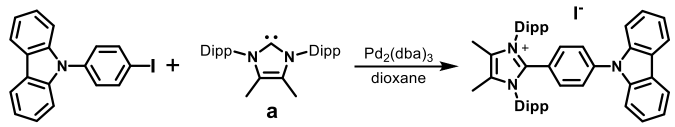

2a

2a: 9-(4-Iodophenyl)carbazole (369.2 mg, 1.0 mmol), carbene **a** (416.5 mg, 1.0 mmol) and $Pd_2(dba)_3$ (18.3 mg, 0.02 mmol) were used following the previous procedure. Yield: 769.2 mg, 97 %. m.p. 345 °C. Elemental analysis (%) for $C_{47}H_{52}N_3I$: calcd: C 71.83, H 6.67, N 5.35; found: C 70.12, H 6.23, N 4.98. ^1H NMR (400 MHz, CD_2Cl_2): 8.10 (d, *J* = 7.4 Hz, 2H), 7.70 (t, *J* = 8.0 Hz, 2H), 7.49 (d, *J* = 8.0 Hz, 4H), 7.45 (d, *J* = 9.1 Hz, 2H), 7.37 (t, *J* = 7.7 Hz, 2H), 7.29 (d, *J* = 7.4 Hz, 2H), 7.16–7.19 (m, 4H), 2.49 (sept, *J* = 6.8 Hz, 4H, $CH(CH_3)$), 2.33 (s, 6H, CH_3), 1.35 (d, *J* = 6.8 Hz, 12H, $CH(CH_3)$), 1.10 (d, *J* = 6.8 Hz, 12H, $CH(CH_3)$) ppm. $^{13}C\{^1H\}$ NMR (100 MHz, CD_2Cl_2): 145.2, 142.7, 141.5, 139.8, 133.0, 130.7, 130.5, 128.5, 126.7, 126.5, 126.4, 124.1, 121.2, 120.6, 119.3, 109.4, 29.3 ($CH(CH_3)$), 24.8 ($CH(CH_3)$), 23.3, 10.5 ppm. IR (cm^{-1}): λ = 2958, 2924, 2879, 1641, 1600, 1538, 1454, 1369, 1335, 1228, 1172, 1063, 838, 809, 753, 730, 651, 453. HRMS (ESI,

positive ions): *m*/*z* = 658.4180 (calcd for $[C_{47}H_{52}N_3]^+$ 658.4156).

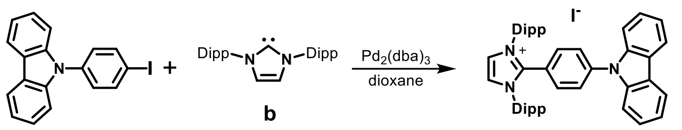

2b

2b: 9-(4-Iodophenyl)carbazole (369.2 mg, 1.0 mmol), carbene **b** (388.5 mg, 1.0 mmol) and $Pd_2(dba)_3$ (18.3 mg, 0.02 mmol) were used following the previous procedure. Yield: 692.8 mg, 91%. m.p. 312 °C. Elemental analysis (%) for $C_{45}H_{48}N_3I$: calcd: C 71.32, H 6.38, N 5.55; found: C 69.58, H 5.98, N 5.18. ^{1}H NMR (400 MHz, CD_3CN): 8.14 (d, *J* = 7.7 Hz, 2H), 8.03 (s, 2H, NC*H*), 7.65 (t, *J* = 8.0 Hz, 2H), 7.62 (d, *J* = 8.9 Hz, 2H), 7.48 (d, *J* = 8.0 Hz, 4H), 7.38 (t, *J* = 7.1 Hz, 2H), 7.29 (t, *J* = 7.1 Hz, 2H), 7.24 (d, *J* = 8.9 Hz, 2H), 7.19 (d, *J* = 8.0 Hz, 2H), 2.57 (sept, *J* = 6.8 Hz, 4H, C*H*(CH_3)), 1.30 (d, *J* = 6.8 Hz, 12H, CH(C*H*$_3$)), 1.15 (d, *J* = 6.8 Hz, 12H, CH(C*H*$_3$)) ppm. $^{13}C\{^1H\}$ NMR (100 MHz, CD_3CN): 146.2, 145.9, 145.8, 142.3, 140.5, 133.4, 132.2, 130.8, 128.1, 127.4, 126.5, 124.7, 121.9, 121.5, 119.8, 111.3, 30.2 (*C*H(CH_3)), 25.7 (CH(*C*H$_3$)), 22.9 ppm. IR (cm^{-1}): λ = 2963, 2924, 2873, 1613, 1533, 1448, 1369, 1335, 1228, 1178, 1053, 843, 809, 748, 725, 629, 453. HRMS (ESI, positive ions): *m*/*z* = 630.3895 (calcd for $[C_{45}H_{48}N_3]^+$ 630.3843).

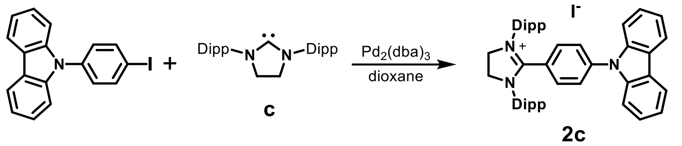

2c

2c: 9-(4-Iodophenyl)carbazole (369.2 mg, 1.0 mmol), carbene **c** (390.5 mg, 1.0 mmol) and $Pd_2(dba)_3$ (18.3 mg, 0.02 mmol) were used following the previous procedure. Yield: 703.2 mg, 92 %. m.p. 327 °C. Elemental analysis (%) for $C_{45}H_{50}N_3I$: calcd: C 71.13, H 6.63, N 5.53; found: C 70.02, H 6.12, N 5.06. ^{1}H NMR (400 MHz, CD_2Cl_2): 8.09 (d, *J* = 7.9 Hz, 2H), 7.47–7.53 (m, 4H), 7.25–7.38 (m, 10H), 7.17 (d, *J* = 7.9 Hz, 2H), 4.84 (s, 4H, NCH_2), 3.14 (sept, *J* = 6.7 Hz, 4H, C*H*(CH_3)), 1.47 (d, *J* = 6.7 Hz, 12H, CH(CH_3)), 1.13 (d, *J* = 6.7 Hz, 12H, CH(C*H*$_3$)) ppm. $^{13}C\{^1H\}$ NMR (100 MHz, CD_2Cl_2): 165.9, 145.8, 143.3, 139.7, 131.8, 131.7, 131.2, 126.9, 126.7, 126.1, 124.3, 121.6, 120.8, 118.9, 109.5, 54.9, 29.5 (*C*H(CH_3)), 26.2 (CH(*C*H$_3$)), 23.4 ppm. IR (cm^{-1}): λ = 2963, 2924, 2867, 1589, 1523, 1488, 1431, 1336, 1302, 1262, 1194, 1063, 826, 759, 702, 492. HRMS (ESI, positive ions): *m*/*z* = 632.4030 (calcd for $[C_{45}H_{50}N_3]^+$ 632.3999).

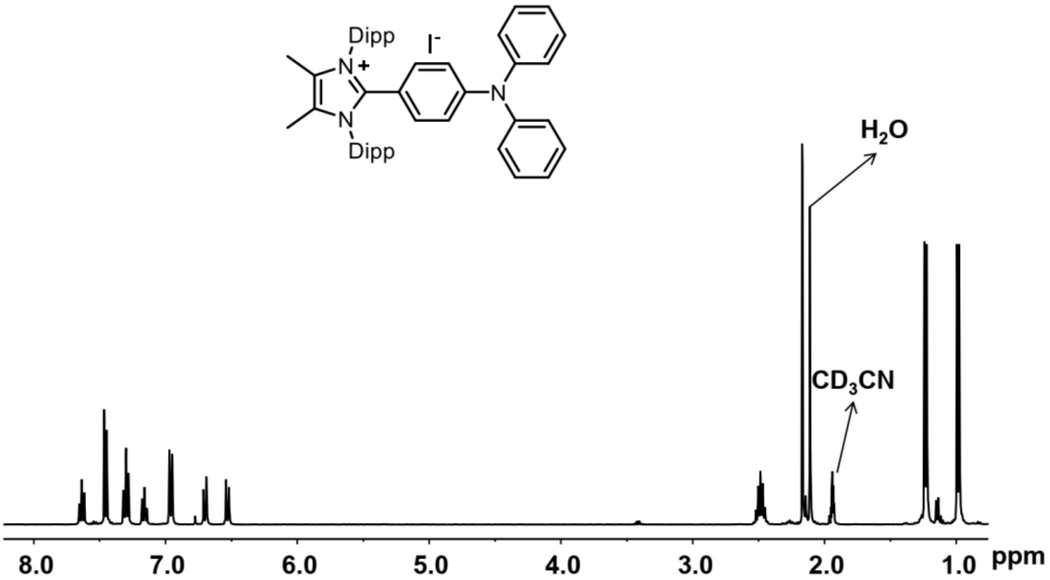

Supplementary Fig. 1. ^{1}H NMR spectrum in CD_3CN of **1a$^{\mathbf{I}}$**.

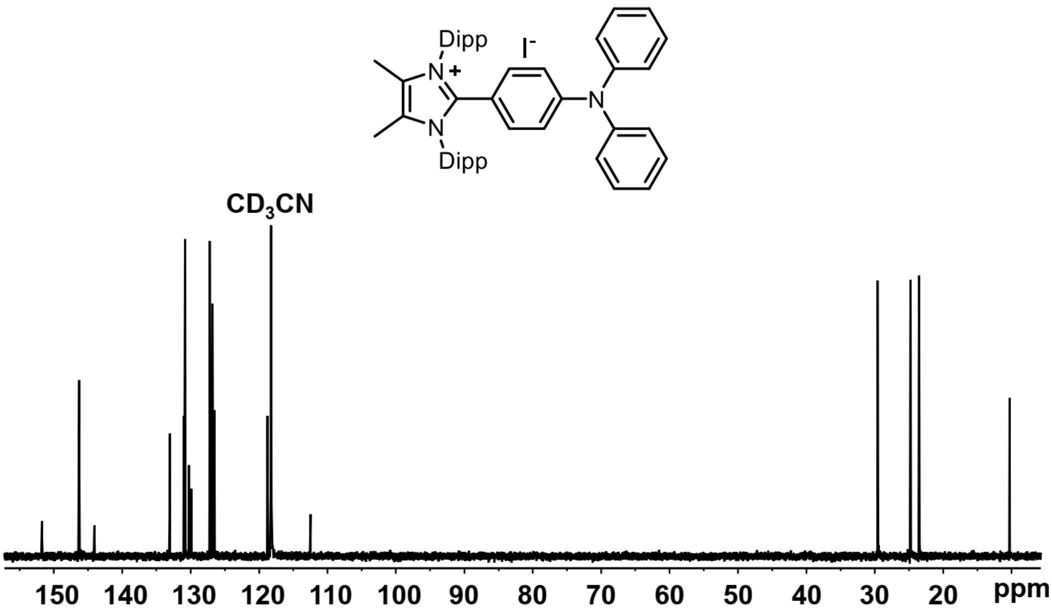

Supplementary Fig. 2. $^{13}C\{^{1}H\}$ NMR spectrum in CD_3CN of **1a$^{\mathbf{I}}$**.

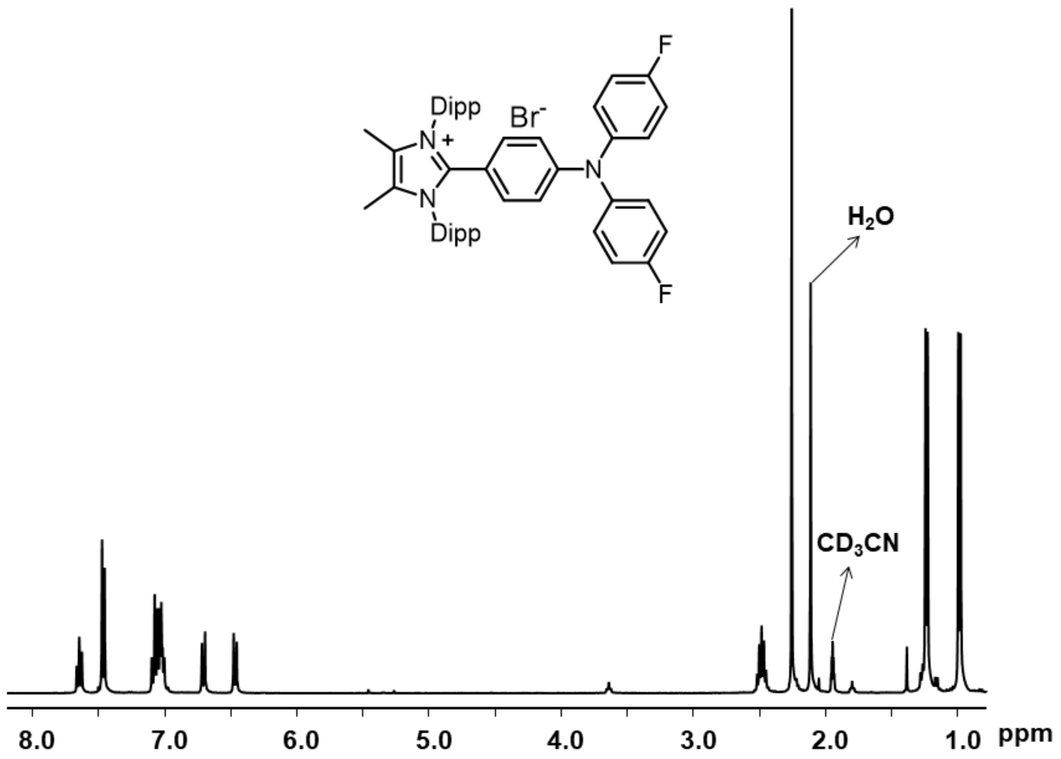

Supplementary Fig. 3. ^{1}H NMR spectrum in CD_3CN of $\mathbf{1a^{II}}$.

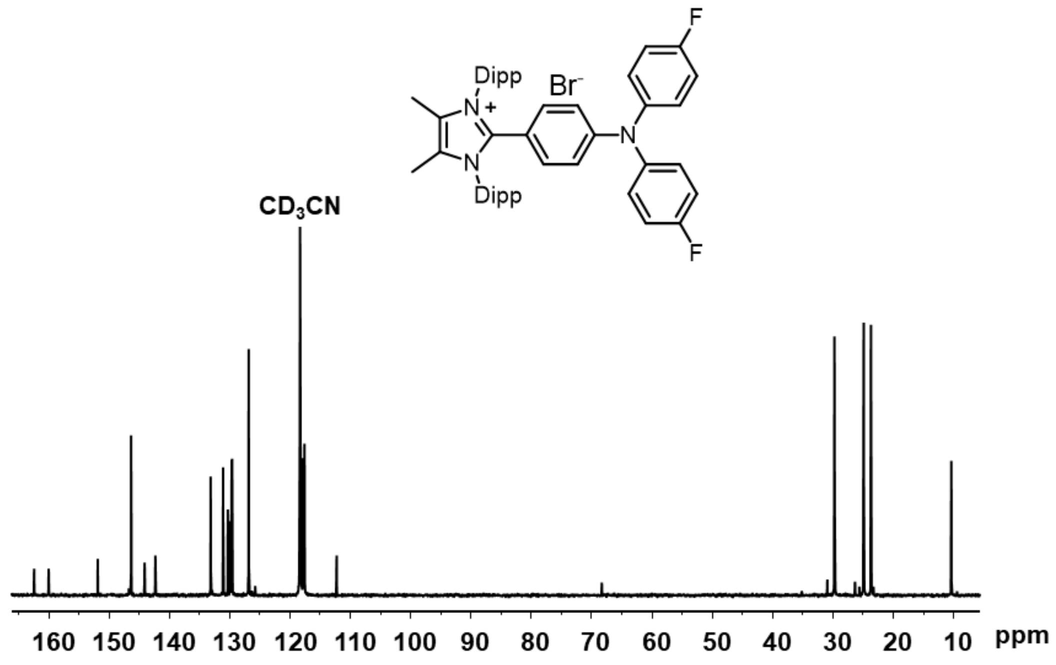

Supplementary Fig. 4. $^{13}C\{^{1}H\}$ NMR spectrum in CD_3CN of $\mathbf{1a^{II}}$.

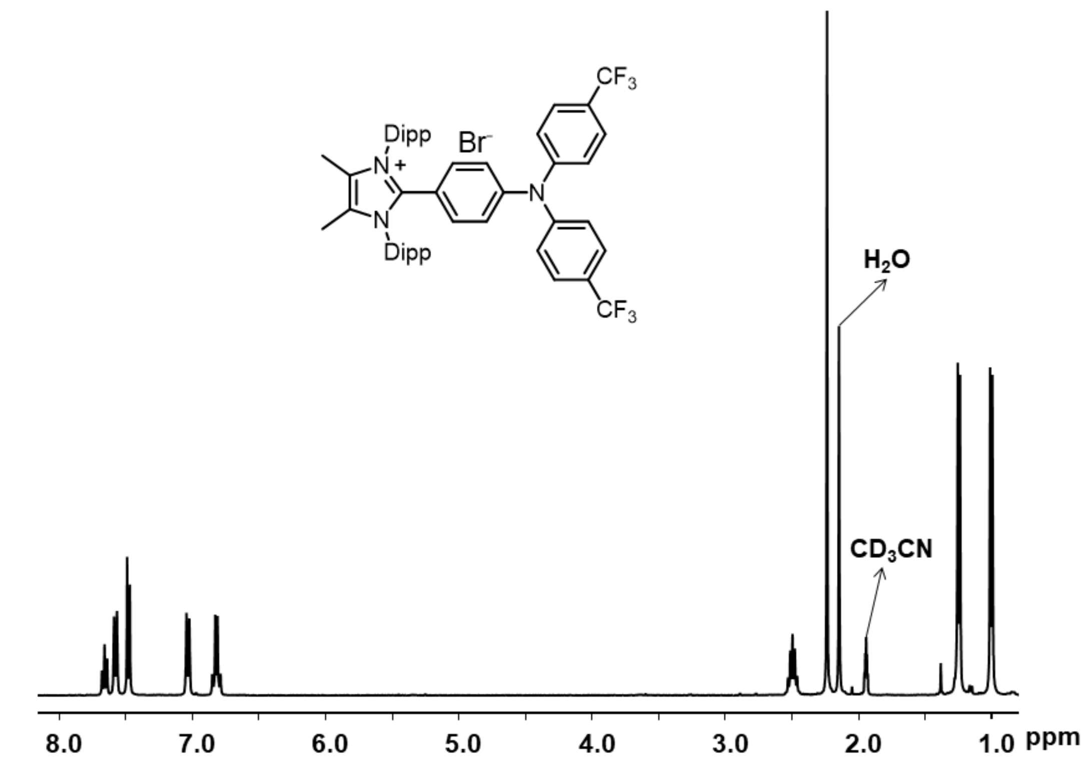

Supplementary Fig. 5. ^{1}H NMR spectrum in CD_3CN of **1a**$^{\mathbf{III}}$.

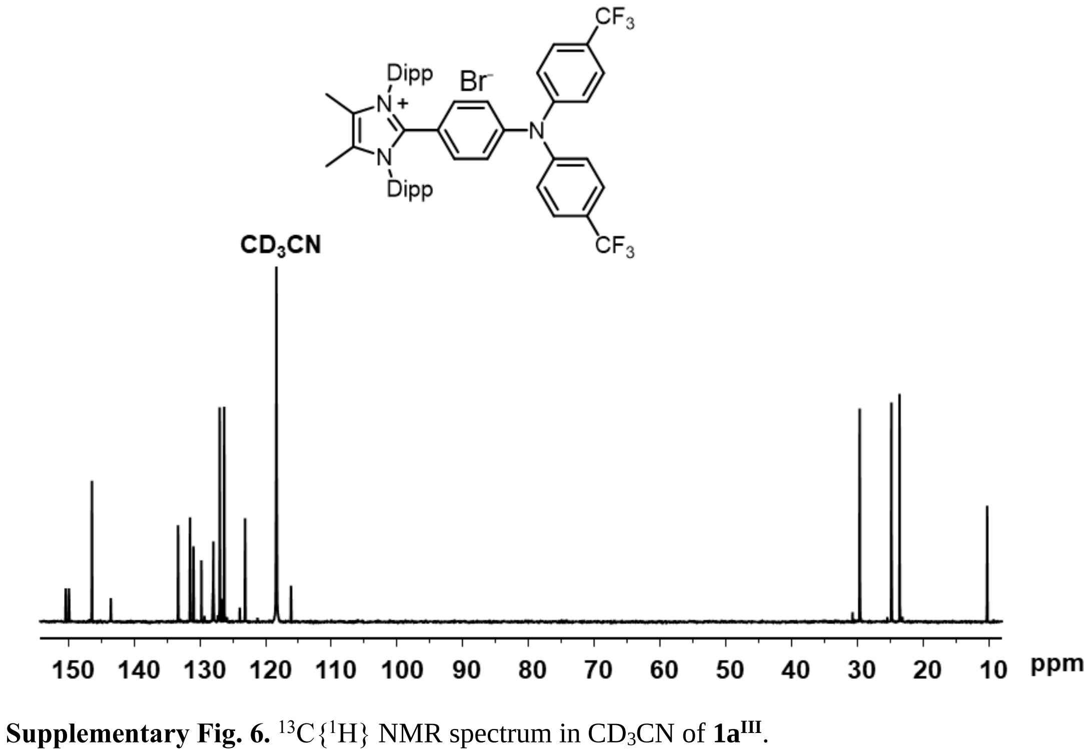

Supplementary Fig. 6. ^{13}C{^{1}H} NMR spectrum in CD_3CN of **1a**$^{\mathbf{III}}$.

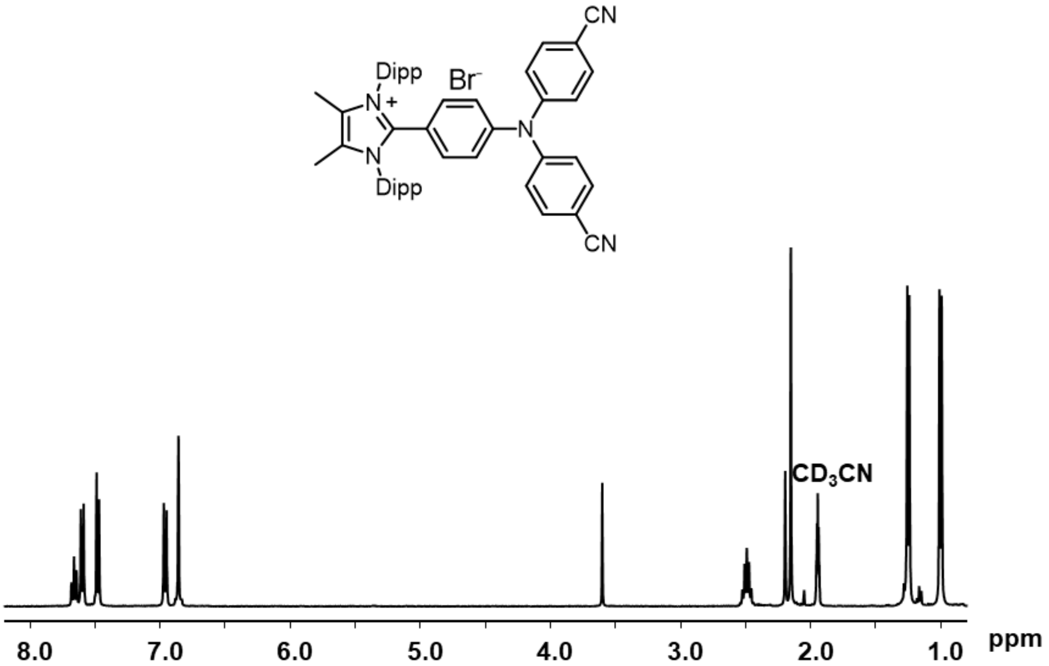

Supplementary Fig. 7. ^{1}H NMR spectrum in CD_3CN of **1a^{IV}**.

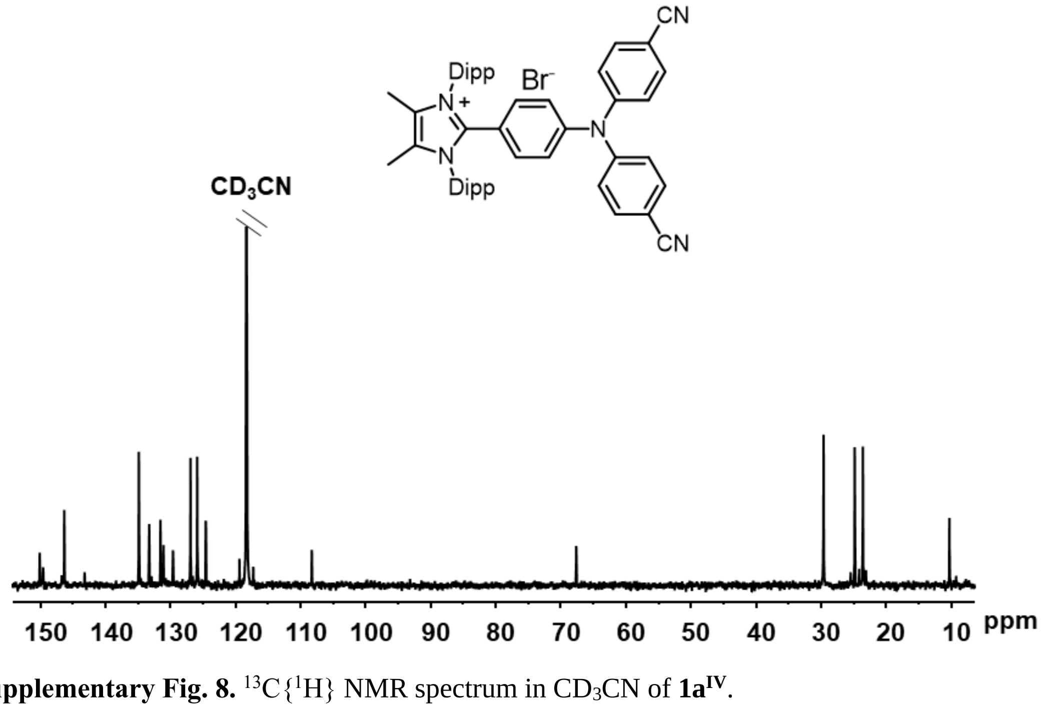

Supplementary Fig. 8. $^{13}C\{^{1}H\}$ NMR spectrum in CD_3CN of **1a^{IV}**.

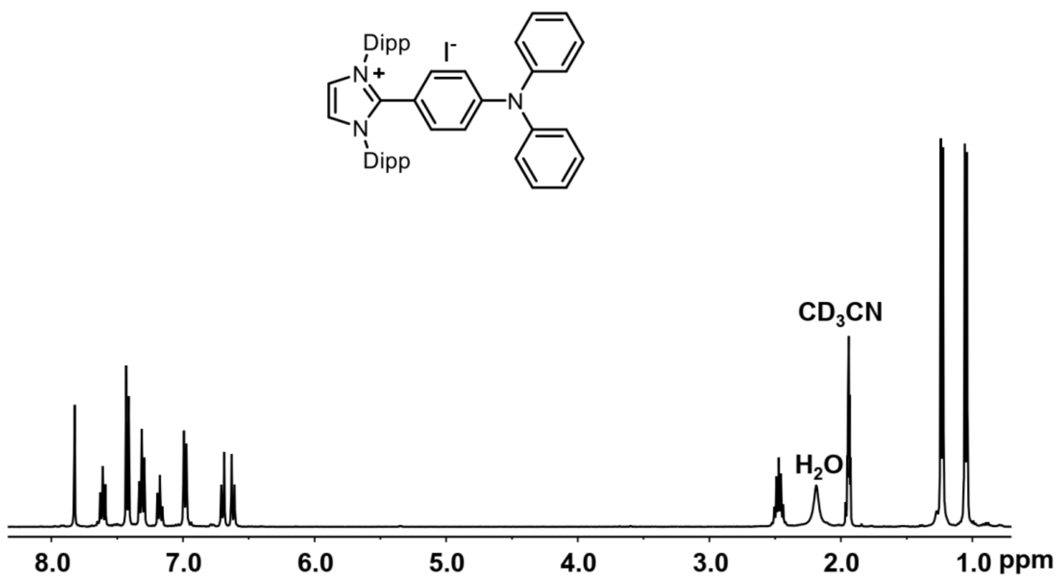

Supplementary Fig. 9. ^{1}H NMR spectrum in CD_3CN of **1b^{I}**.

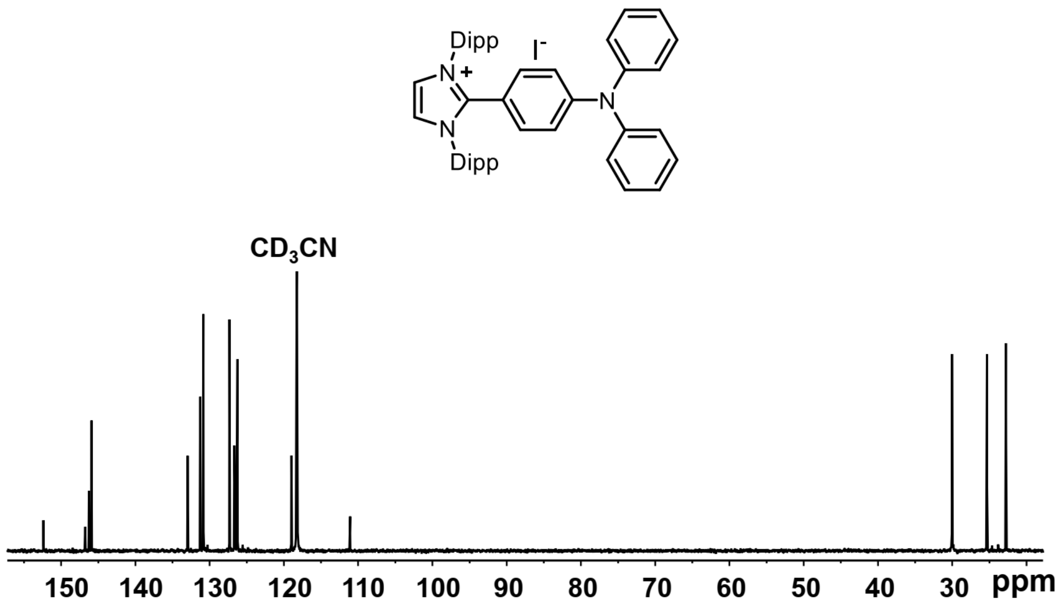

Supplementary Fig. 10. $^{13}C\{^{1}H\}$ NMR spectrum in CD_3CN of **1b^{I}**.

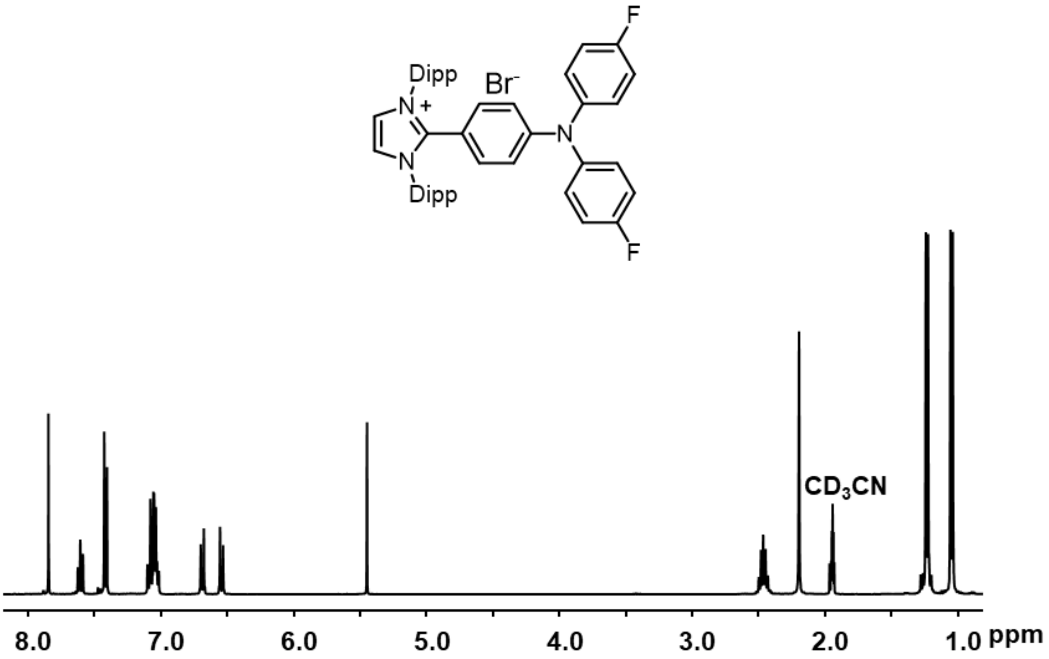

Supplementary Fig. 11. ^{1}H NMR spectrum in CD_3CN of **1b^{II}**.

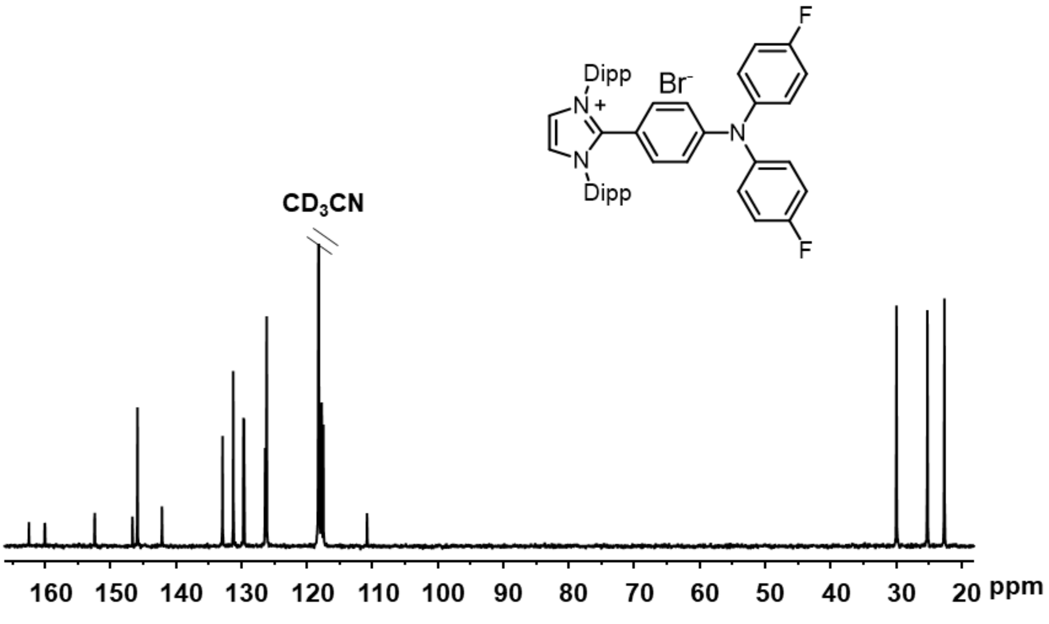

Supplementary Fig. 12. $^{13}C\{^{1}H\}$ NMR spectrum in CD_3CN of **1b^{II}**.

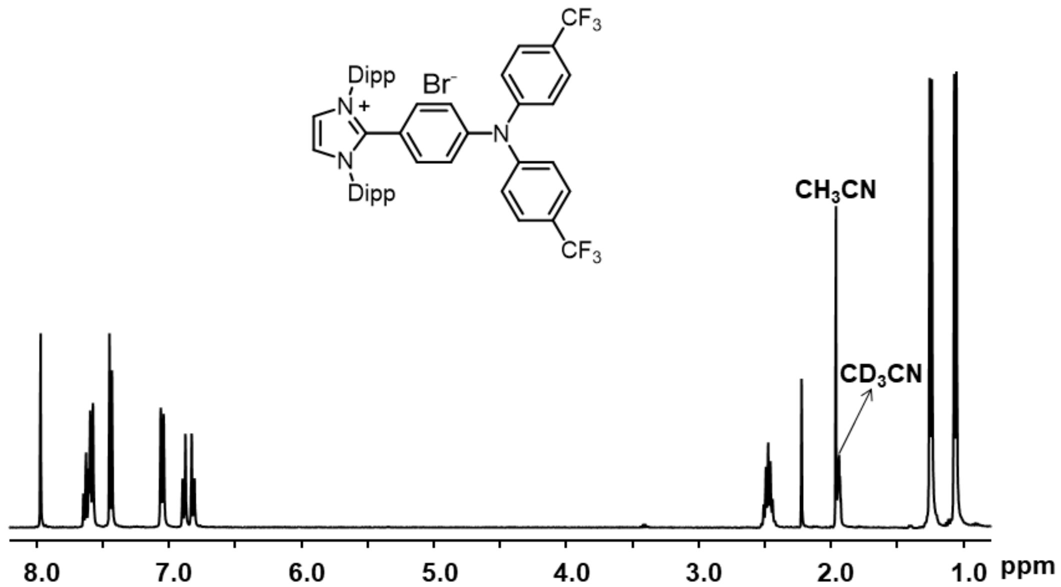

Supplementary Fig. 13. ^{1}H NMR spectrum in CD_3CN of **1b^{III}**.

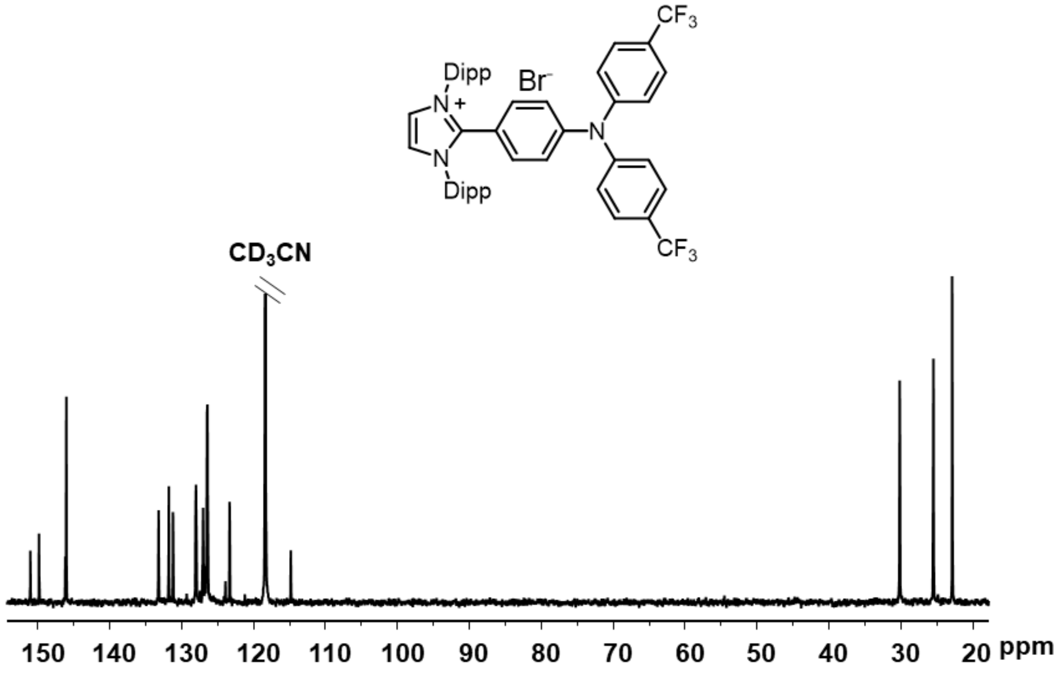

Supplementary Fig. 14. $^{13}C\{^{1}H\}$ NMR spectrum in CD_3CN of **1b^{III}**.

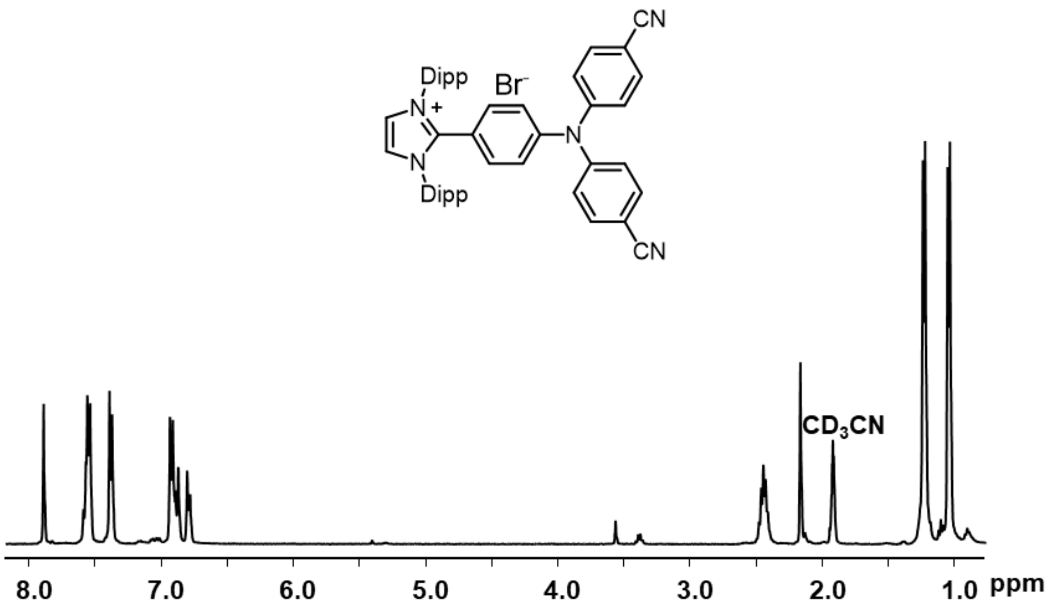

Supplementary Fig. 15. ^{1}H NMR spectrum in CD_3CN of $\mathbf{1b^{IV}}$.

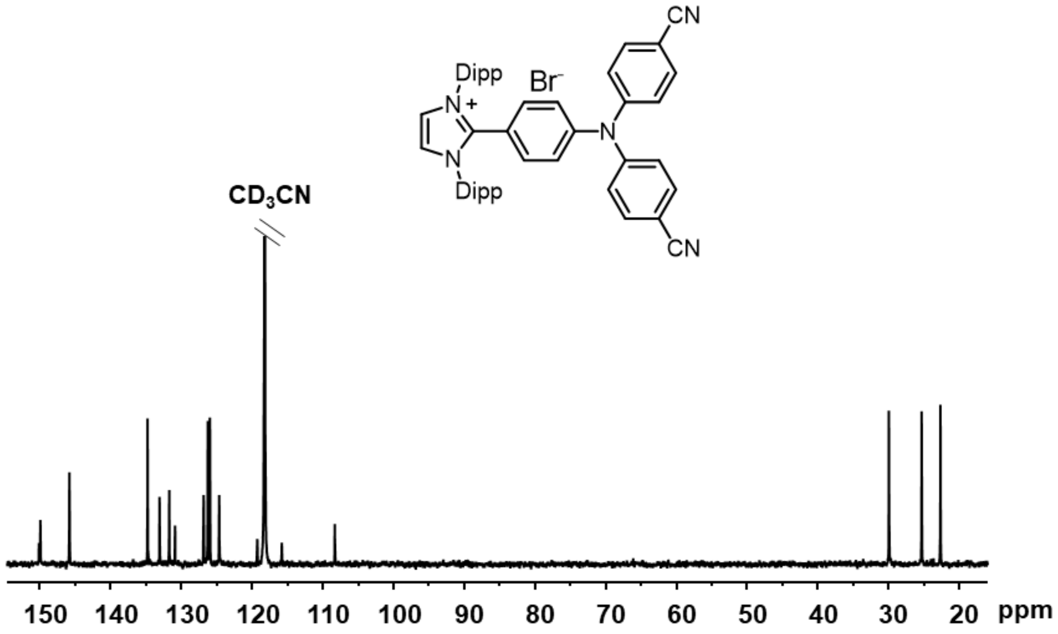

Supplementary Fig. 16. $^{13}C\{^{1}H\}$ NMR spectrum in CD_3CN of $\mathbf{1b^{IV}}$.

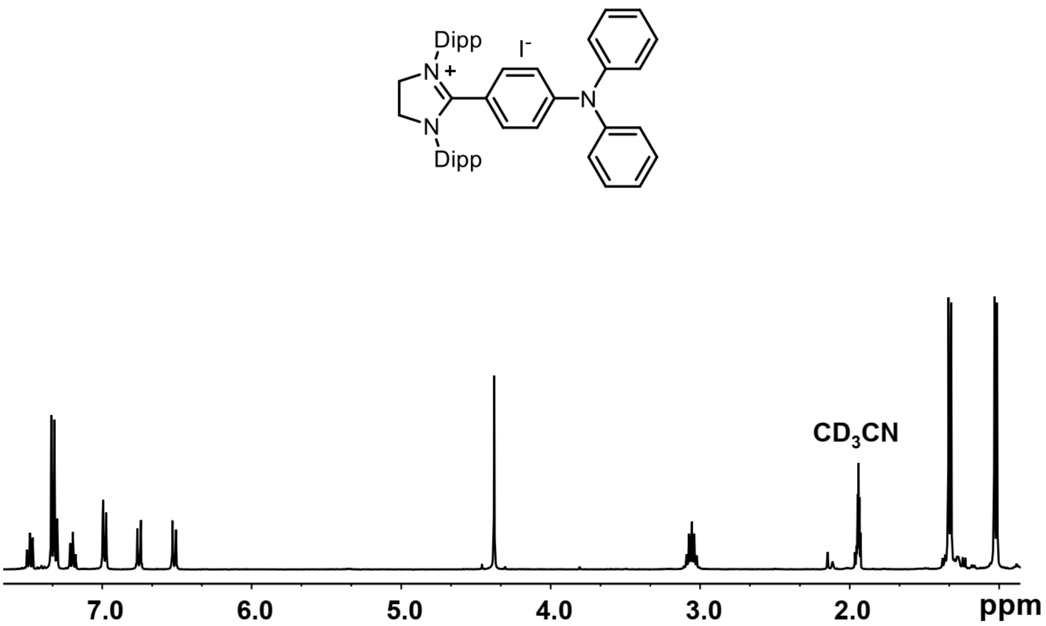

Supplementary Fig. 17. ^{1}H NMR spectrum in CD_3CN of **1c^{I}**.

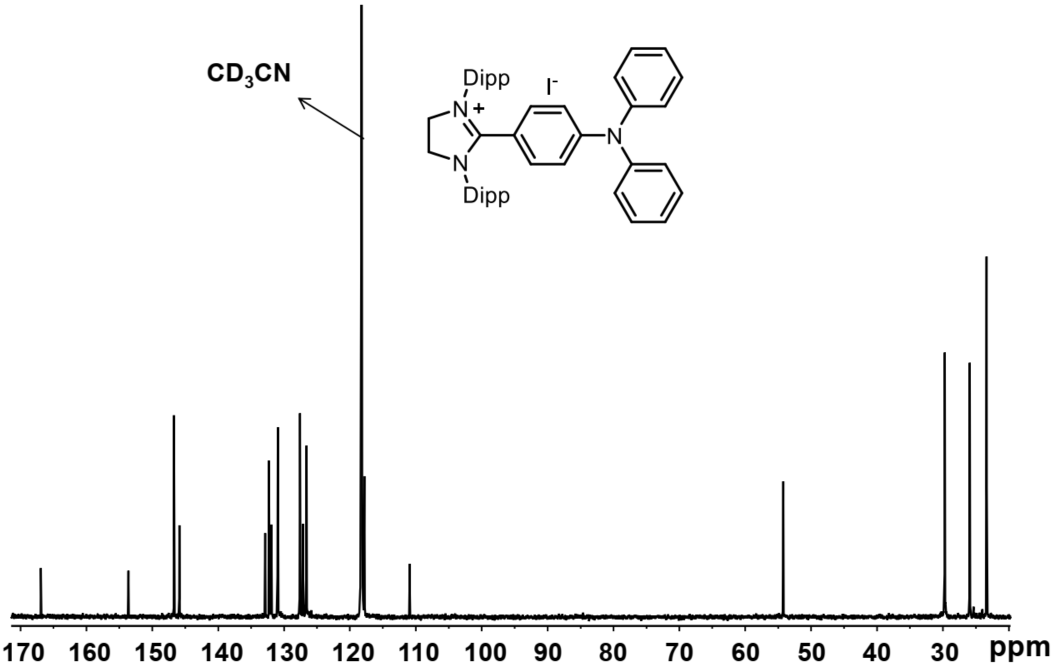

Supplementary Fig. 18. $^{13}C\{^{1}H\}$ NMR spectrum in CD_3CN of **1c^{I}**.

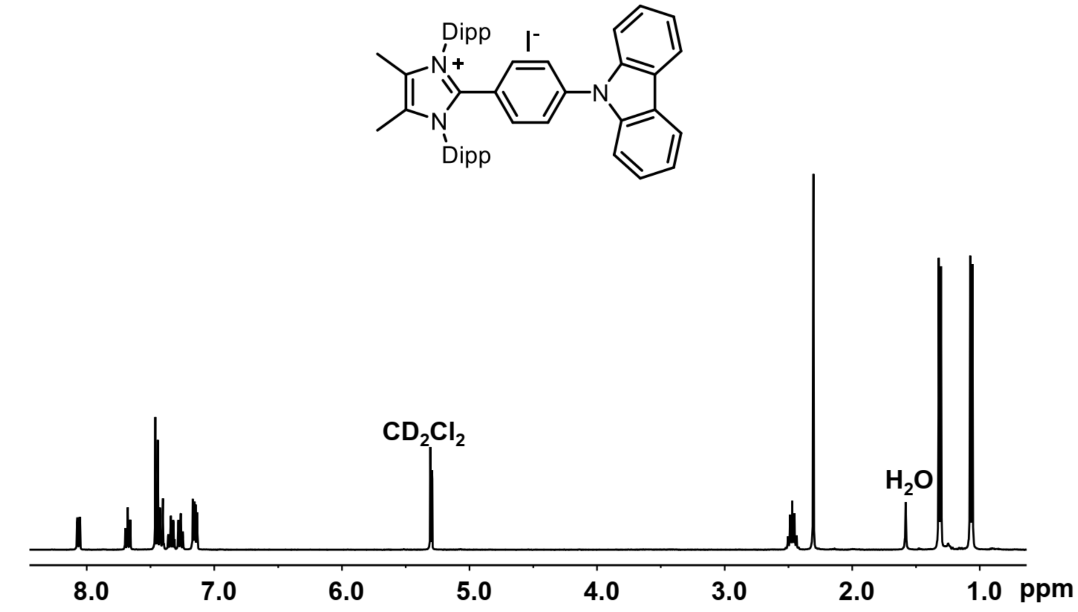

Supplementary Fig. 19. ^{1}H NMR spectrum in CD_2Cl_2 of **2a**.

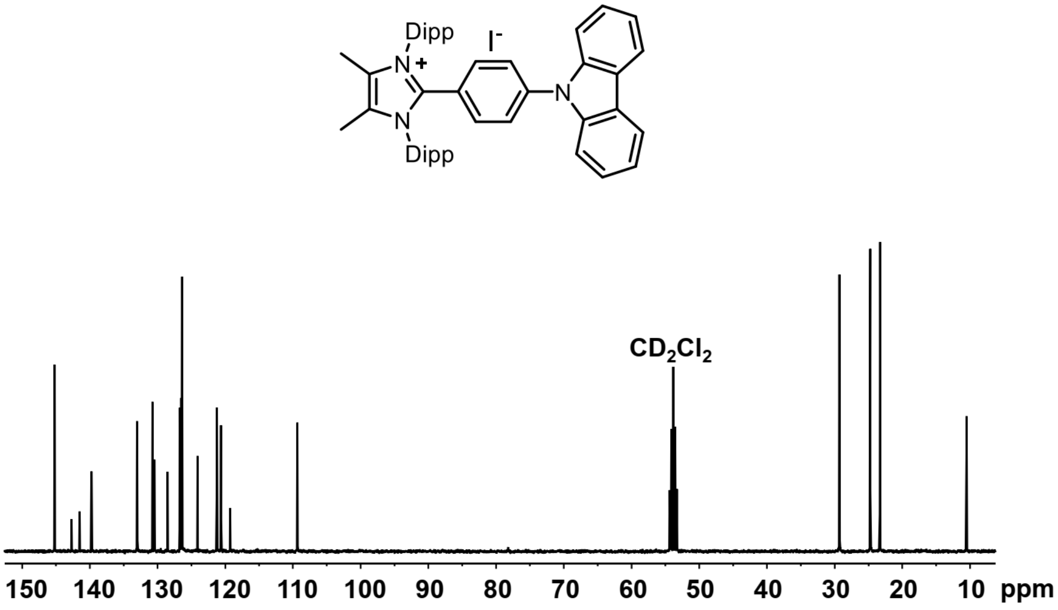

Supplementary Fig. 20. $^{13}C\{^{1}H\}$ NMR spectrum in CD_2Cl_2 of **2a**.

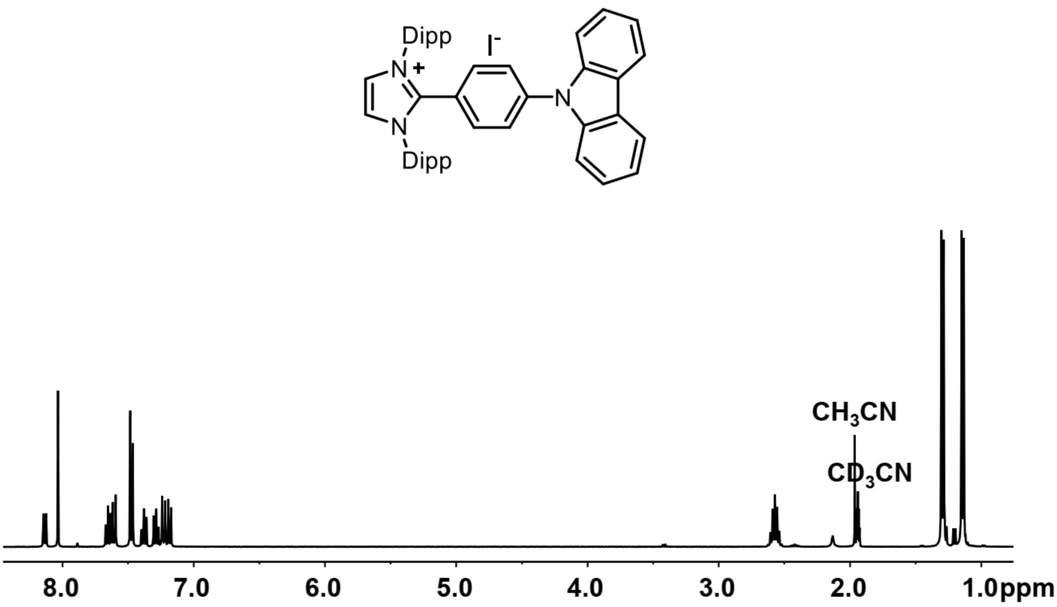

Supplementary Fig. 21. ^{1}H NMR spectrum in CD_3CN of **2b**.

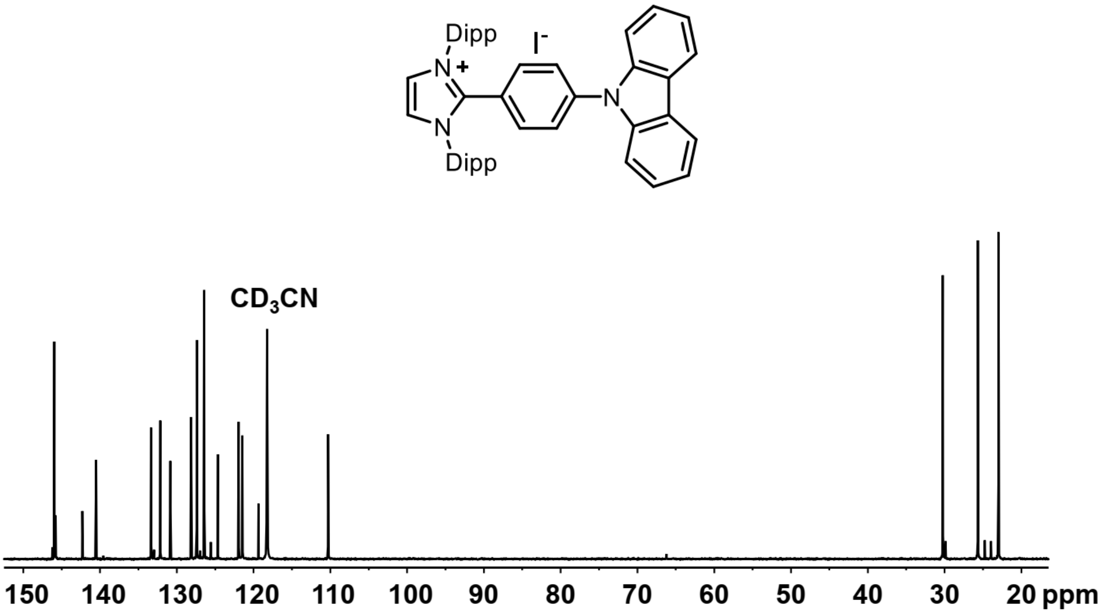

Supplementary Fig. 22. $^{13}C\{^{1}H\}$ NMR spectrum in CD_3CN of **2b**.

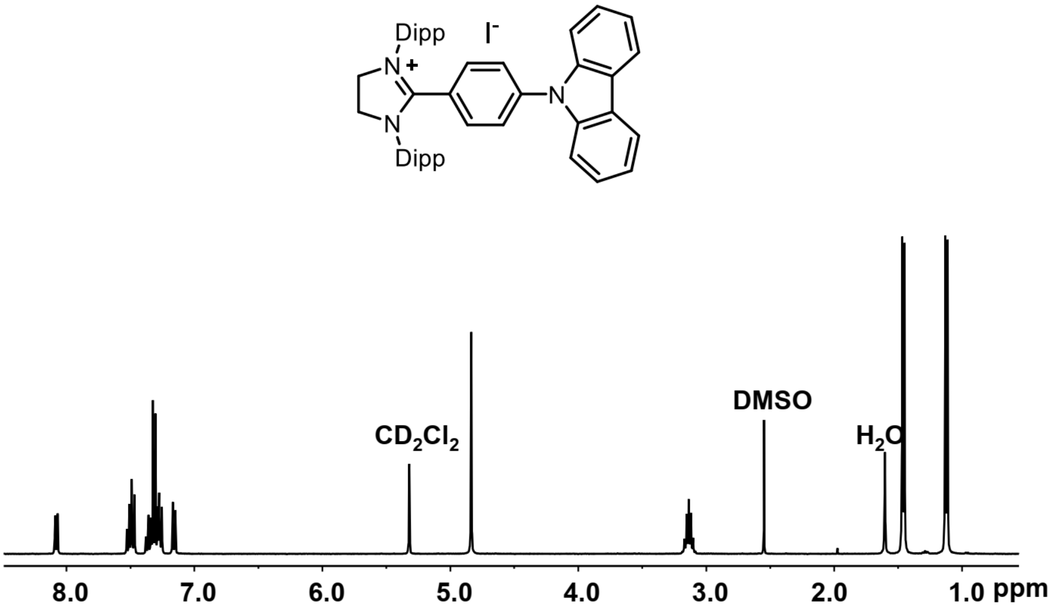

Supplementary Fig. 23. ^{1}H NMR spectrum in CD_2Cl_2 of **2c**.

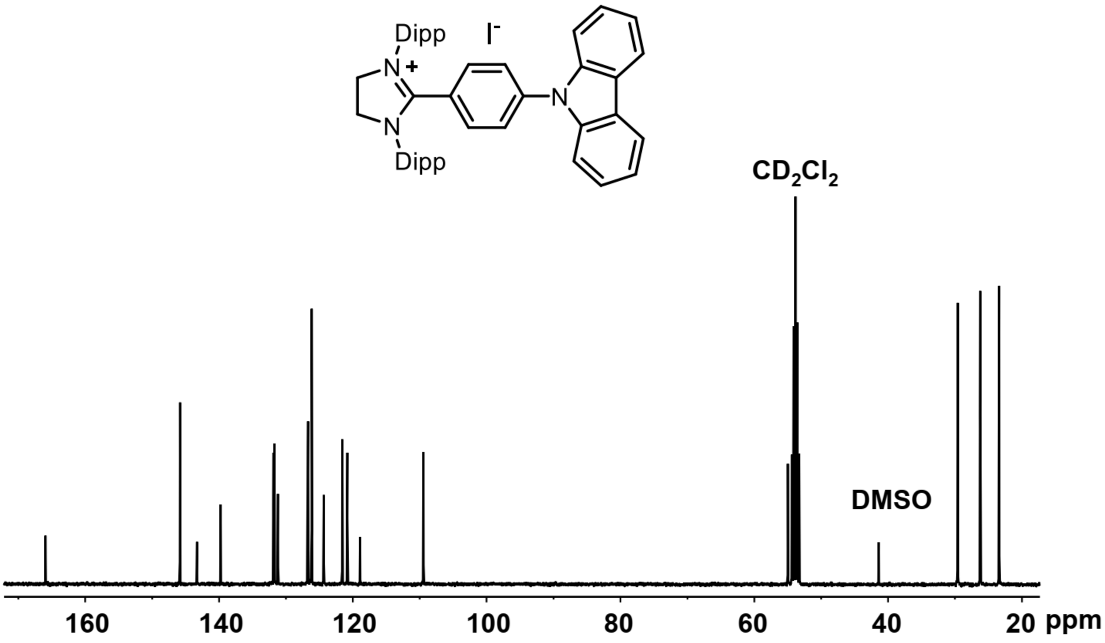

Supplementary Fig. 24. $^{13}C\{^{1}H\}$ NMR spectrum in CD_2Cl_2 of **2c**.

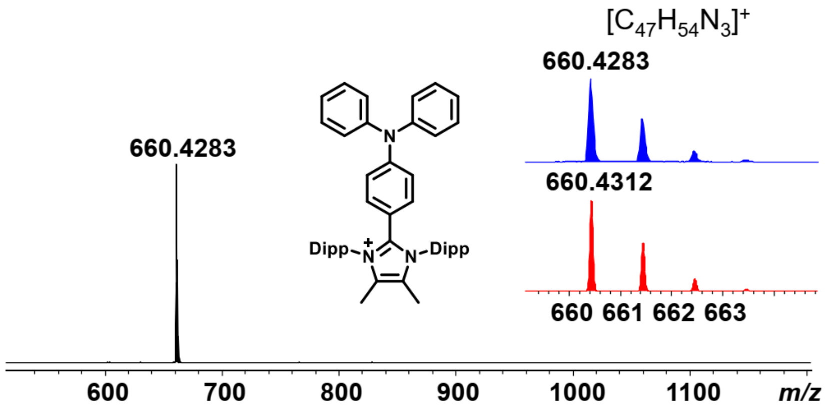

Supplementary Fig. 25. HR-ESI mass spectrum of **1a**$^{\mathbf{I}}$, inset experimental (blue) and calculated (red) isotope distributions for peaks corresponding to $[C_{47}H_{54}N_3]^+$.

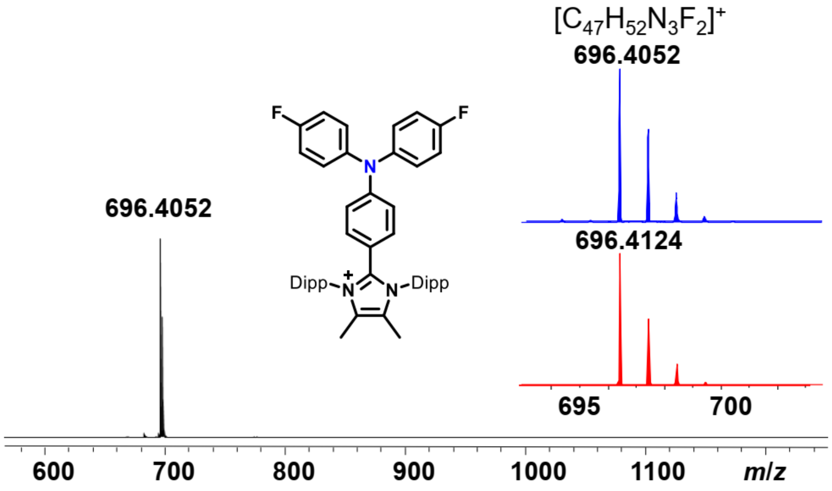

Supplementary Fig. 26. HR-ESI mass spectrum of **1a**$^{\mathbf{II}}$, inset experimental (blue) and calculated (red) isotope distributions for peaks corresponding to $[C_{47}H_{52}N_3F_2]^+$.

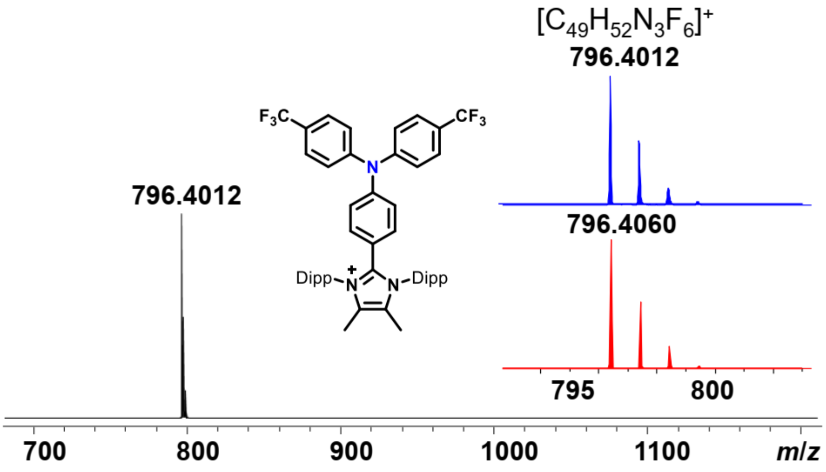

Supplementary Fig. 27. HR-ESI mass spectrum of **1a**$^{\mathbf{III}}$, inset experimental (blue) and calculated (red) isotope distributions for peaks corresponding to $[C_{49}H_{52}N_3F_6]^+$.

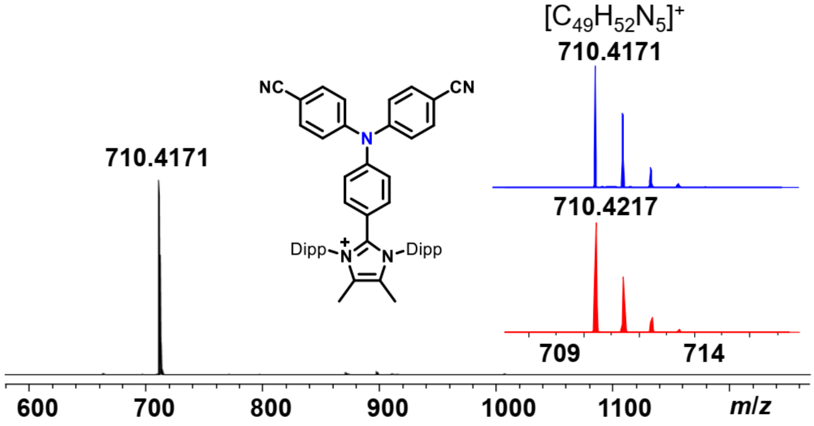

Supplementary Fig. 28. HR-ESI mass spectrum of **1a**^{IV}, inset experimental (blue) and calculated (red) isotope distributions for peaks corresponding to $[C_{49}H_{52}N_5]^+$.

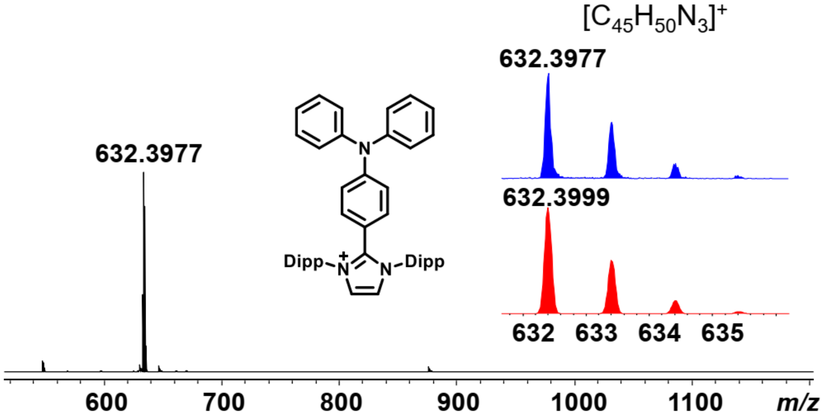

Supplementary Fig. 29. HR-ESI mass spectrum of **1b**^{I}, inset experimental (blue) and calculated (red) isotope distributions for peaks corresponding to $[C_{45}H_{50}N_3]^+$.

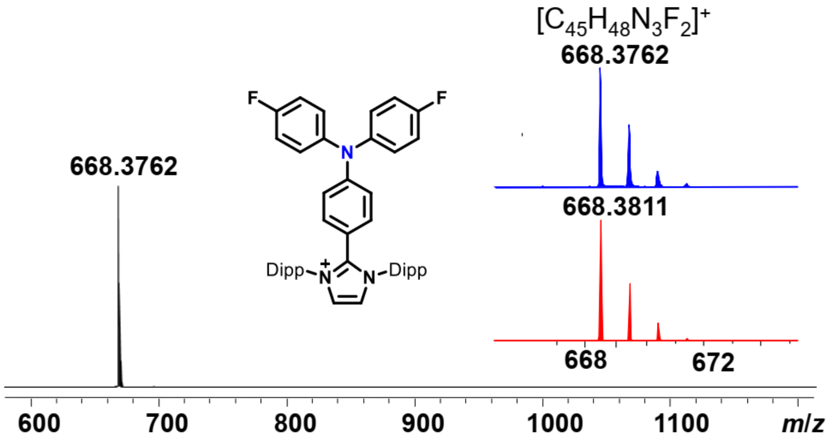

Supplementary Fig. 30. HR-ESI mass spectrum of **1b**^{II}, inset experimental (blue) and calculated (red) isotope distributions for peaks corresponding to $[C_{45}H_{49}N_3F_2]^+$.

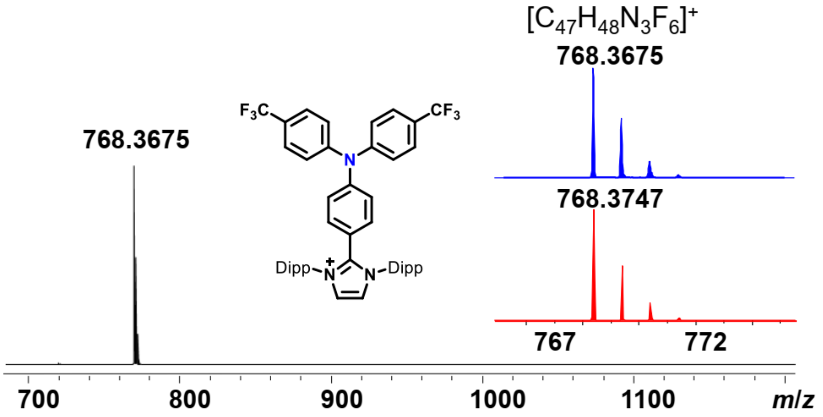

Supplementary Fig. 31. HR-ESI mass spectrum of **1b**$^{\mathbf{III}}$, inset experimental (blue) and calculated (red) isotope distributions for peaks corresponding to $[C_{47}H_{48}N_3F_2]^+$.

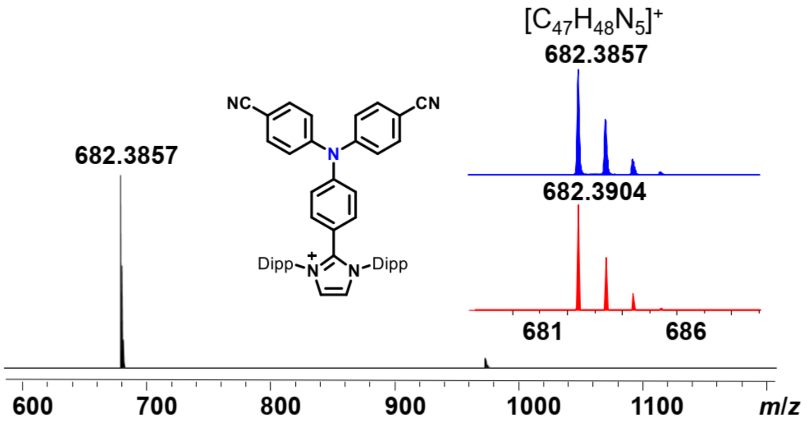

Supplementary Fig. 32. HR-ESI mass spectrum of **1b**$^{\mathbf{IV}}$, inset experimental (blue) and calculated (red) isotope distributions for peaks corresponding to $[C_{47}H_{48}N_5]^+$.

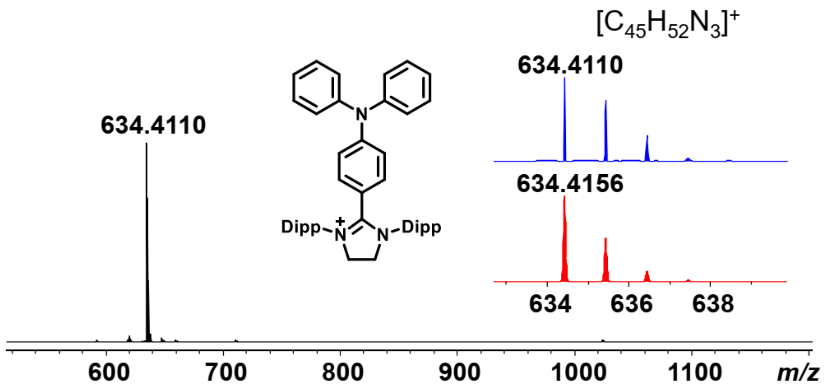

Supplementary Fig. 33. HR-ESI mass spectrum of **1c**$^{\mathbf{I}}$, inset experimental (blue) and calculated (red) isotope distributions for peaks corresponding to $[C_{45}H_{52}N_3]^+$.

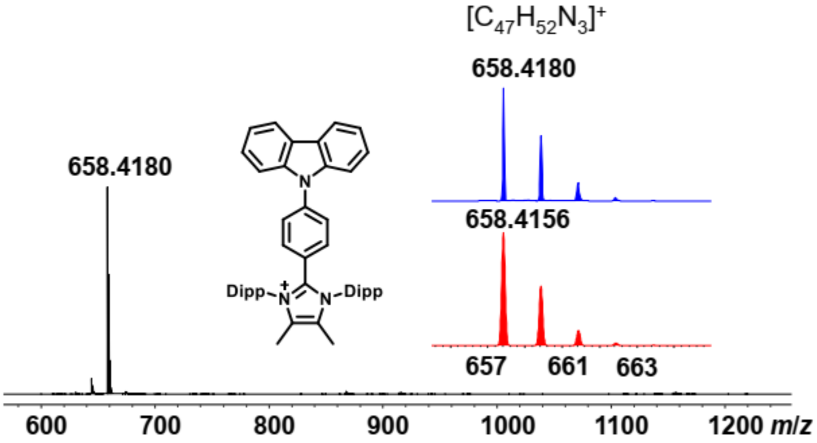

Supplementary Fig. 34. HR-ESI mass spectrum of **2a**, inset experimental (blue) and calculated (red) isotope distributions for peaks corresponding to $[C_{47}H_{52}N_3]^+$.

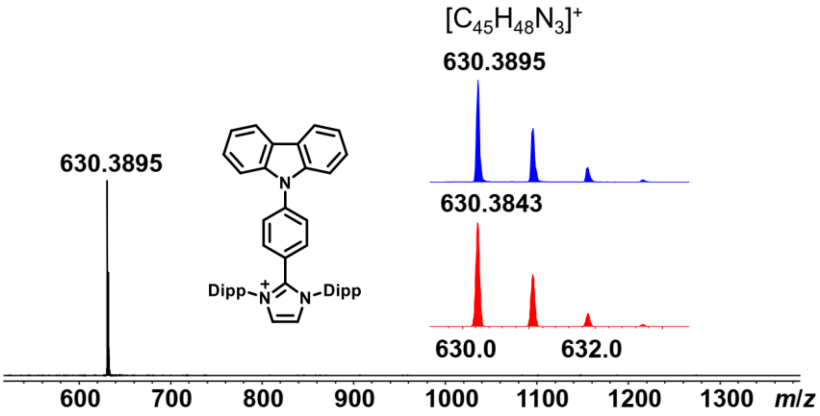

Supplementary Fig. 35. HR-ESI mass spectrum of **2b**, inset experimental (blue) and calculated (red) isotope distributions for peaks corresponding to $[C_{45}H_{48}N_3]^+$.

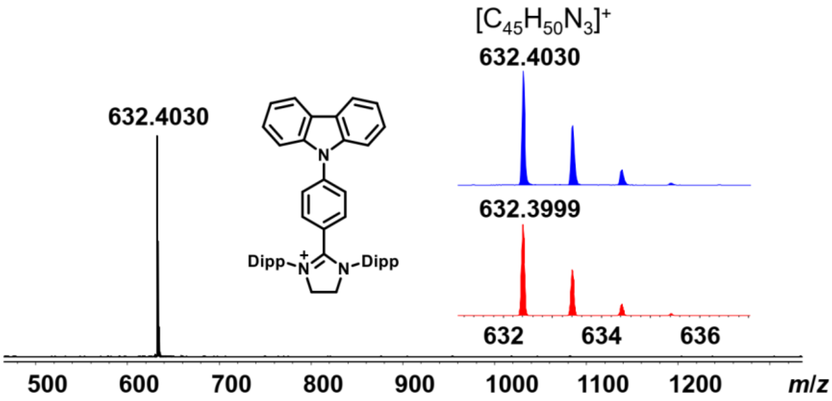

Supplementary Fig. 36. HR-ESI mass spectrum of **2c**, inset experimental (blue) and calculated (red) isotope distributions for peaks corresponding to $[C_{45}H_{50}N_3]^+$.

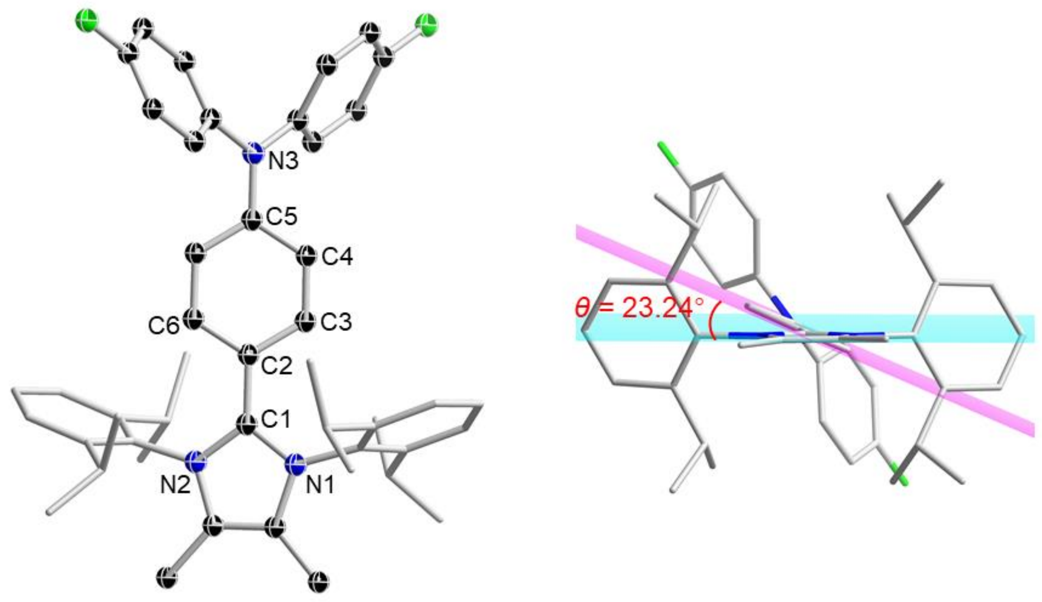

Supplementary Fig. 37. X-ray solid-state structures of $\mathbf{1a^{II}}$. Hydrogen atoms and counter anion were omitted for clarity. Selected bond lengths [Å] and bond angles [°]: C1–N1 1.358, C1–N2 1.358, C1–C2 1.467, C2–C3 1.408, C3-C4 1.384, C4-C5 1.396, C5-N3 1.404, N1-C1-N2 105.46.

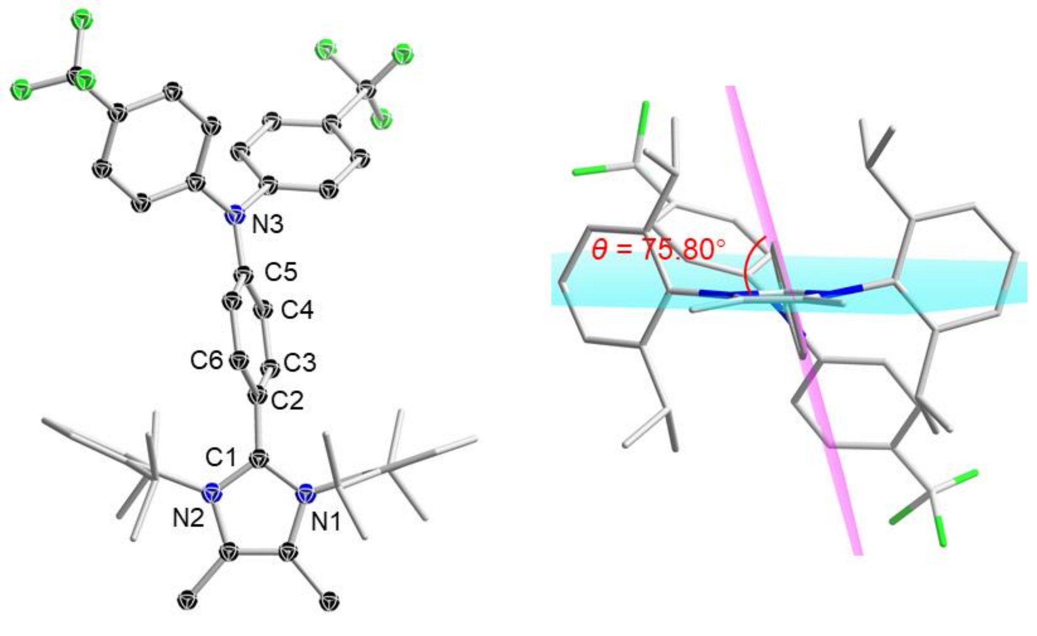

Supplementary Fig. 38. X-ray solid-state structures of $\mathbf{1a^{III}}$. Hydrogen atoms and counter anion were omitted for clarity. Selected bond lengths [Å] and bond angles [°]: C1–N1 1.342, C1–N2 1.350, C1–C2 1.469, C2–C3 1.390, C3-C4 1.384, C4-C5 1.375, C5-N3 1.418, N1-C1-N2 107.03.

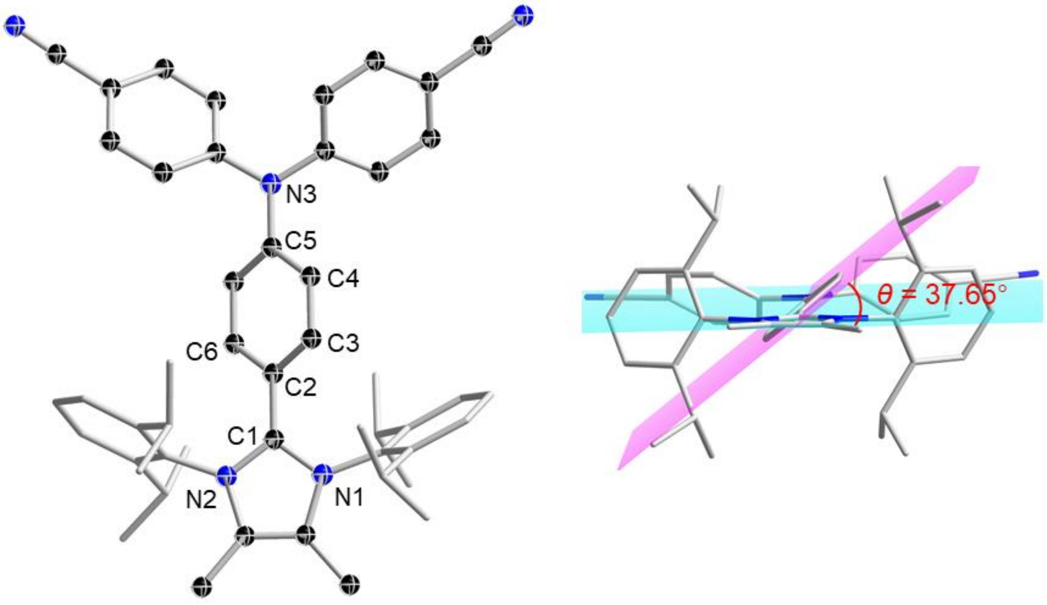

Supplementary Fig. 39. X-ray solid-state structures of **1a^{IV}**. Hydrogen atoms and counter anion were omitted for clarity. Selected bond lengths [Å] and bond angles [°]: C1–N1 1.346, C1–N2 1.346, C1–C2 1.469, C2–C3 1.395, C3-C4 1.382, C4-C5 1.389, C5-N3 1.415, N1-C1-N2 106.72.

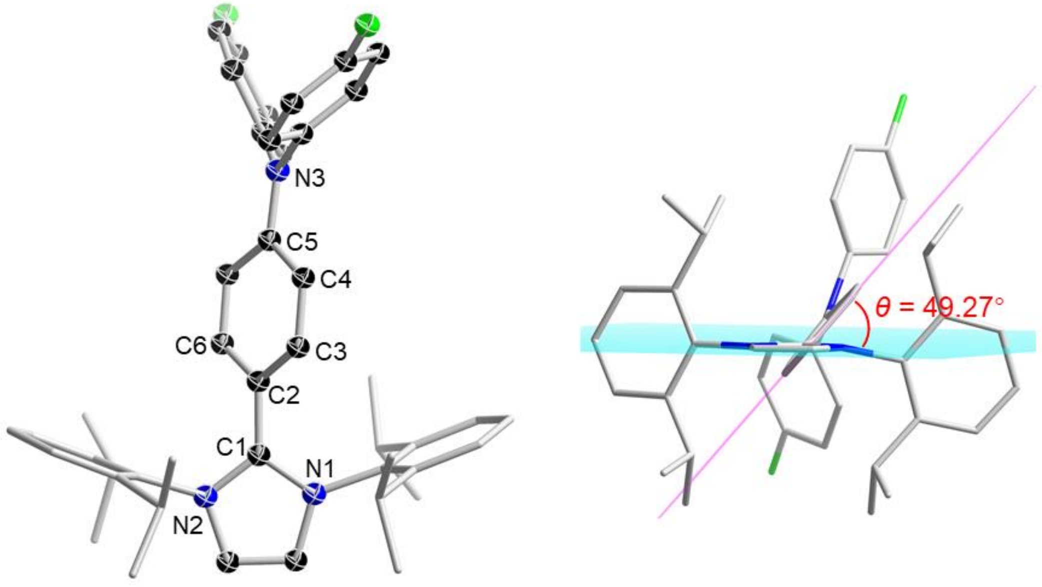

Supplementary Fig. 40. X-ray solid-state structures of **1b^{II}**. Hydrogen atoms and counter anion were omitted for clarity. Selected bond lengths [Å] and bond angles [°]: C1–N1 1.342, C1–N2 1.347, C1–C2 1.466, C2–C3 1.392, C3-C4 1.378, C4-C5 1.402, C5-N3 1.392, N1-C1-N2 106.99.

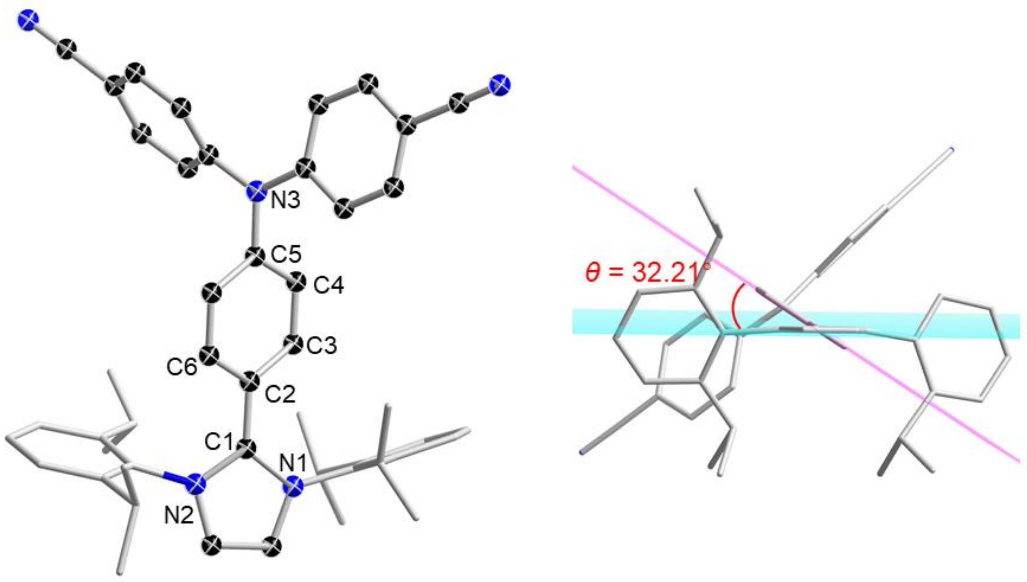

Supplementary Fig. 41. X-ray solid-state structures of $\mathbf{1b^{IV}}$. Hydrogen atoms and counter anion were omitted for clarity. Selected bond lengths [Å] and bond angles [°]: C1–N1 1.334, C1–N2 1.348, C1–C2 1.477, C2–C3 1.391, C3-C4 1.387, C4-C5 1.374, C5-N3 1.436, N1-C1-N2 106.14.

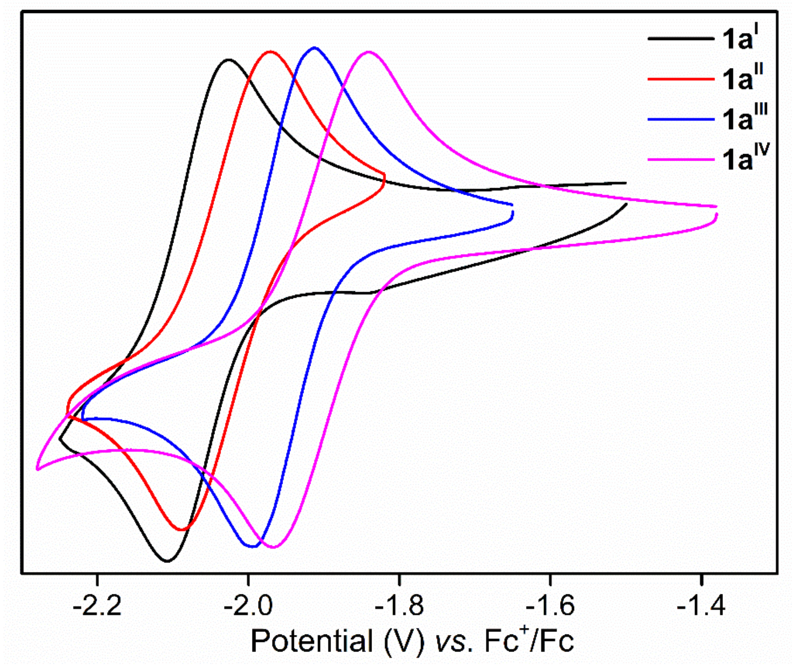

Supplementary Fig. 42. Cyclic voltammograms of $\mathbf{1a^{I}}$–$\mathbf{1a^{IV}}$ in CH_3CN with 0.1 M Bu_4NPF_6 at a scan rate of 100 mV s^{-1}.

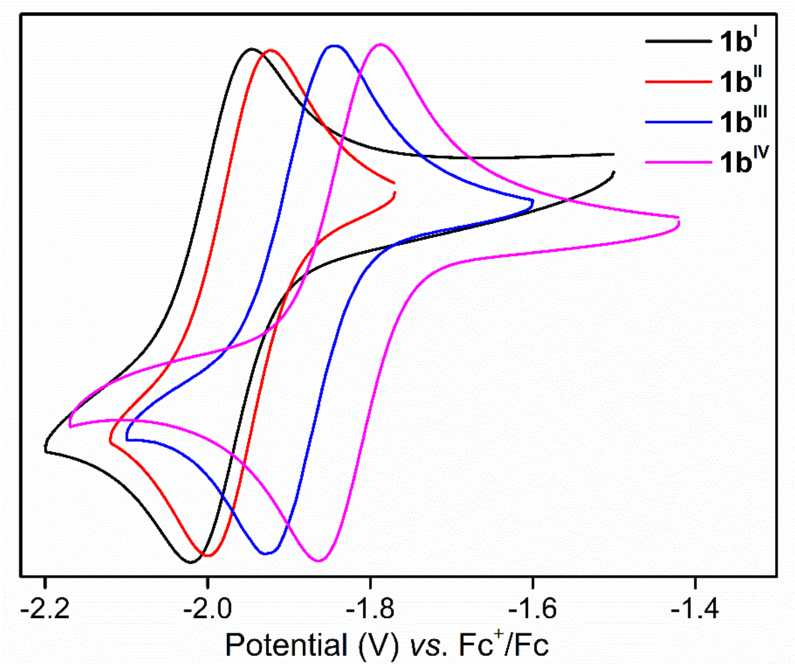

Supplementary Fig. 43. Cyclic voltammograms of **$1b^{I}$–$1b^{IV}$** in CH_3CN with 0.1 M Bu_4NPF_6 at a scan rate of 100 mV s^{-1}.

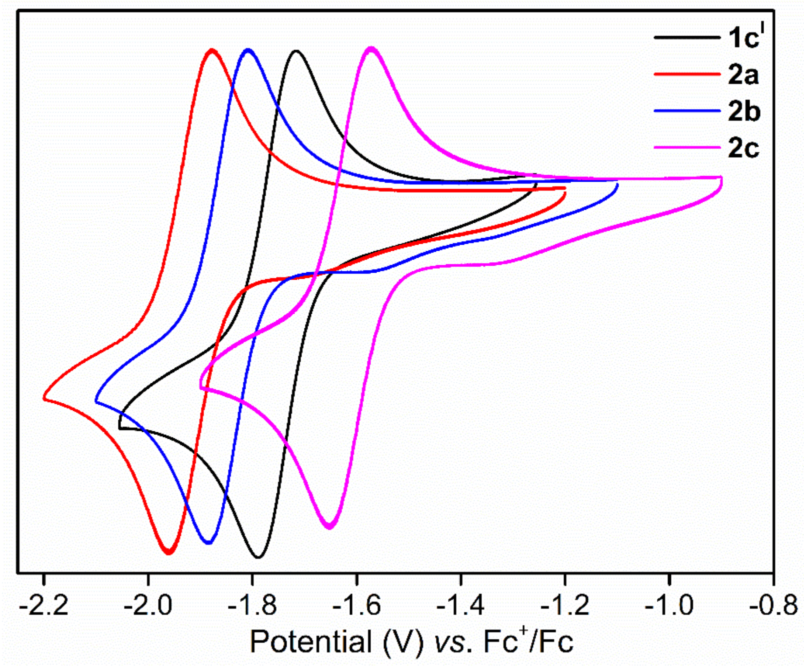

Supplementary Fig. 44. Cyclic voltammograms of **$1c^{I}$** and **2a–c** in CH_3CN with 0.1 M Bu_4NPF_6 at a scan rate of 100 mV s^{-1}.

3. Synthesis and characterization of 3a-c and 4a-c

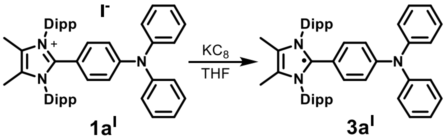

$1a^I$ $3a^I$

3a^I: KC_8 (40.5 mg, 0.3 mmol) was added to a THF (10.0 mL) solution of precursor salt **1a^I** (227.9 mg, 0.3 mmol) at −35 ºC. The reaction mixture was brought to room temperature and further stirred for 4h. Filtration through a plug of Celite afforded a blue solution. The volatiles was removed, extraction with hexane and removal of volatiles afforded **3a^I** as a black powder. Single crystals were grown by cooling a saturated hexane solution of **3a^I** at −30 °C. Yield: 168.9 mg, 89%. Elemental analysis (%) for $C_{47}H_{54}N_3$: calcd: C 85.41, H 8.24, N 6.36; found: C 83.37, H 7.92, N 5.98. UV−vis (THF, λ (nm), ε (M^{-1} cm^{-1})): 219 (36364), 290 (18298), 348 (22364), 462 (2436). IR (cm^{-1}): λ = 2975, 2932, 1643, 1584, 1528, 1492, 1462, 1331, 1276, 1191, 1066, 818, 769, 709, 505.

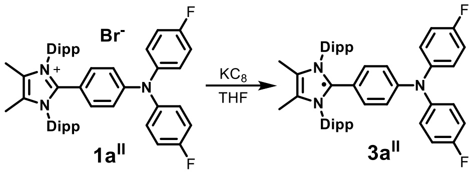

$1a^{II}$ $3a^{II}$

3a^II: KC_8 (40.5 mg, 0.3 mmol) was added to a THF (10.0 mL) solution of precursor salt **1a^II** (233.1 mg, 0.3 mmol) at −35 ºC. The reaction mixture was brought to room temperature and further stirred for 4h. Filtration through a plug of Celite afforded a blue solution. The volatiles were removed, extraction with hexane and removal of volatiles afforded **3a^II** as a black powder. Yield: 198.4 mg, 95%. Elemental analysis (%) for $C_{47}H_{52}N_3F_2$: calcd: C 81.00, H 7.52, N 6.03; found: C 78.34, H 6.72, N 5.34. UV−vis (THF, λ (nm), ε (M^{-1} cm^{-1})): 223 (97731), 295 (60888), 327 (66174), 471 (2957). IR (cm^{-1}): λ = 2982, 2942, 2865, 1641, 1590, 1529, 1487, 1458, 1331, 1304, 1270, 1192, 1061, 815, 765, 701, 534.

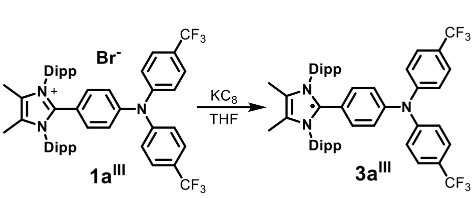

$1a^{III}$ $3a^{III}$

3a^III: KC_8 (40.5 mg, 0.3 mmol) was added to a THF (10.0 mL) solution of precursor salt **1a^III** (263.1 mg, 0.3 mmol) at −35 ºC. The reaction mixture was brought to room temperature and further stirred for 4h. Filtration through a plug of Celite afforded a blue solution. The volatiles were removed,

extraction with hexane and removal of volatiles afforded **3a^{III}** as a black powder. Yield: 219.8 mg, 92%. Elemental analysis (%) for $C_{49}H_{52}N_3F_6$: calcd: C 73.85, H 6.58, N 5.27; found: C 71.18, H 5.97, N 4.74. UV−vis (THF, λ (nm), ε (M^{-1} cm^{-1})): 215 (49389), 315 (17576), 356 (15490), 429 (3442), 490 (2106). IR (cm^{-1}): λ = 2978, 2930, 2871, 1642, 1590, 1529, 1491, 1457, 1332, 1308, 1278, 1193, 1045, 804, 702, 515.

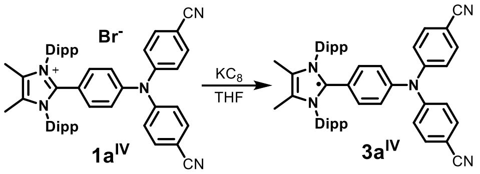

3a^{IV}: KC_8 (40.5 mg, 0.3 mmol) was added to a THF (10.0 mL) solution of precursor salt **1a^{IV}** (237.1 mg, 0.3 mmol) at −35 ºC. The reaction mixture was brought to room temperature and further stirred for 4h. Filtration through a plug of Celite afforded a blue solution. The volatiles were removed, extraction with hexane and removal of volatiles afforded **3a^{IV}** as a black powder. Yield: 202 mg, 95%. Elemental analysis (%) for $C_{49}H_{52}N_5$: calcd: C 82.78, H 7.37, N 9.85; found: C 80.32, H 6.83, N 8.79. UV−vis (THF, λ (nm), ε (M^{-1} cm^{-1})): 212 (22092), 343 (10706), 499 (982). IR (cm^{-1}): λ = 2979, 2934, 2867, 1592, 1527, 1475, 1443, 1328, 1302, 1270, 1185, 1062, 932, 802, 757, 705, 518, 452.

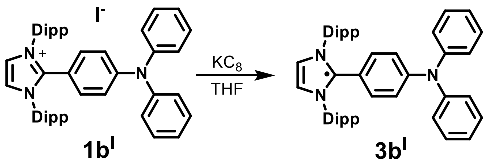

3b^{I}: KC_8 (40.5 mg, 0.3 mmol) was added to a THF (10.0 mL) solution of precursor salt **1b^{I}** (228.5 mg, 0.3 mmol) at −35 ºC. The reaction mixture was brought to room temperature and further stirred for 4h. Filtration through a plug of Celite afforded a blue solution. The volatiles was removed, extraction with hexane and removal of volatiles afforded **3b^{I}** as a black powder. Single crystals were grown by cooling a saturated hexane solution of **3b^{I}** at −30 °C. Yield: 175.2 mg, 92%. Elemental analysis (%) for $C_{45}H_{50}N_3$: calcd: C 85.40, H 7.96, N 6.64; found: C 83.21, H 7.04, N 5.97. UV−vis (THF, λ (nm), ε (M^{-1} cm^{-1})): 219 (39393), 296 (20729), 346 (17731), 478 (2498). IR (cm^{-1}): λ = 2965, 2938, 2845, 1628, 1602, 1532, 1451, 1361, 1331, 1224, 1168, 1062, 841, 812, 758, 726, 655, 453.

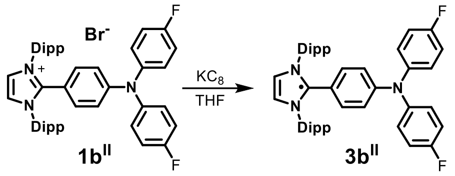

3b^II: KC_8 (40.5 mg, 0.3 mmol) was added to a THF (10.0 mL) solution of precursor salt **1b^II** (224.7 mg, 0.3 mmol) at −35 ºC. The reaction mixture was brought to room temperature and further stirred for 4h. Filtration through a plug of Celite afforded a blue solution. The volatiles were removed, extraction with hexane and removal of volatiles afforded **3b^II** as a black powder. Yield: 178.4 mg, 89%. Elemental analysis (%) for $C_{45}H_{48}N_3F_2$: calcd: C 80.80, H 7.23, N 6.28; found: C 79.12, H 6.56, N 5.87. UV−vis (THF, λ (nm), ε (M^{-1} cm^{-1})): 213 (45882), 292 (13131), 327 (14339), 465 (1775). IR (cm^{-1}): λ = 2962, 2918, 2872, 1635, 1605, 1525, 1443, 1371, 1329, 1224, 1173, 1058, 835, 754, 731, 653, 457.

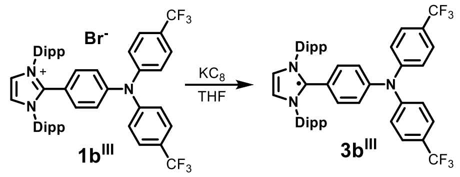

3b^III: KC_8 (40.5 mg, 0.3 mmol) was added to a THF (10.0 mL) solution of precursor salt **1b^III** (254.7 mg, 0.3 mmol) at −35 ºC. The reaction mixture was brought to room temperature and further stirred for 4h. Filtration through a plug of Celite afforded a blue solution. The volatiles was removed, extraction with hexane and removal of volatiles afforded **3b^III** as a black powder. Single crystals were grown by cooling a saturated hexane solution of **3b^III** at −30 °C. Yield: 204.2 mg, 89%. Elemental analysis (%) for $C_{47}H_{48}N_3F_6$: calcd: C 73.42, H 6.29, N 5.46; found: C 71.89, H 5.78, N 4.72. UV−vis (THF, λ (nm), ε (M^{-1} cm^{-1})): 217 (64497), 318 (32437), 351 (27567), 478 (4614). IR (cm^{-1}): λ = 2968, 2932, 2892, 1635, 1532, 1451, 1362, 1332, 1228, 1170, 1061, 832, 802, 751, 733, 652, 448.

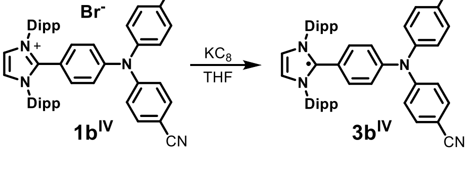

3b^IV: KC_8 (40.5 mg, 0.3 mmol) was added to a THF (10.0 mL) solution of precursor salt **1b^IV** (228.9 mg, 0.3 mmol) at −35 ºC. The reaction mixture was brought to room temperature and further stirred

for 4h. Filtration through a plug of Celite afforded a brown solution. The volatiles were removed, extraction with hexane and removal of volatiles afforded **3b^IV^** as a black powder. Yield: 188 mg, 92%. Elemental analysis (%) for $C_{47}H_{48}N_5$: calcd: C 82.66, H 7.08, N 10.25; found: C 80.23, H 6.34, N 9.44. UV−vis (THF, λ (nm), ε (M^{-1} cm^{-1})): 213 (34824), 313 (11435), 356 (10521), 485 (1223). IR (cm^{-1}): λ = 2978, 2885, 1589, 1527, 1446, 1332, 1276, 1187, 1043, 932, 801, 760, 693, 523, 438.

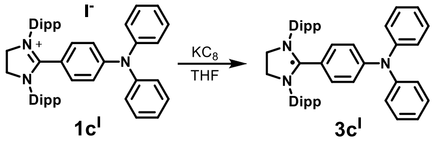

1c^I^ **3c^I^**

3c^I^: KC_8 (40.5 mg, 0.3 mmol) was added to a THF (10.0 mL) solution of precursor salt **1c^I^** (236.3 mg, 0.3 mmol) at −35 ºC. The reaction mixture was brought to room temperature and further stirred for 4h. Filtration through a plug of Celite afforded a brown solution. The volatiles was removed, extraction with hexane and removal of volatiles afforded **3c^I^** as a black powder. Single crystals were grown by cooling a saturated hexane solution of **3c^I^** at −30 °C. Yield: 188.4 mg, 95%. Elemental analysis (%) for $C_{45}H_{52}N_3$: calcd: C 85.13, H 8.26, N 6.62; found: C 83.34, H 7.15, N 6.01. UV−vis (THF, λ (nm), ε (M^{-1} cm^{-1})): 218 (45454), 299 (27227), 396 (13609), 550 (1514). IR (cm^{-1}): λ = 2972, 2870, 1604, 1523, 1450, 1328, 1288, 1187, 1043, 932, 801, 762, 689, 524, 445.

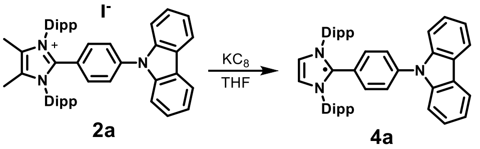

2a **4a**

4a: KC_8 (40.5 mg, 0.3 mmol) was added to a THF (10.0 mL) solution of precursor salt **2a** (227.3 mg, 0.3 mmol) at −35 ºC. The reaction mixture was brought to room temperature and further stirred for 4h. Filtration through a plug of Celite afforded a blue solution. The volatiles was removed, extraction with hexane and removal of volatiles afforded **4a** as a black powder. Single crystals were grown by cooling a saturated hexane solution of **4a** at −30 °C. Yield: 174.1 mg, 92%. Elemental analysis (%) for $C_{47}H_{52}N_3$: calcd: C 85.67, H 7.95, N 6.38; found: C 83.76, H 7.38, N 5.97. UV−vis (THF, λ (nm), ε (M^{-1} cm^{-1})): 244 (44662), 296 (22093), 336 (16434), 392 (16470), 468 (5408). IR (cm^{-1}): λ = 2972, 2921, 2865, 1639, 1604, 1529, 1452, 1365, 1333, 1219, 1168, 1061, 835, 807, 751, 727, 649, 463.

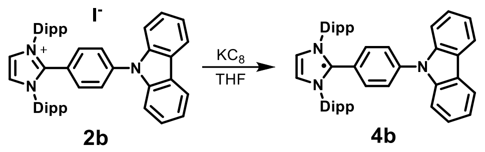

4b: KC_8 (40.5 mg, 0.3 mmol) was added to a THF (10.0 mL) solution of precursor salt **2b** (227.9 mg, 0.3 mmol) at −35 ºC. The reaction mixture was brought to room temperature and further stirred for 4h. Filtration through a plug of Celite afforded a blue solution. The volatiles was removed, extraction with hexane and removal of volatiles afforded **4b** as a black powder. Single crystals were grown by cooling a saturated hexane solution of **4b** at −30 °C. Yield: 170.8 mg, 90%. Elemental analysis (%) for $C_{45}H_{48}N_3$: calcd: C 85.67, H 7.67, N 6.66; found: C 83.23, H 7.03, N 6.34. UV−vis (THF, λ (nm), ε (M^{-1} cm^{-1})): 243 (47423), 294 (14793), 336 (12681), 467 (3072), 673 (2260). IR (cm^{-1}): λ = 2968, 2933, 2871, 1615, 1531, 1445, 1365, 1332, 1232, 1175, 1051, 847, 813, 743, 683, 465.

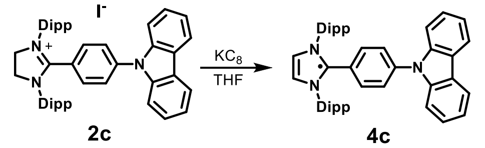

4c: KC_8 (40.5 mg, 0.3 mmol) was added to a THF (10.0 mL) solution of precursor salt **2c** (235.7 mg, 0.3 mmol) at −35 ºC. The reaction mixture was brought to room temperature and further stirred for 4h. Filtration through a plug of Celite afforded a brown solution. The volatiles was removed, extraction with hexane and removal of volatiles afforded **4c** as a black powder. Single crystals were grown by cooling a saturated hexane solution of **4c** at −30 °C. Yield: 175.9 mg, 89%. Elemental analysis (%) for $C_{45}H_{50}N_3$: calcd: C 85.40, H 7.96, N 6.64; found: C 83.45, H 7.12, N 5.98. UV−vis (THF, λ (nm), ε (M^{-1} cm^{-1})): 240 (49315), 294 (17726), 368 (11114), 516 (2590). IR (cm^{-1}): λ = 2973, 2932, 2864, 1578, 1518, 1484, 1433, 1332, 1316, 1189, 1061, 832, 763, 709, 489.

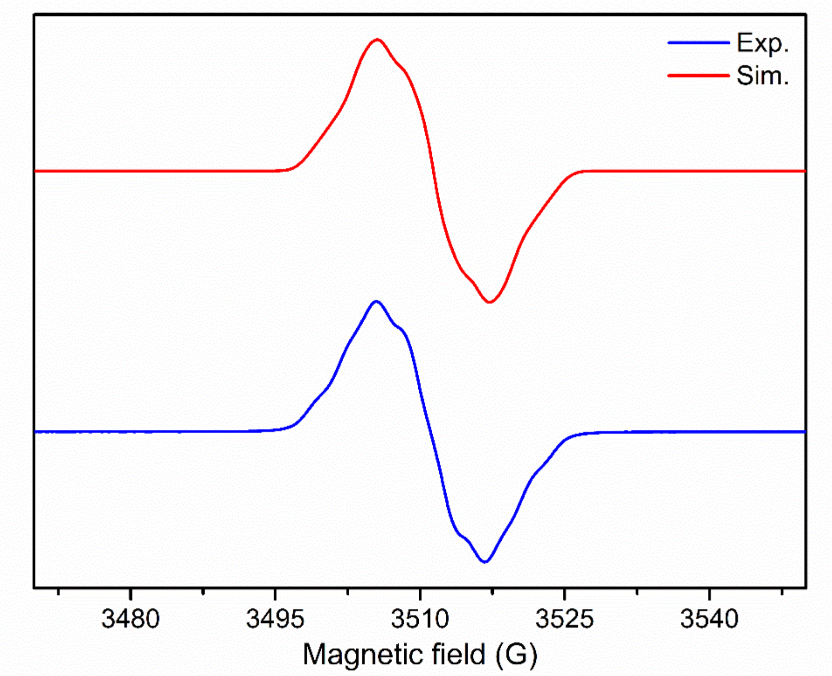

Supplementary Fig. 45. Experimental (blue) and simulated (red) EPR spectra of **3a$^{\mathbf{I}}$** in THF solution at room temperature (c = 2.0 mM). Hyperfine couplings: g = 2.0029, A(2*^{14}N): 2.62 G, A(2*^{1}H): 4.35 G, A(2*^{1}H): 1.68 G.

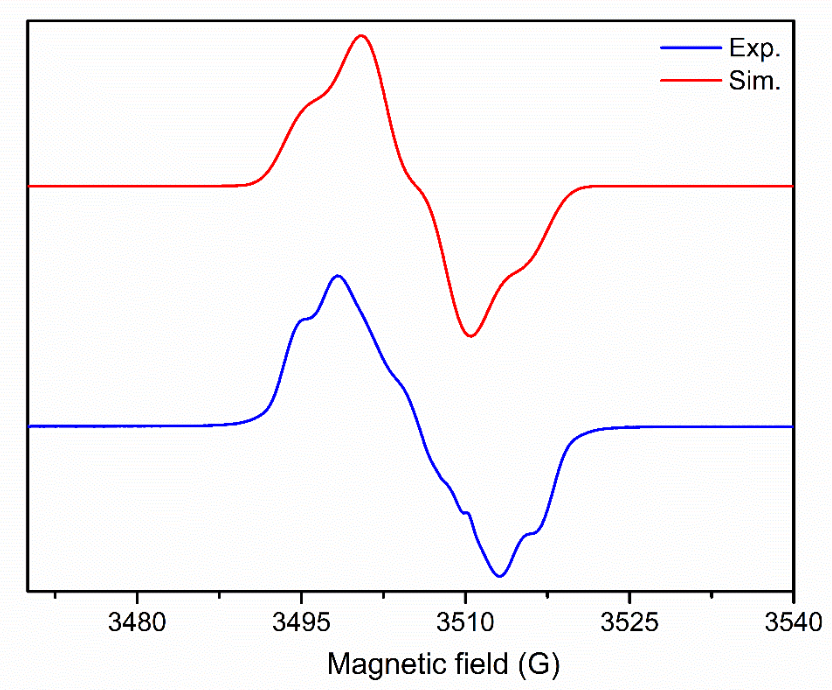

Supplementary Fig. 46. Experimental (blue) and simulated (red) EPR spectra of **3a$^{\mathbf{II}}$** in THF solution at room temperature (c = 1.8 mM). Hyperfine couplings: g = 2.0030, A(2*^{14}N): 1.50 G, A(2*^{1}H): 4.80 G, A(*^{1}H): 5.30 G.

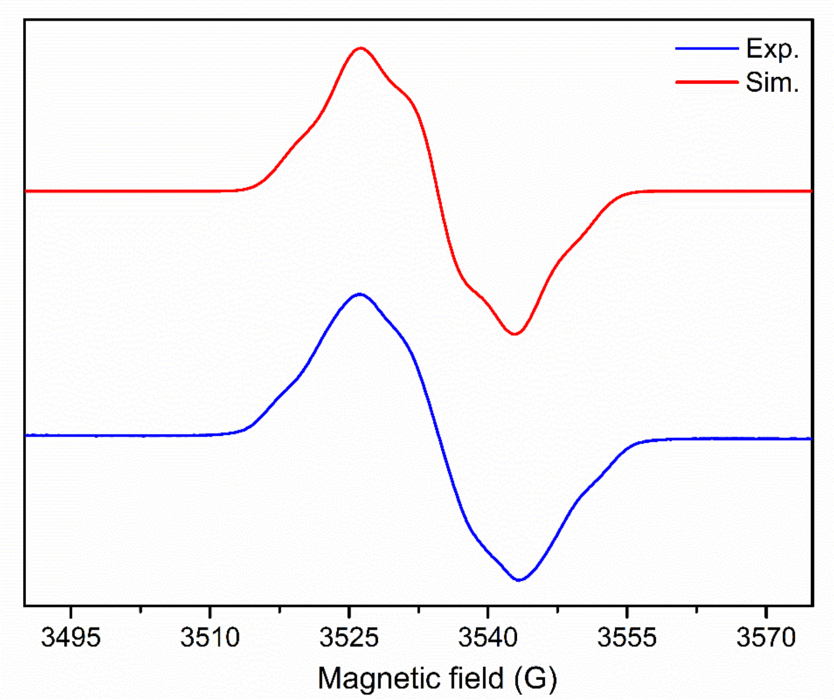

Supplementary Fig. 47. Experimental (blue) and simulated (red) EPR spectra of $\mathbf{3a}^{\mathrm{III}}$ in THF solution at room temperature (*c* = 1.8 mM). Hyperfine couplings: *g* = 2.0029, $A(2{*}^{14}N)$: 2.35 G, $A(2{*}^{1}H)$: 4.20 G.

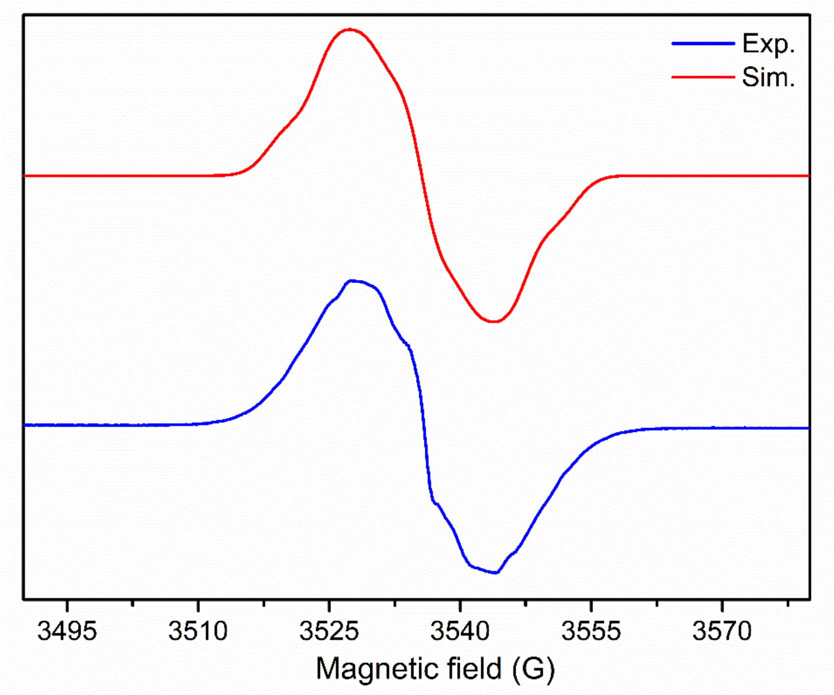

Supplementary Fig. 48. Experimental (blue) and simulated (red) EPR spectra of $\mathbf{3a}^{\mathrm{IV}}$ in THF solution at room temperature (*c* = 1.8 mM). Hyperfine couplings: *g* = 2.0028, $A(2{*}^{14}N)$: 2.65 G, $A(2{*}^{1}H)$: 3.85 G.

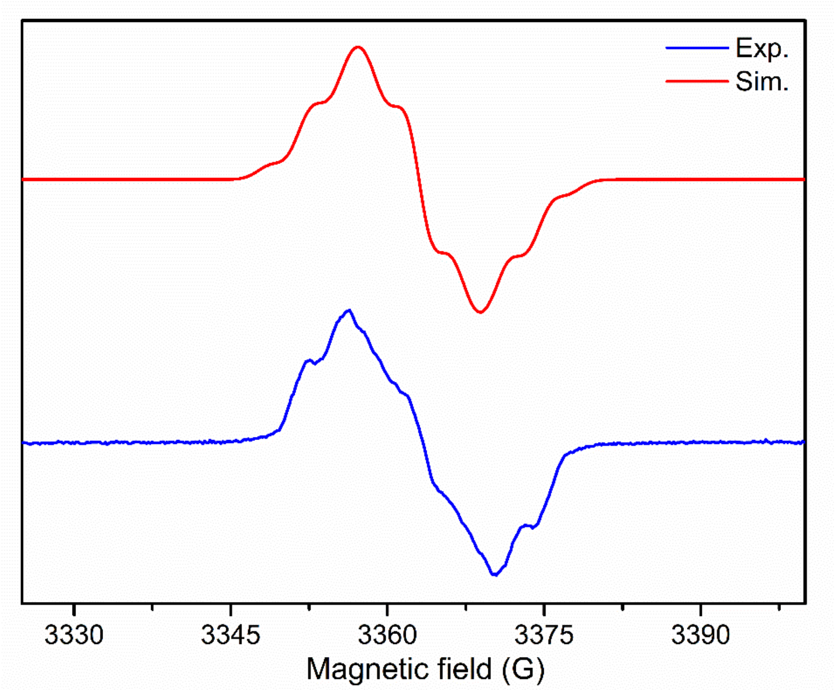

Supplementary Fig. 49. Experimental (blue) and simulated (red) EPR spectra of **3b^{I}** in THF solution at room temperature (c = 1.8 mM). Hyperfine couplings: g = 2.0027, A(2*^{14}N): 2.52 G, A(2*^{1}H): 4.02 G, A(2*^{1}H): 3.85 G.

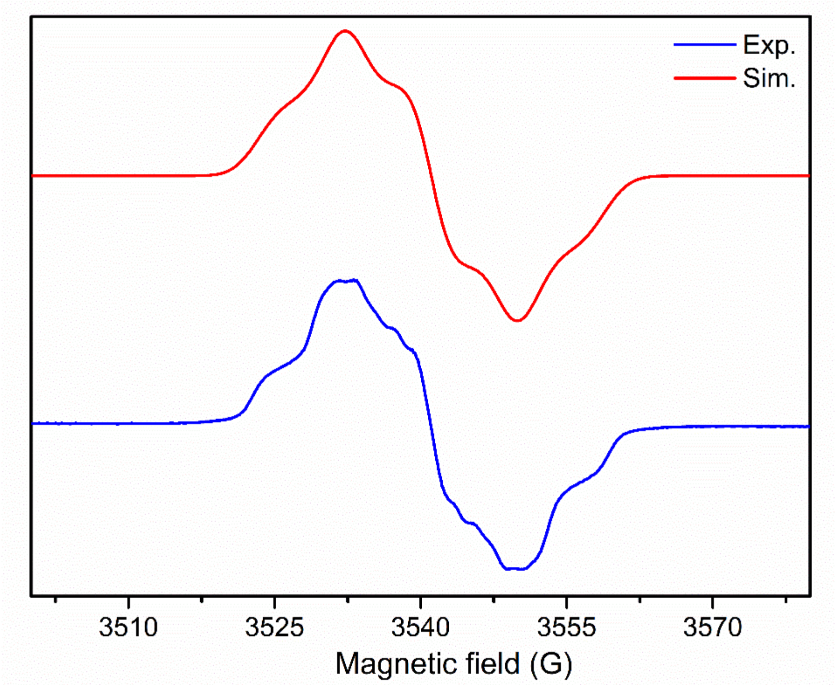

Supplementary Fig. 50. Experimental (blue) and simulated (red) EPR spectra of **3b^{II}** in THF solution at room temperature (c = 1.8 mM). Hyperfine couplings: g = 2.0028, A(2*^{14}N): 2.25 G, A(2*^{1}H): 5.05 G, A(2*^{1}H): 0.95 G.

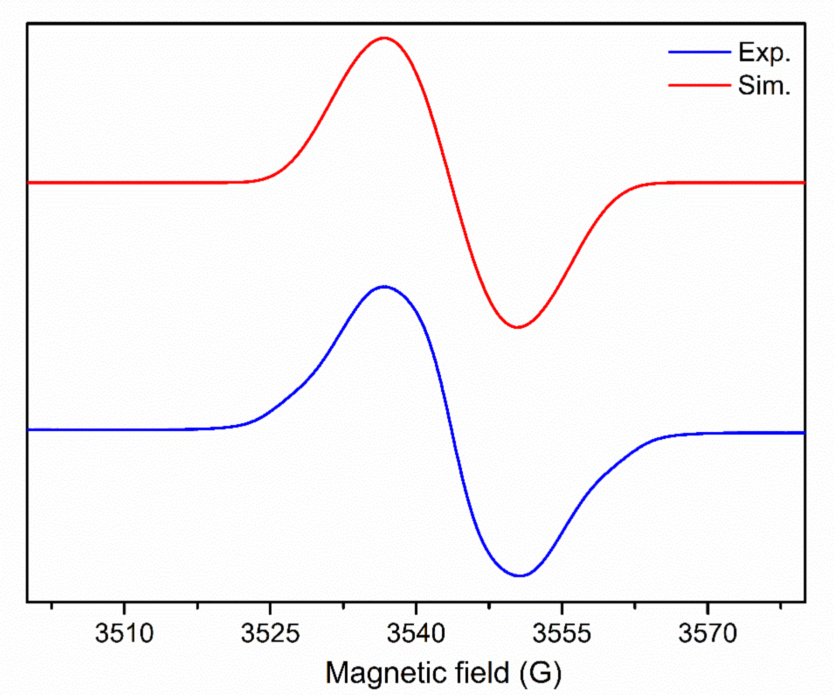

Supplementary Fig. 51. Experimental (blue) and simulated (red) EPR spectra of **3b^{III}** in THF solution at room temperature (c = 1.8 mM). Hyperfine couplings: g = 2.0029, A(2*^{14}N): 2.75 G, A(2*^{1}H): 2.35 G, A(2*^{1}H): 1.45 G.

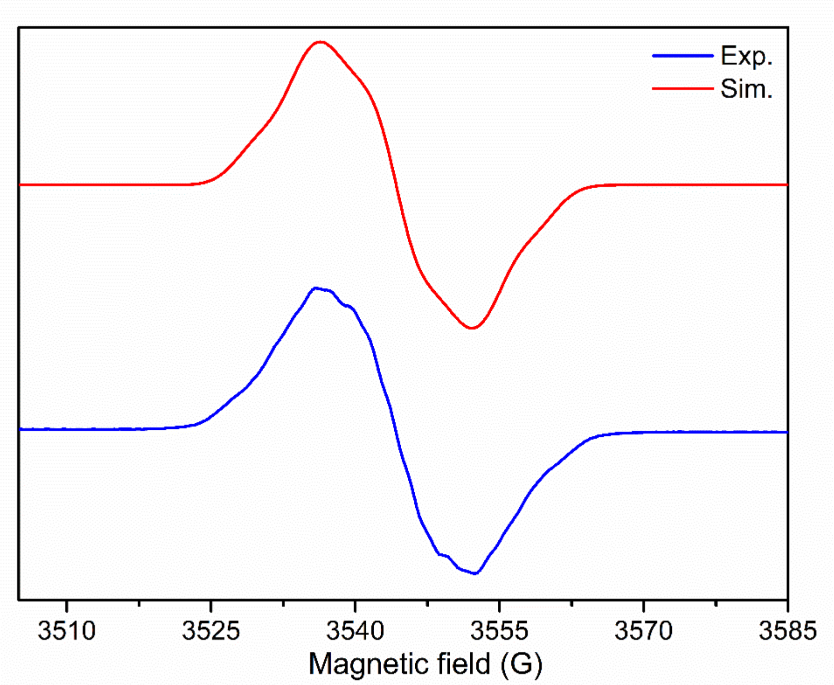

Supplementary Fig. 52. Experimental (blue) and simulated (red) EPR spectra of **3b^{IV}** in THF solution at room temperature (c = 1.8 mM). Hyperfine couplings: g = 2.0028, A(2*^{14}N): 2.35 G, A(2*^{1}H): 3.85 G.

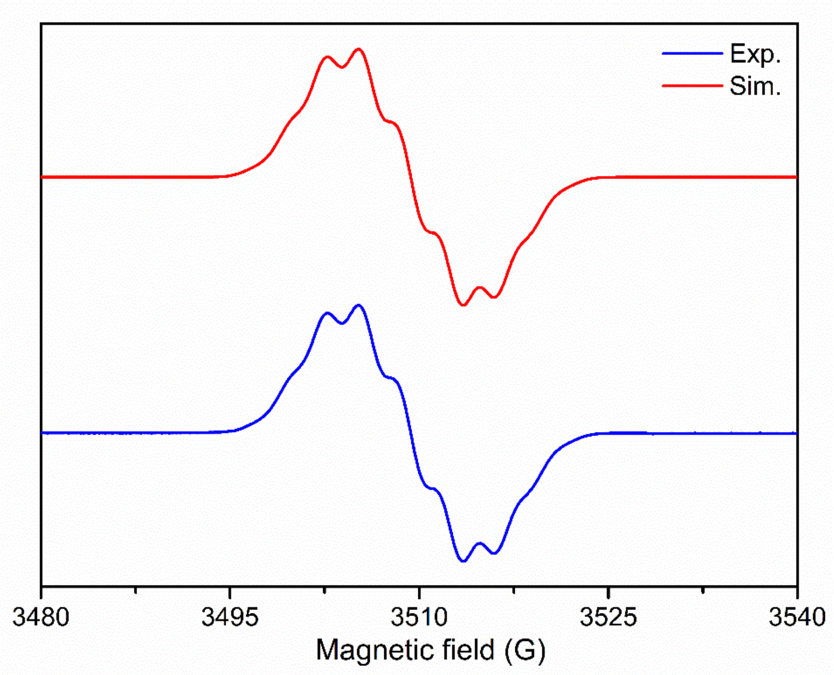

Supplementary Fig. 53. Experimental (blue) and simulated (red) EPR spectra of **3c^{I}** in THF solution at room temperature (*c* = 1.8 mM). Hyperfine couplings: *g* = 2.0028, *A*(2*^{14}N): 2.75 G; *A*(2*^{1}H): 3.02 G; *A*(2*^{1}H): 3.82 G.

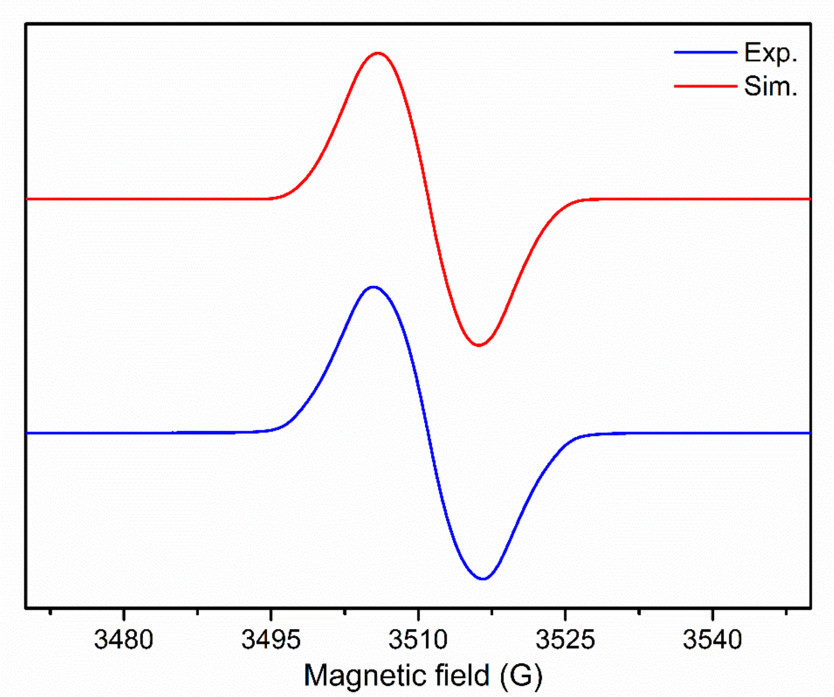

Supplementary Fig. 54. Experimental (blue) and simulated (red) EPR spectra of **4a** in THF solution at room temperature (*c* = 1.8 mM). Hyperfine couplings: *g* = 2.0029, *A*(2*^{14}N): 2.62 G; *A*(2*^{1}H): 3.85 G; *A*(2*^{1}H): 2.05 G.

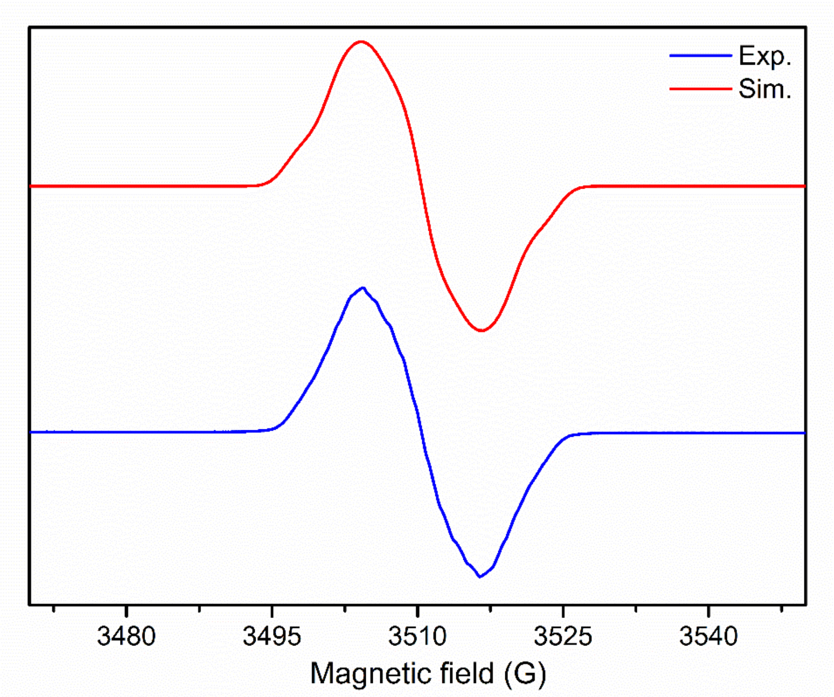

Supplementary Fig. 55. Experimental (blue) and simulated (red) EPR spectra of **4b** in THF solution at room temperature (c = 1.8 mM). Hyperfine couplings: g = 2.0030, $A(2{*}^{14}N)$: 3.15 G; $A(2{*}^{1}H)$: 4.35 G; $A(2{*}^{1}H)$: 1.65 G.

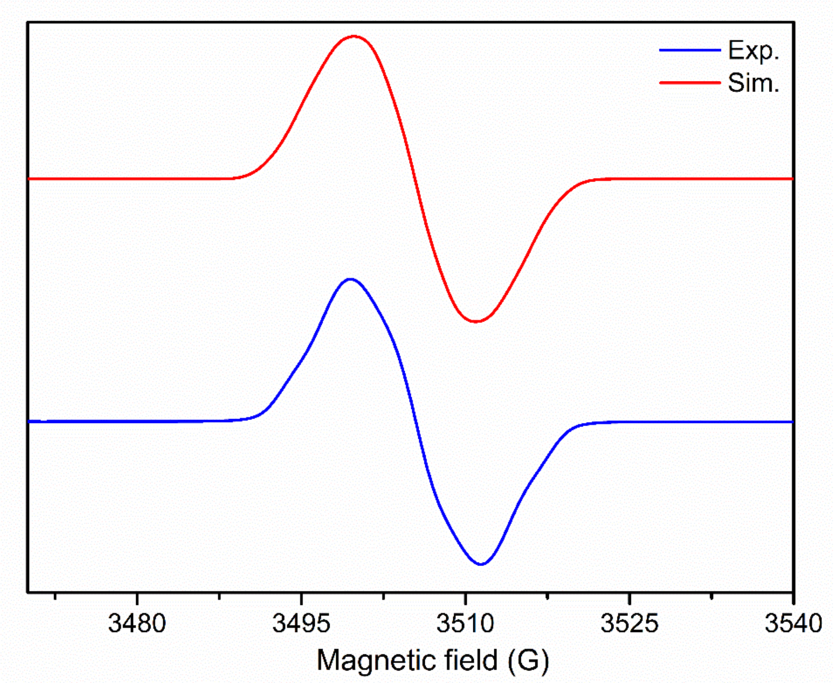

Supplementary Fig. 56. Experimental (blue) and simulated (red) EPR spectra of **4c** in THF solution at room temperature (c = 1.8 mM). Hyperfine couplings: g = 2.0030, $A(2{*}^{14}N)$: 3.40 G; $A(2{*}^{1}H)$: 2.95 G; $A(2{*}^{1}H)$: 1.90 G.

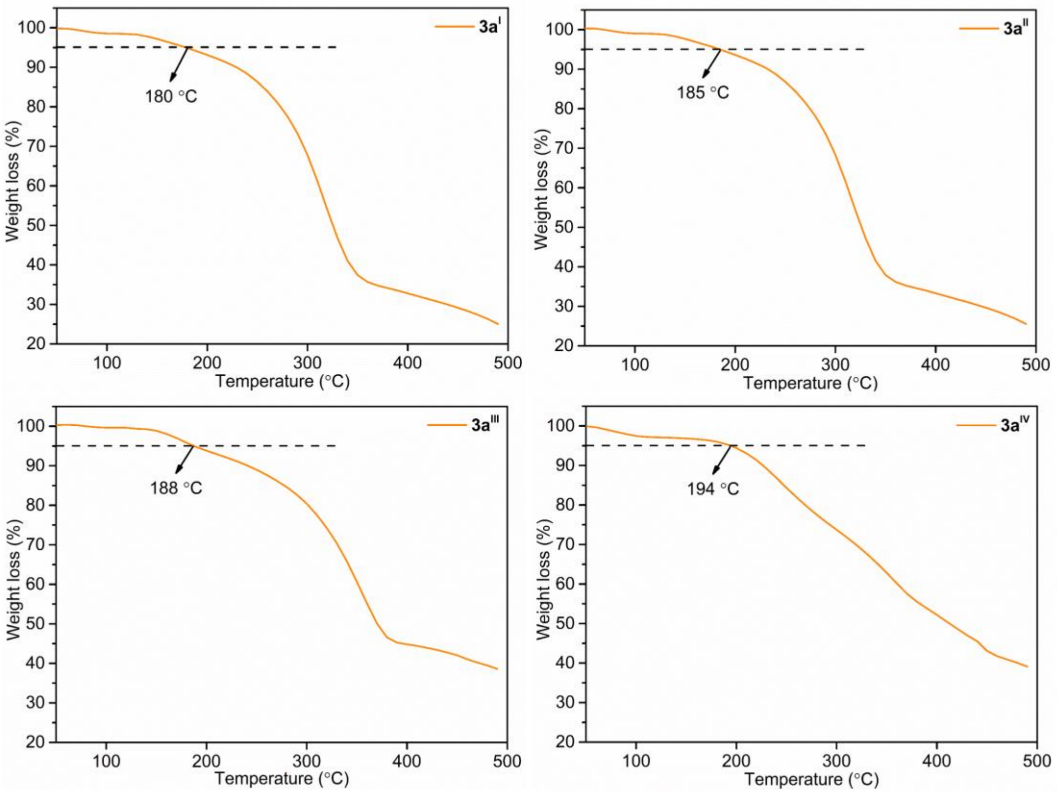

Supplementary Fig. 57. TGA curve of radicals **3a^{I}–3a^{IV}** under nitrogen flow.

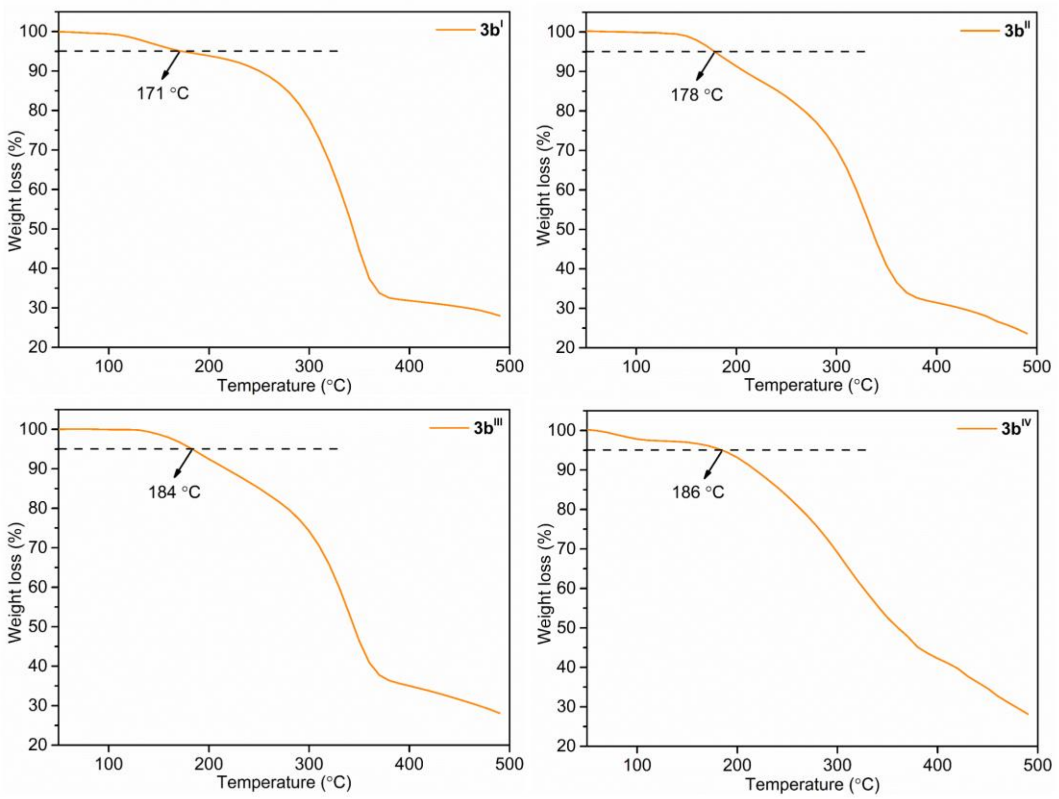

Supplementary Fig. 58. TGA curve of radicals **3b^{I}–3b^{IV}** under nitrogen flow.

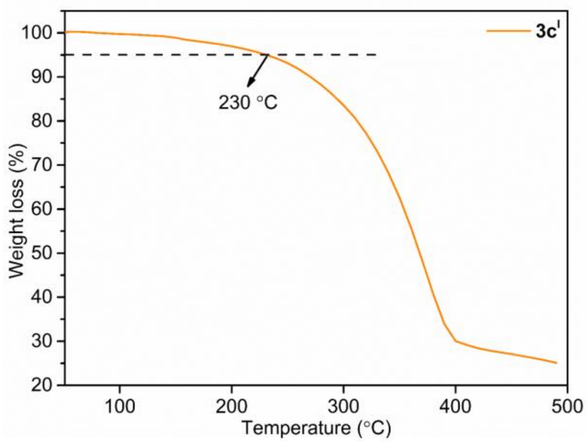

Supplementary Fig. 59. TGA curve of radical **3c**$^{\mathbf{I}}$ under nitrogen flow.

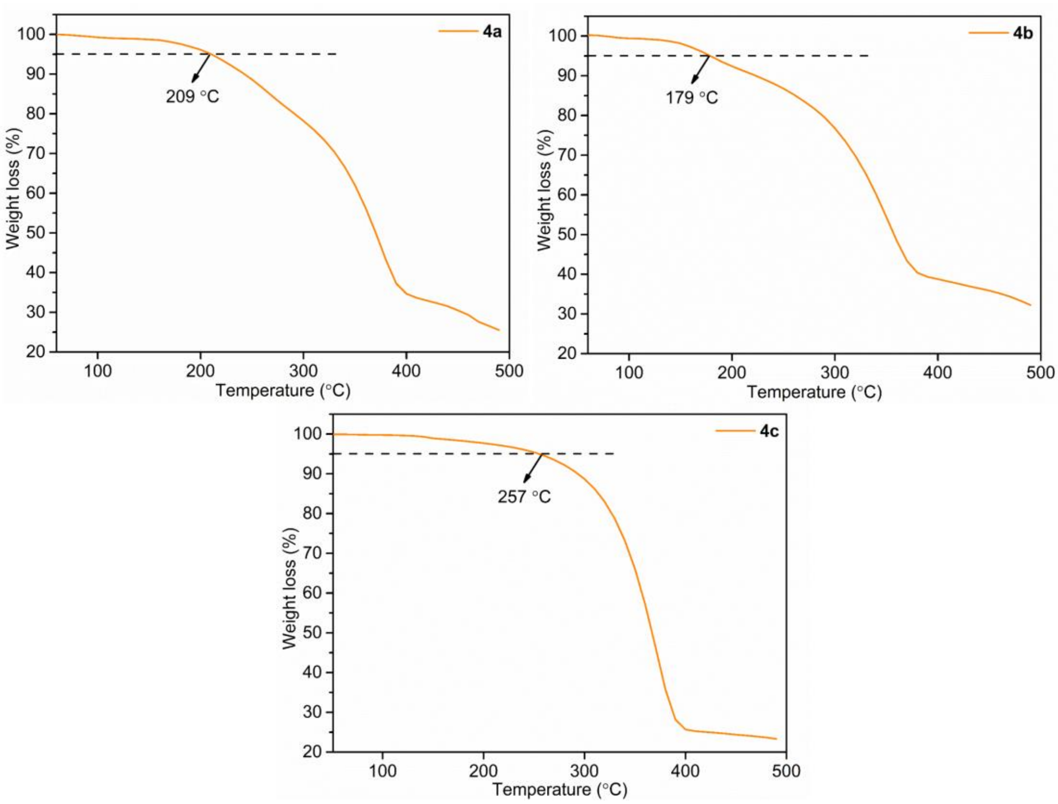

Supplementary Fig. 60. TGA curve of radicals **4a−c** under nitrogen flow.

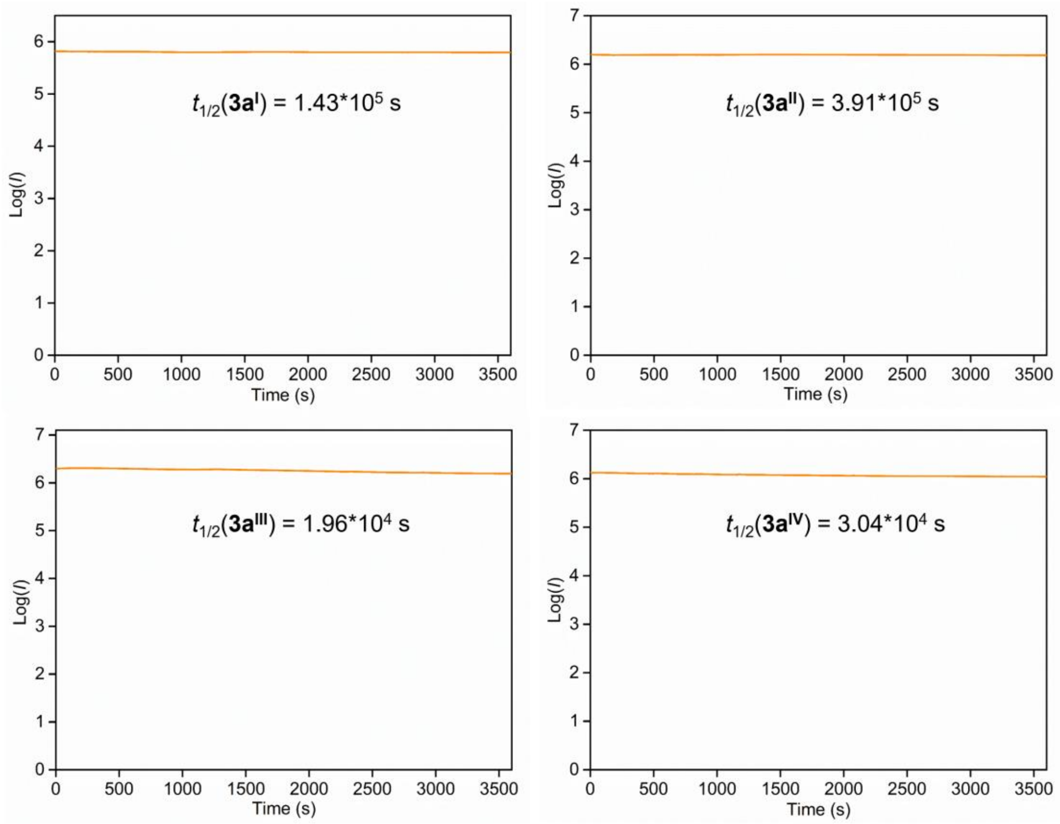

Supplementary Fig. 61. Plots showing the emission decay of radicals **3a**$^{\mathbf{I}}$–**3a**$^{\mathbf{IV}}$ in THF under continuous excitation with light at λ = 370 nm.

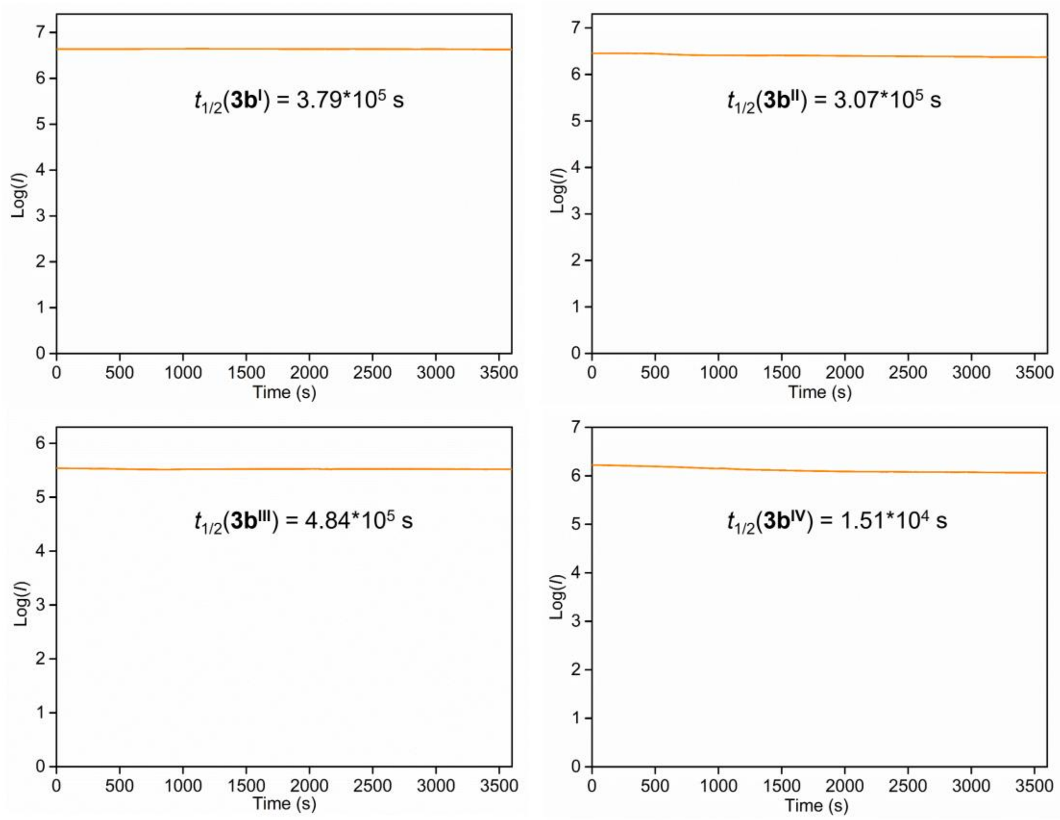

Supplementary Fig. 62. Plots showing the emission decay of radicals **3b**$^{\mathbf{I}}$–**3b**$^{\mathbf{IV}}$ in THF under continuous excitation with light at λ = 370 nm.

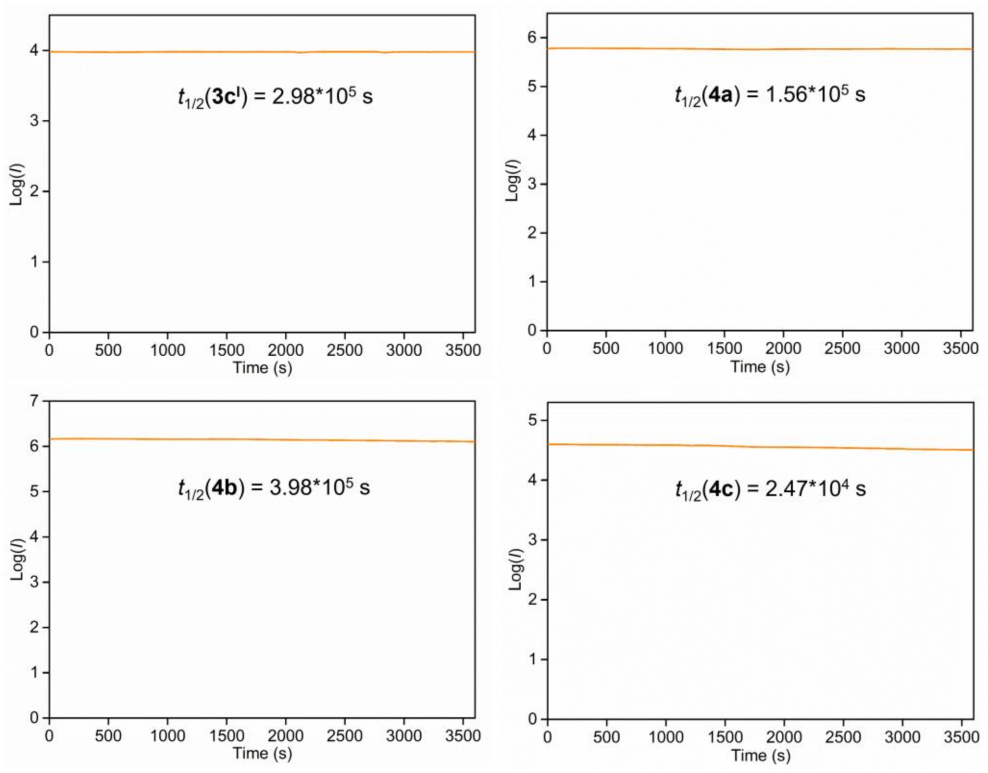

Supplementary Fig. 63. Plots showing the emission decay of radicals **3c**$^{\mathbf{I}}$ and **4a−c** in THF under continuous excitation with light at λ = 370 nm.

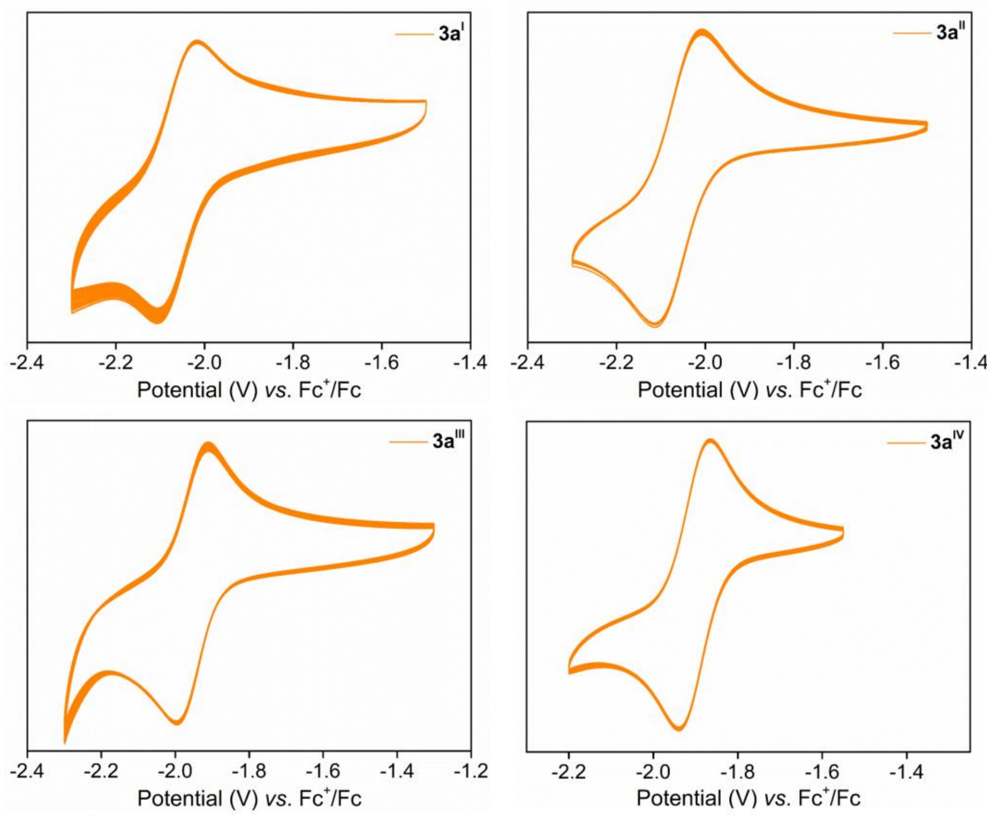

Supplementary Fig. 64. The multicycle CV curves of radicals **3a**$^{\mathbf{I}}$**–3a**$^{\mathbf{IV}}$ with scan rate of 100 mV s^{-1}.

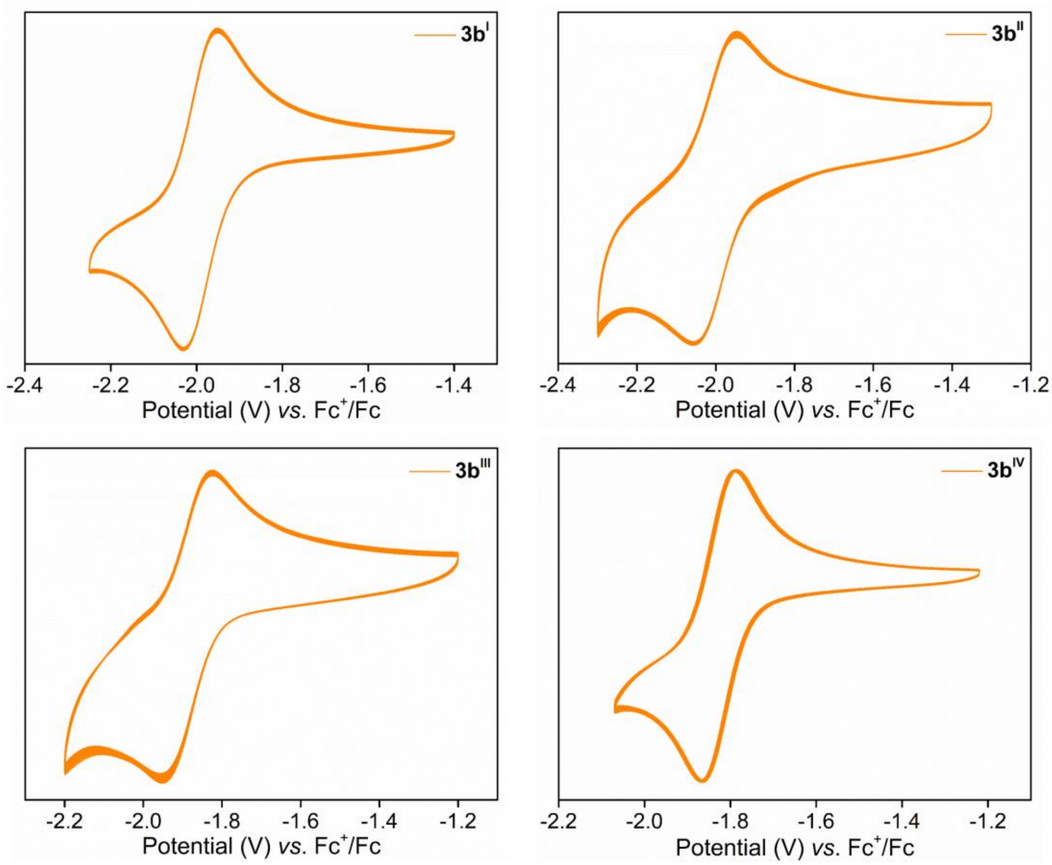

Supplementary Fig. 65. The multicycle CV curves of radicals **$3b^{I}$–$3b^{IV}$** with scan rate of 100 mV s^{-1}.

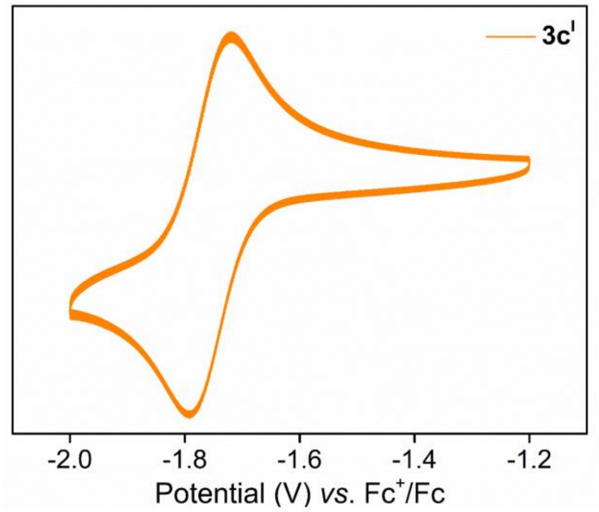

Supplementary Fig. 66. The multicycle CV curves of radicals **$3c^{I}$** with scan rate of 100 mV s^{-1}.

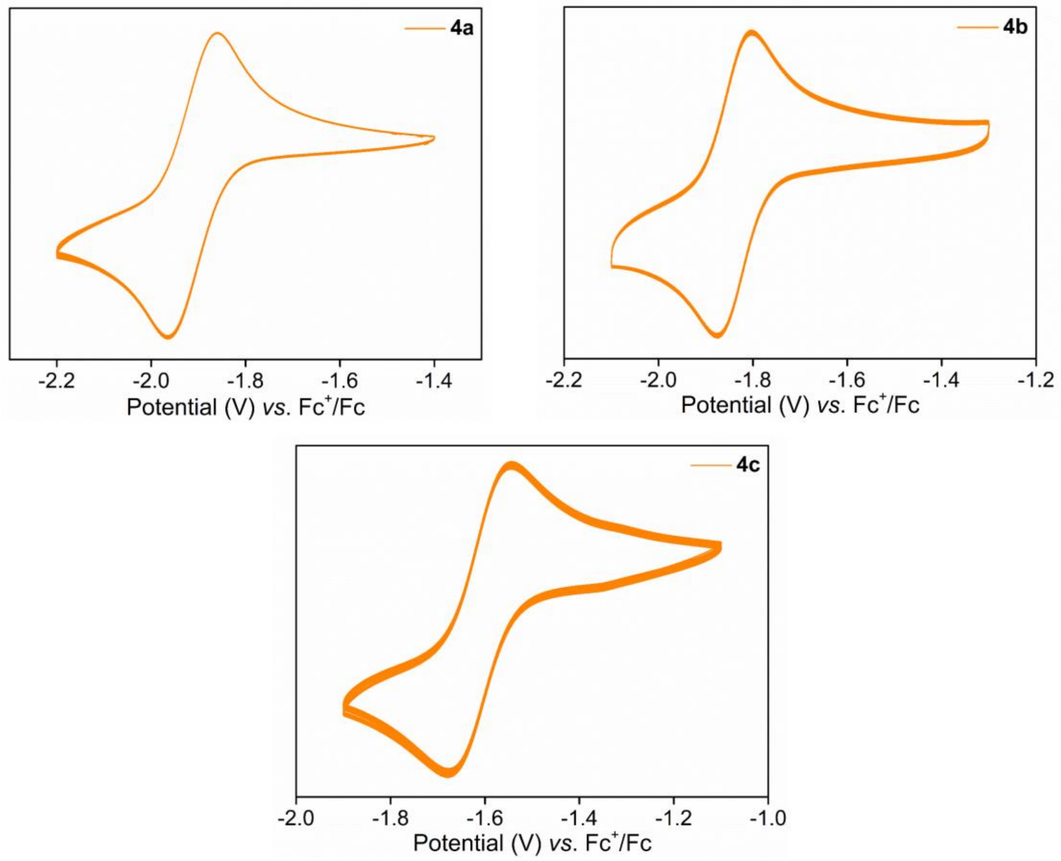

Supplementary Fig. 67. The multicycle CV curves of radicals **4a−c** with scan rate of 100 mV s^{-1}.

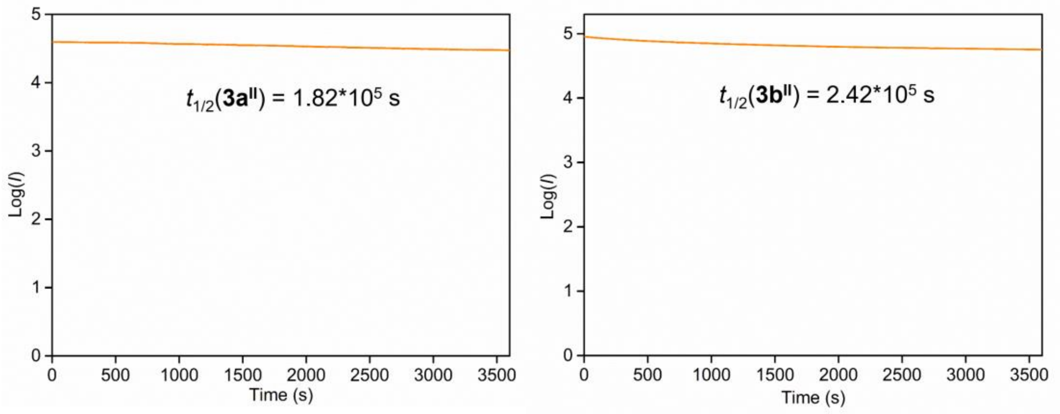

Supplementary Fig. 68. Plots showing the emission decay of a polymethyl methacrylate (PMMA) film containing radicals **3a^{II}** or **3b^{II} (**5% wt**)** under continuous excitation with light at λ = 370 nm.

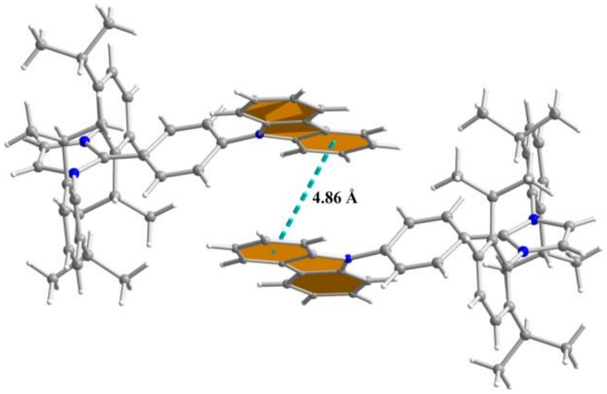

Supplementary Fig. 69. π-stacking interactions in carbazolylpheyl-based radical **4b**.

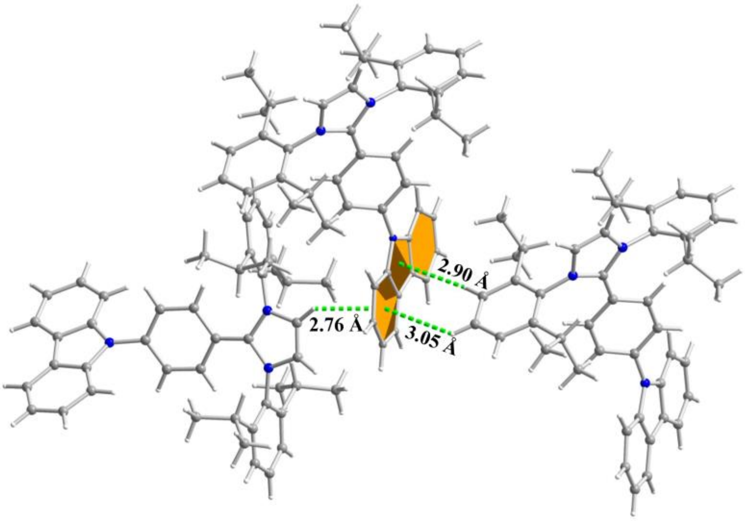

Supplementary Fig. 70. C-H/π interactions in carbazolylpheyl-based radical **4b**.

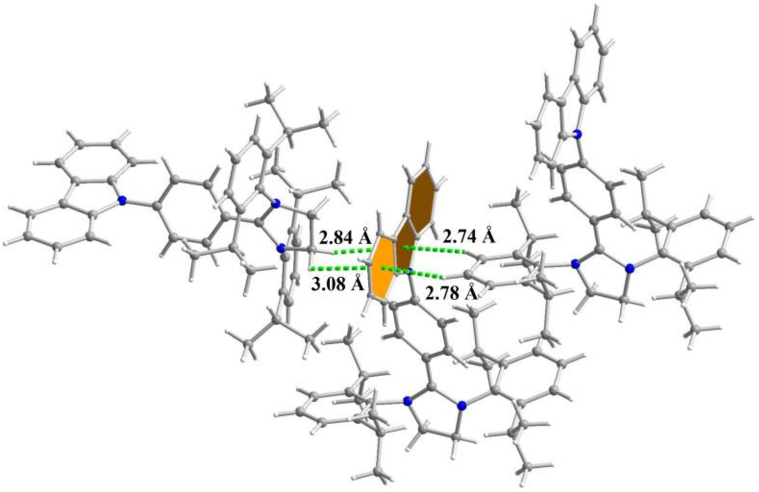

Supplementary Fig. 71. C-H/π interactions in carbazolylpheyl-based radical **4c**.

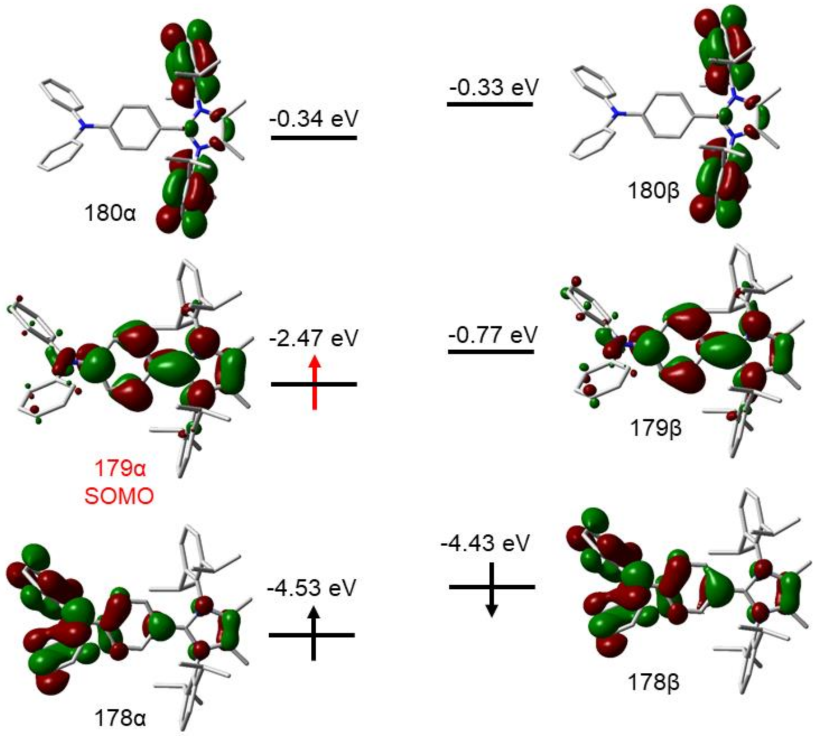

Supplementary Fig. 72. Energies and wave functions for the frontier molecular orbitals of **3a^{I}**, as calculated at the UB3LYP/6-31G(d) level of theory.

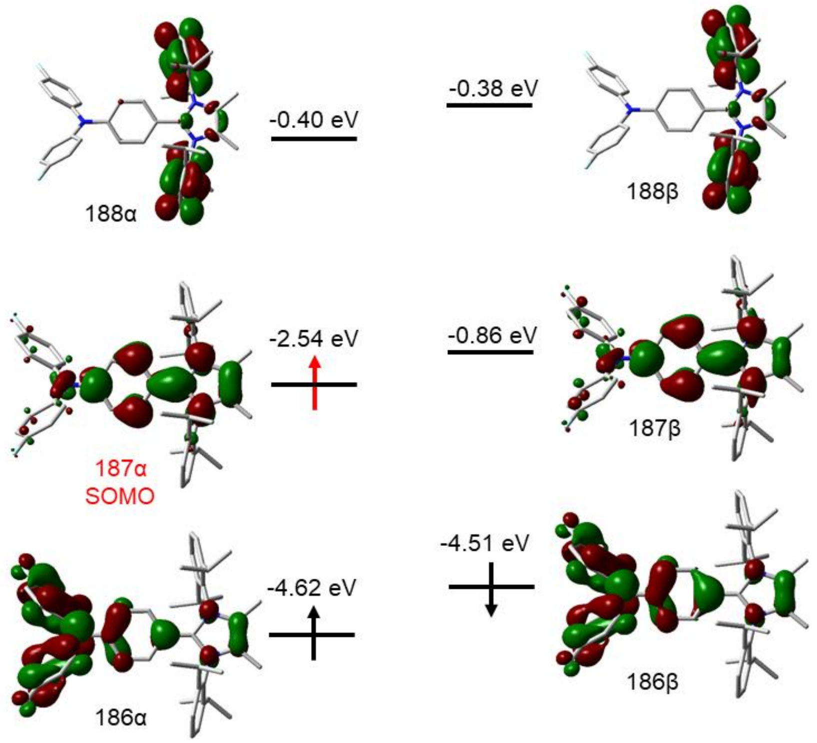

Supplementary Fig. 73. Energies and wave functions for the frontier molecular orbitals of **3a^{II}**, as calculated at the UB3LYP/6-31G(d) level of theory.

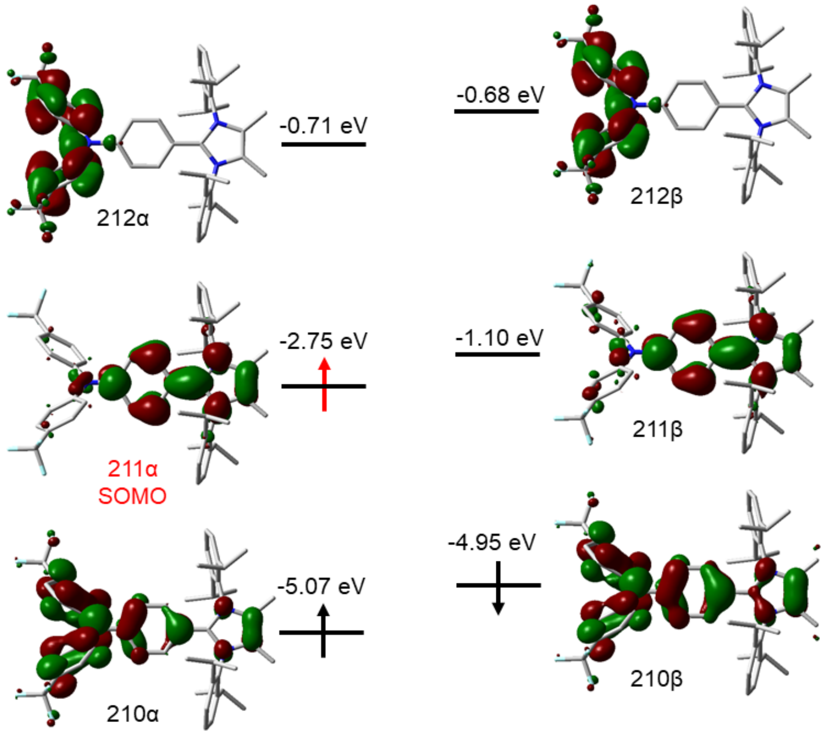

Supplementary Fig. 74. Energies and wave functions for the frontier molecular orbitals of **3a**$^{\mathbf{III}}$, as calculated at the UB3LYP/6-31G(d) level of theory.

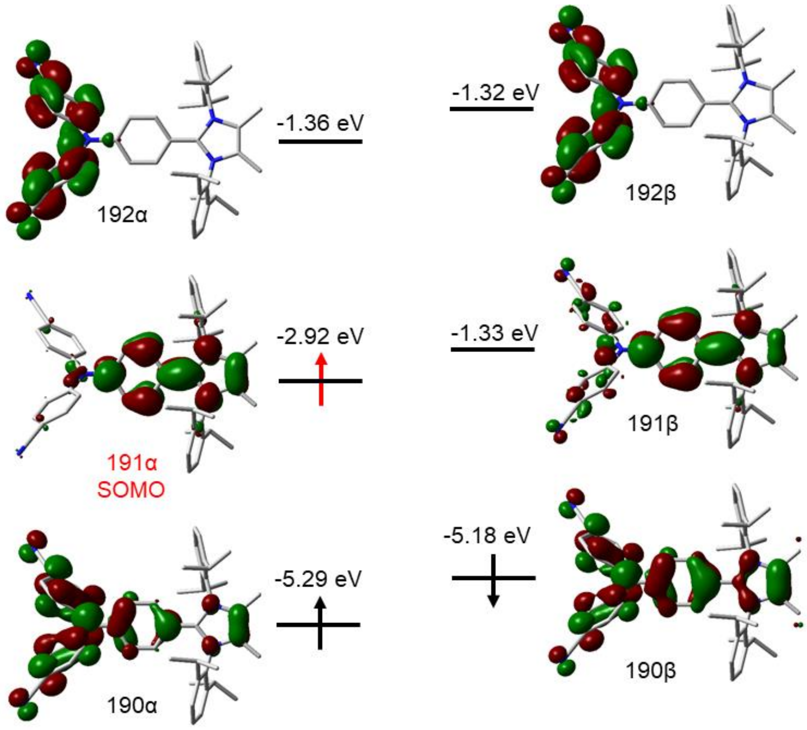

Supplementary Fig. 75. Energies and wave functions for the frontier molecular orbitals of **3a**$^{\mathbf{IV}}$, as calculated at the UB3LYP/6-31G(d) level of theory.

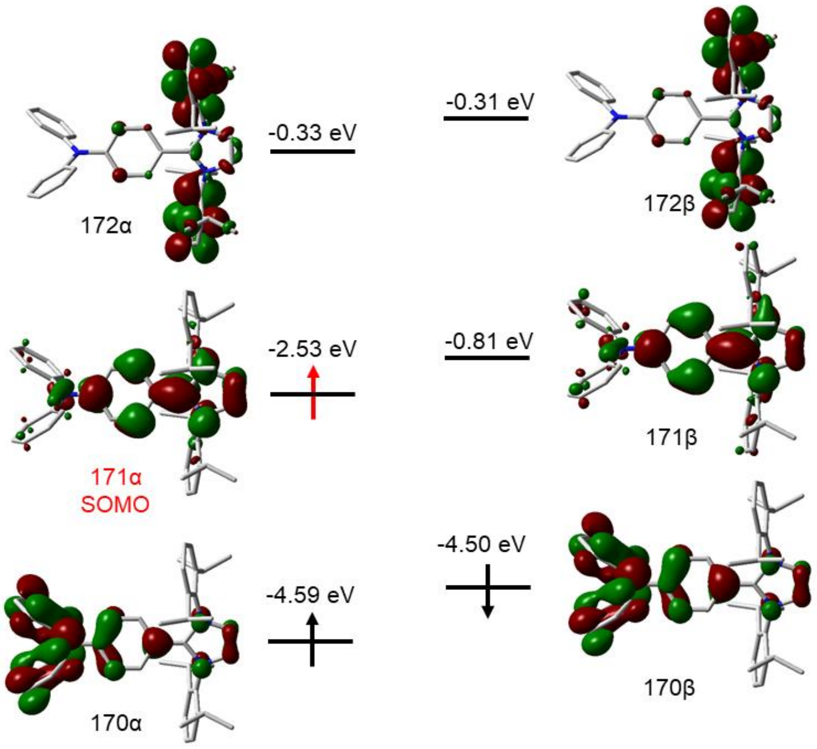

Supplementary Fig. 76. Energies and wave functions for the frontier molecular orbitals of **3b^I^**, as calculated at the UB3LYP/6-31G(d) level of theory.

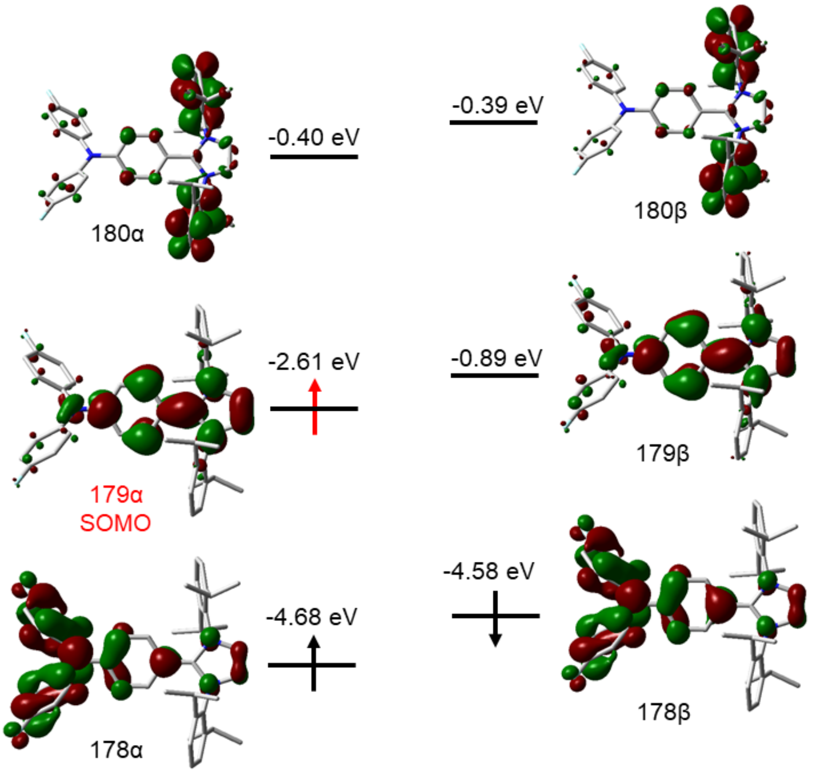

Supplementary Fig. 77. Energies and wave functions for the frontier molecular orbitals of **3b^{II}**, as calculated at the UB3LYP/6-31G(d) level of theory.

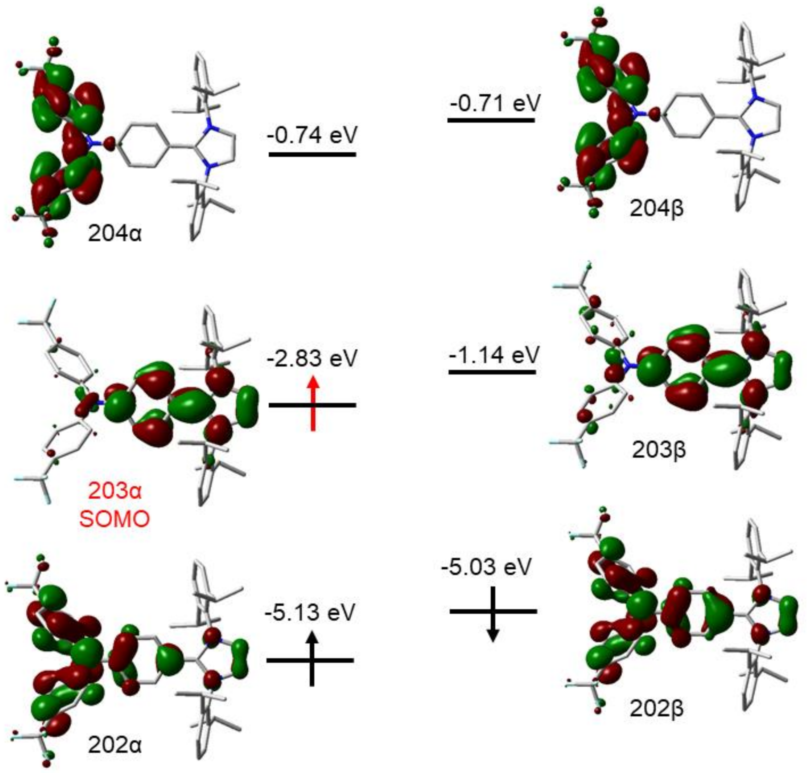

Supplementary Fig. 78. Energies and wave functions for the frontier molecular orbitals of **3b^{III}**, as calculated at the UB3LYP/6-31G(d) level of theory.

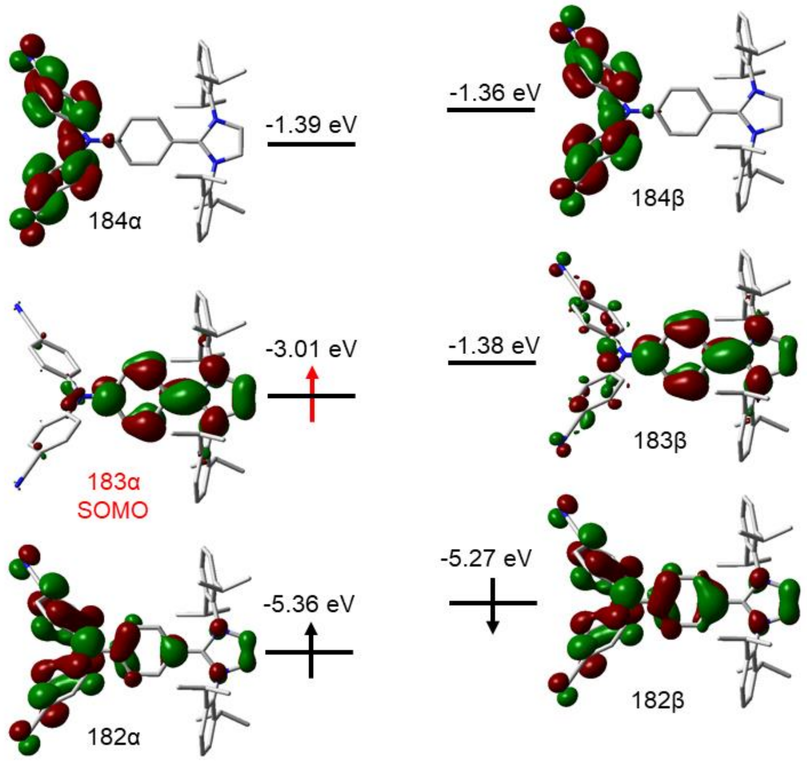

Supplementary Fig. 79. Energies and wave functions for the frontier molecular orbitals of **3b^{IV}**, as calculated at the UB3LYP/6-31G(d) level of theory.

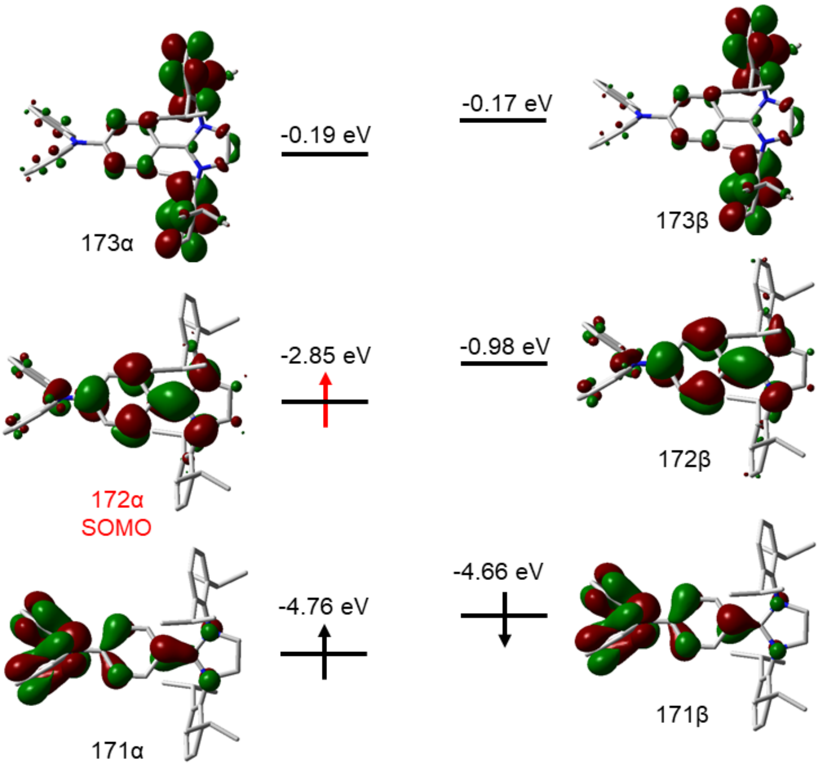

Supplementary Fig. 80. Energies and wave functions for the frontier molecular orbitals of **3c**$^{\mathbf{I}}$, as calculated at the UB3LYP/6-31G(d) level of theory.

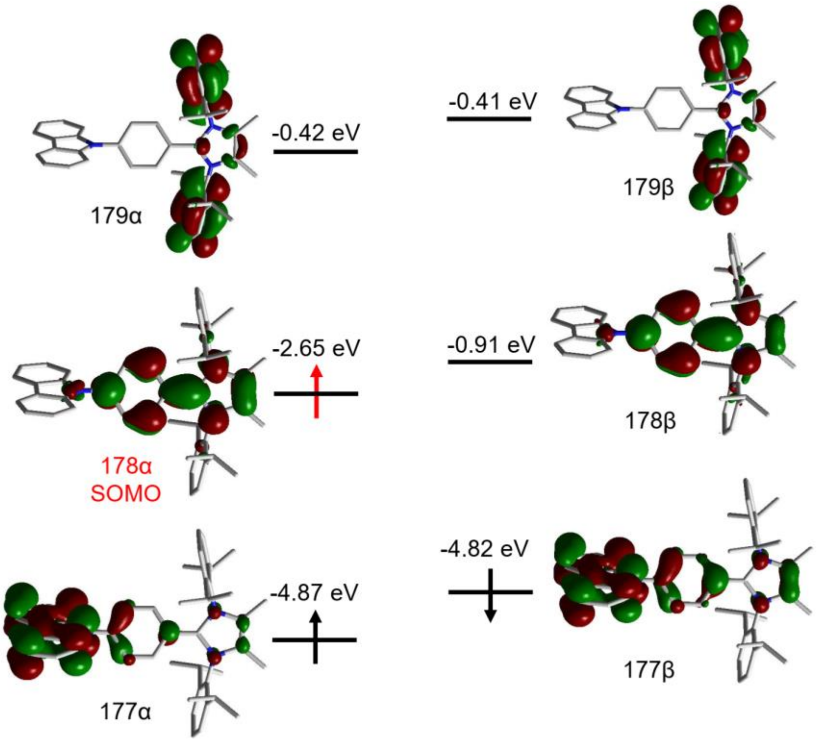

Supplementary Fig. 81. Energies and wave functions for the frontier molecular orbitals of **4a**, as calculated at the UB3LYP/6-31G(d) level of theory.

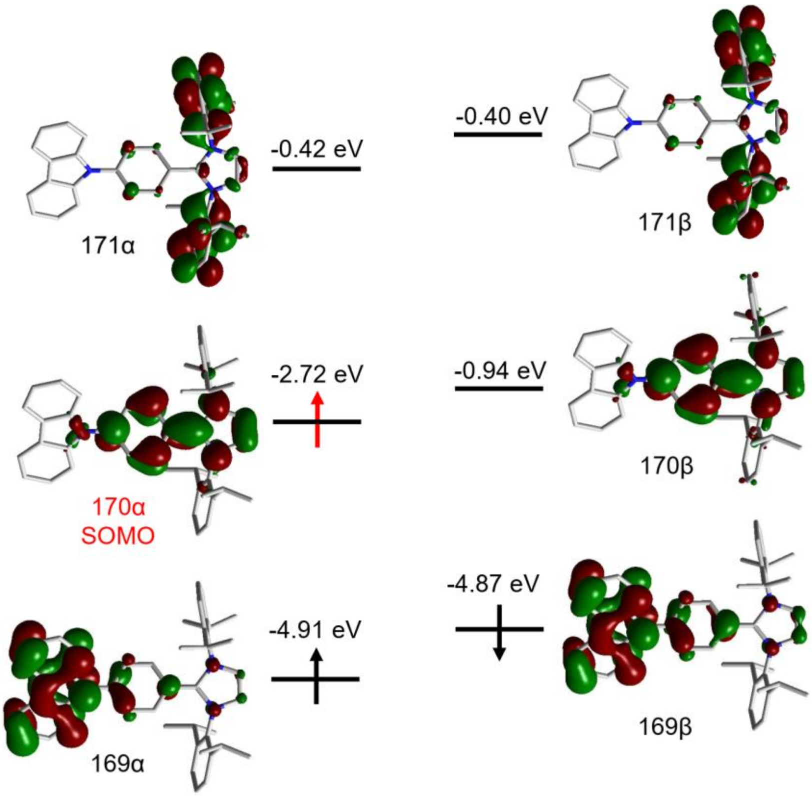

Supplementary Fig. 82. Energies and wave functions for the frontier molecular orbitals of **4b**, as calculated at the UB3LYP/6-31G(d) level of theory.

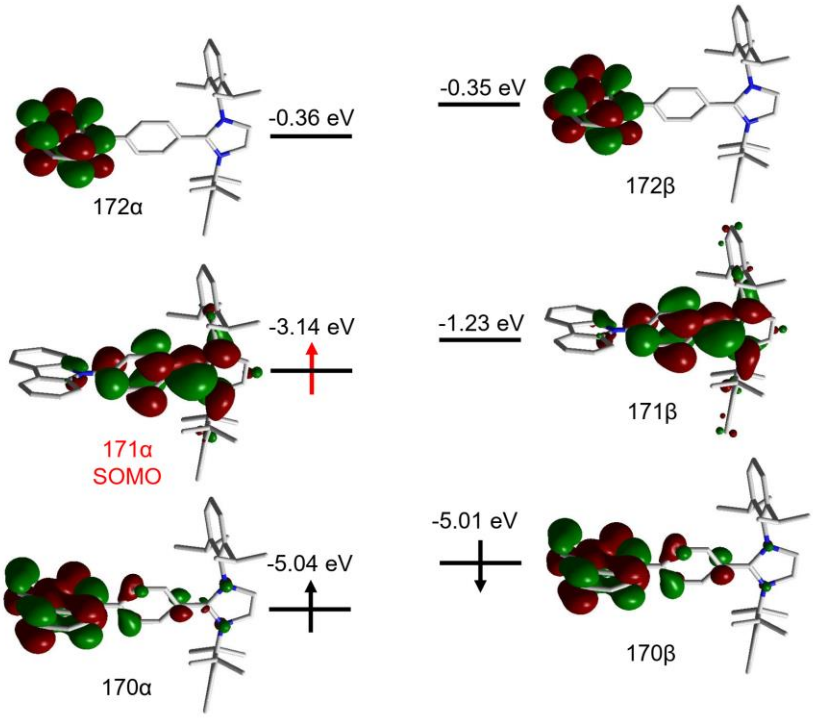

Supplementary Fig. 83. Energies and wave functions for the frontier molecular orbitals of **4c**, as calculated at the UB3LYP/6-31G(d) level of theory.

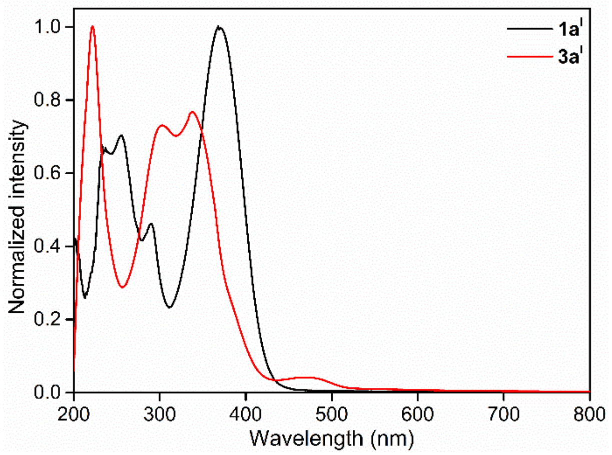

Supplementary Fig. 84. UV-vis spectra of **1a^{I}** and **3a^{I}** in THF solvent at room temperature (*c* = 10^{-4} M).

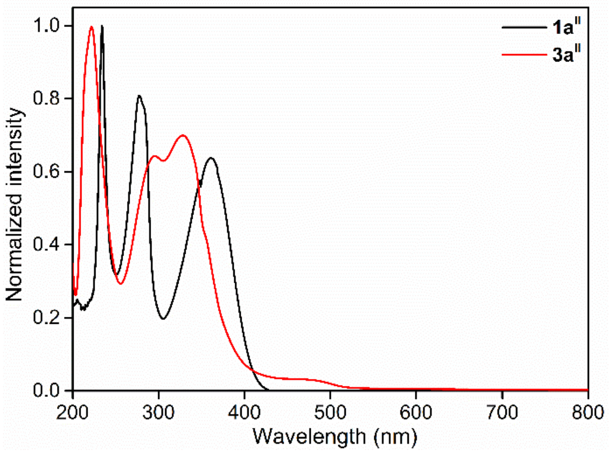

Supplementary Fig. 85. UV-vis spectra of **1a^{II}** and **3a^{II}** in THF solvent at room temperature (*c* = 10^{-4} M).

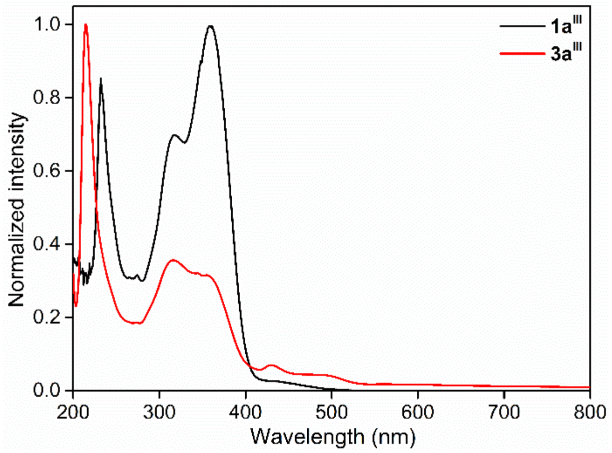

Supplementary Fig. 86. UV-vis spectra of **1a^{III}** and **3a^{III}** in THF solvent at room temperature (*c* = 10^{-4} M).

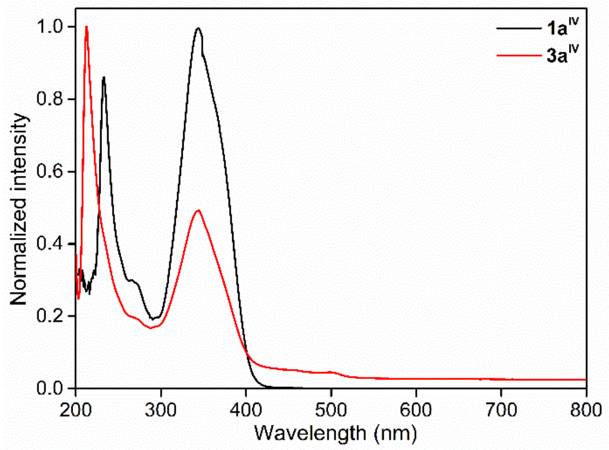

Supplementary Fig. 87. UV-vis spectra of **1a**$^{\mathbf{IV}}$ and **3a**$^{\mathbf{IV}}$ in THF solvent at room temperature (c = 10^{-4} M).

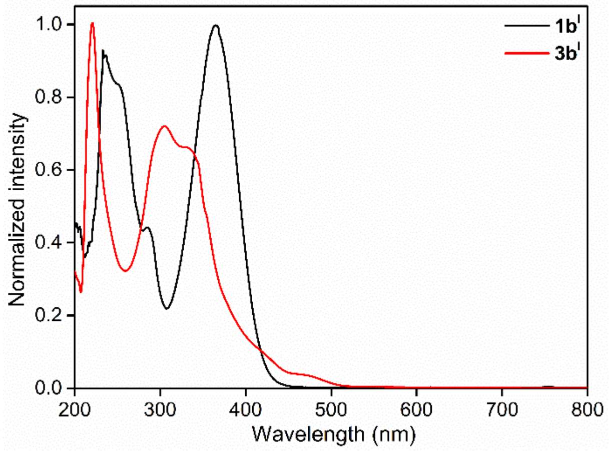

Supplementary Fig. 88. UV-vis spectra of **1b**$^{\mathbf{I}}$ and **3b**$^{\mathbf{I}}$ in THF solvent at room temperature (c = 10^{-4} M).

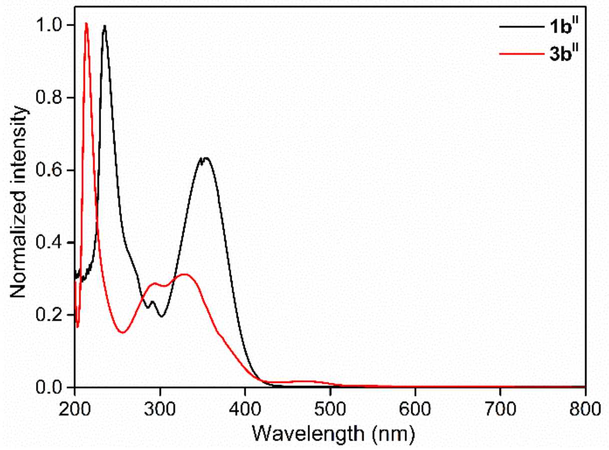

Supplementary Fig. 89. UV-vis spectra of **1b**$^{\mathbf{II}}$ and **3b**$^{\mathbf{II}}$ in THF solvent at room temperature (c = 10^{-4} M).

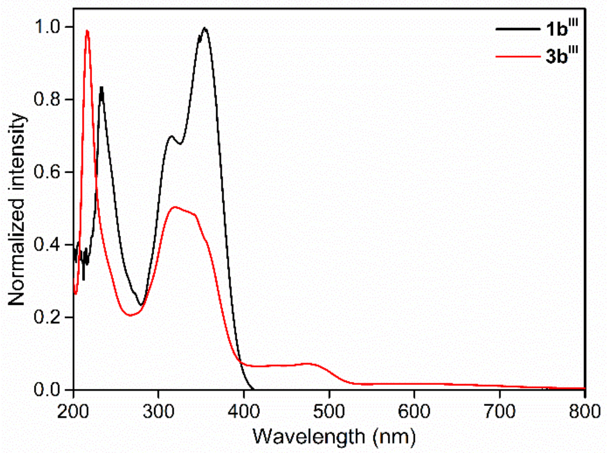

Supplementary Fig. 90. UV-vis spectra of **1b^{III}** and **3b^{III}** in THF solvent at room temperature (c = 10^{-4} M).

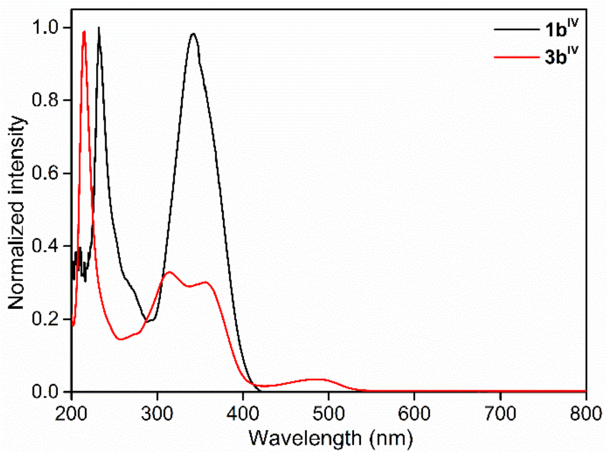

Supplementary Fig. 91. UV-vis spectra of **1b^{IV}** and **3b^{IV}** in THF solvent at room temperature (c = 10^{-4} M).

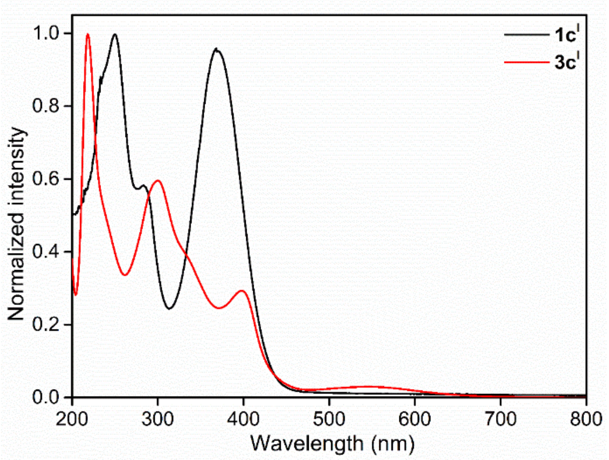

Supplementary Fig. 92. UV-vis spectra of **1c^{I}** and **3c^{I}** in THF solvent at room temperature (c = 10^{-4} M).

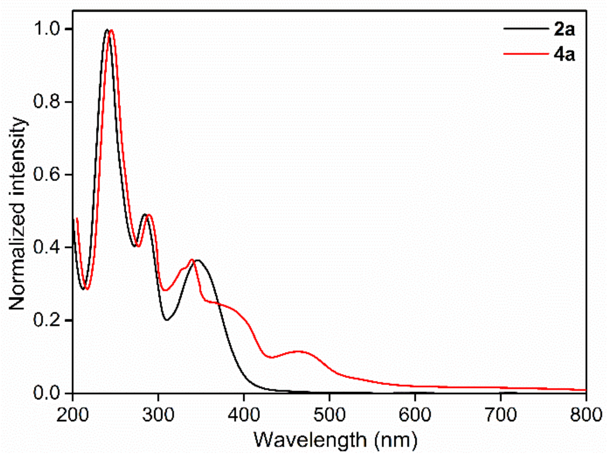

Supplementary Fig. 93. UV-vis spectra of **2a** and **4a** in THF solvent at room temperature ($c = 10^{-4}$ M).

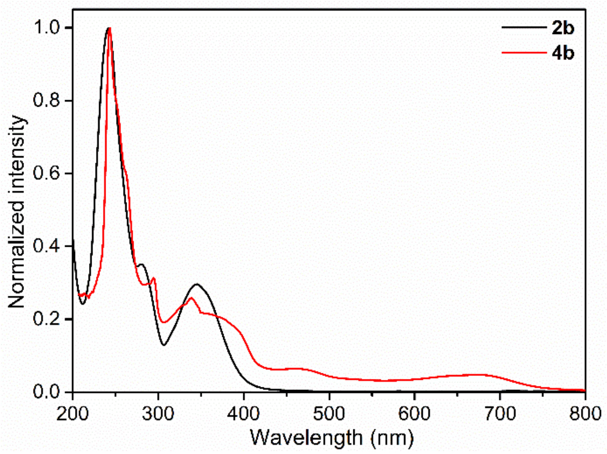

Supplementary Fig. 94. UV-vis spectra of **2b** and **4b** in THF solvent at room temperature ($c = 10^{-4}$ M).

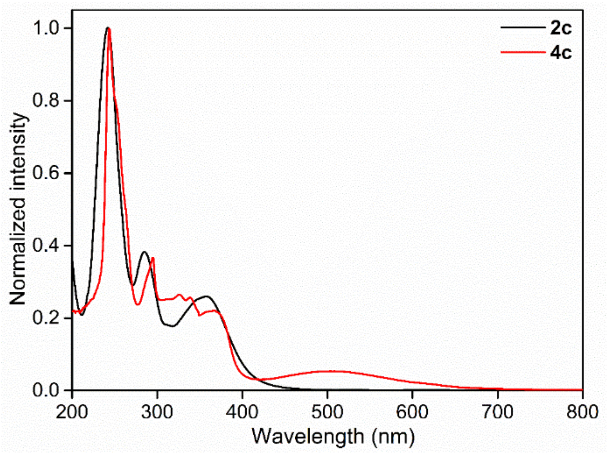

Supplementary Fig. 95. UV-vis spectra of **2c** and **4c** in THF solvent at room temperature ($c = 10^{-4}$ M).

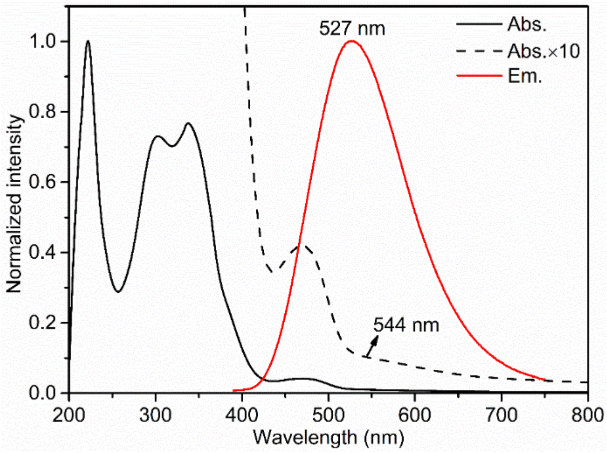

Supplementary Fig. 96. Absorption and emission spectra of **3a^{I}** in THF at room temperature. Enlarged portion of absorption spectra (10 fold) are shown.

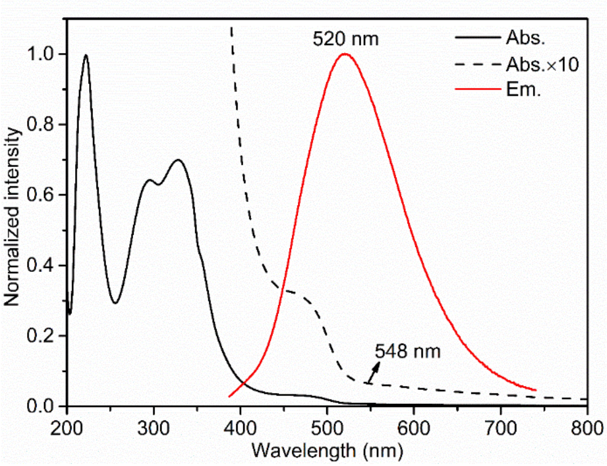

Supplementary Fig. 97. Absorption and emission spectra of **3a^{II}** in THF at room temperature. Enlarged portion of absorption spectra (10 fold) are shown.

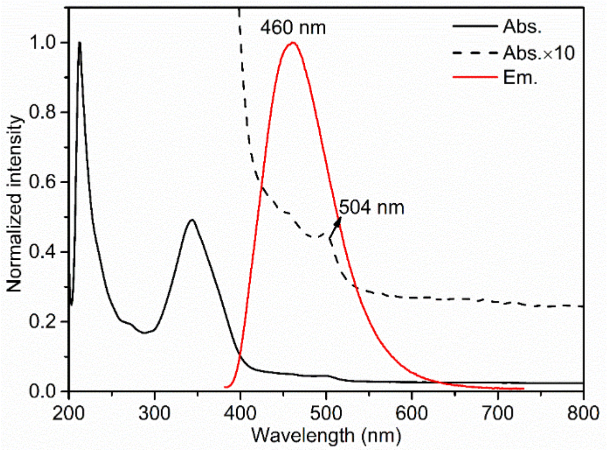

Supplementary Fig. 98. Absorption and emission spectra of **3a^{IV}** in THF at room temperature. Enlarged portion of absorption spectra (10 fold) are shown.

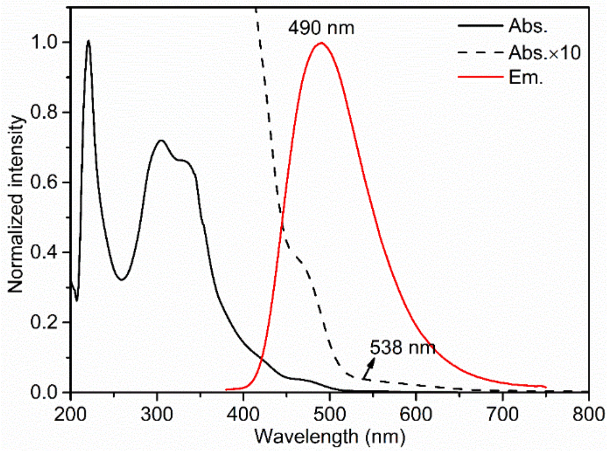

Supplementary Fig. 99. Absorption and emission spectra of **3b**$^{\mathbf{I}}$ in THF at room temperature. Enlarged portion of absorption spectra (10 fold) are shown.

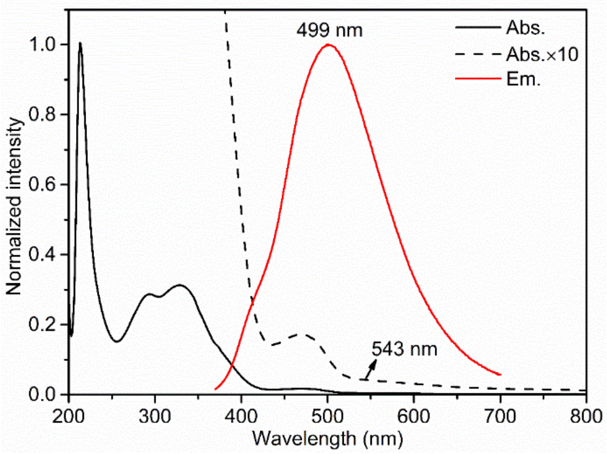

Supplementary Fig. 100. Absorption and emission spectra of **3b**$^{\mathbf{II}}$ in THF at room temperature. Enlarged portion of absorption spectra (10 fold) are shown.

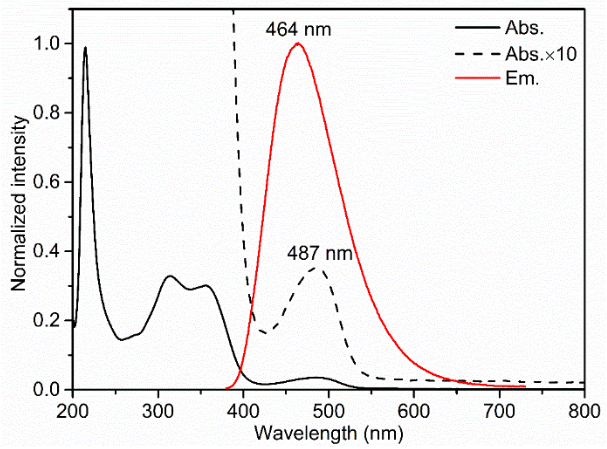

Supplementary Fig. 101. Absorption and emission spectra of **3b**$^{\mathbf{IV}}$ in THF at room temperature. Enlarged portion of absorption spectra (10 fold) are shown.

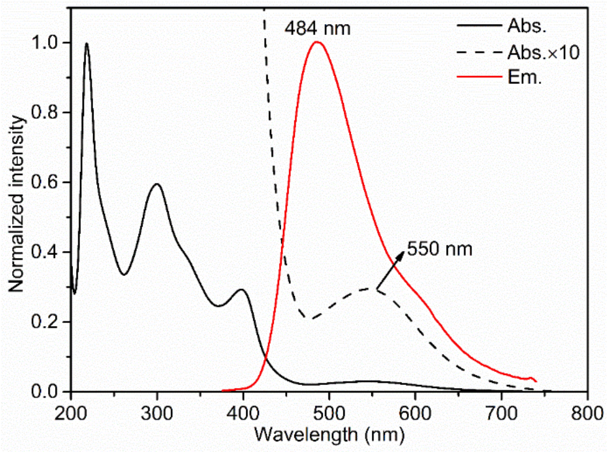

Supplementary Fig. 102. Absorption and emission spectra of **3c^{I}** in THF at room temperature. Enlarged portion of absorption spectra (10 fold) are shown.

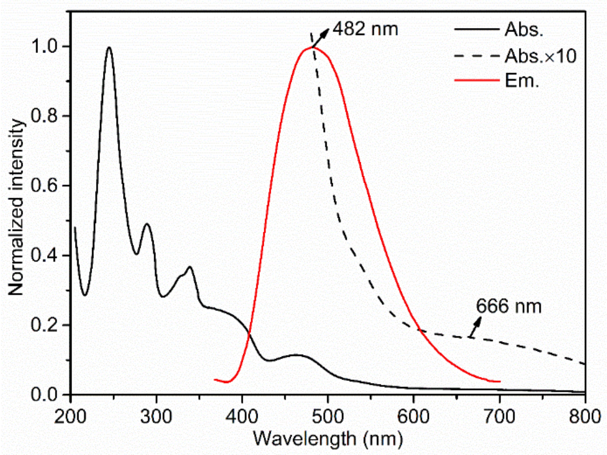

Supplementary Fig. 103. Absorption and emission spectra of **4a** in THF at room temperature. Enlarged portion of absorption spectra (10 fold) are shown.

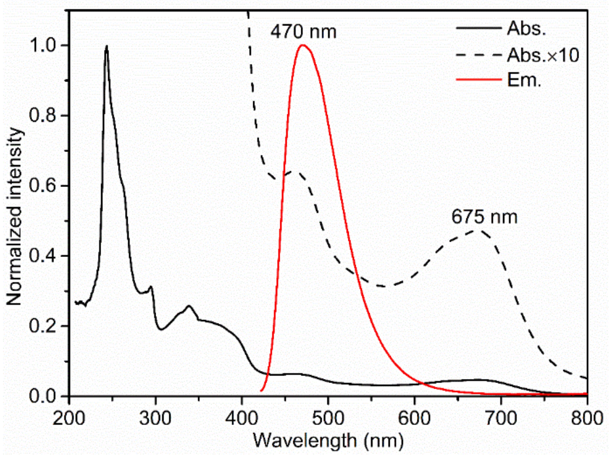

Supplementary Fig. 104. Absorption and emission spectra of **4b** in THF at room temperature. Enlarged portion of absorption spectra (10 fold) are shown.

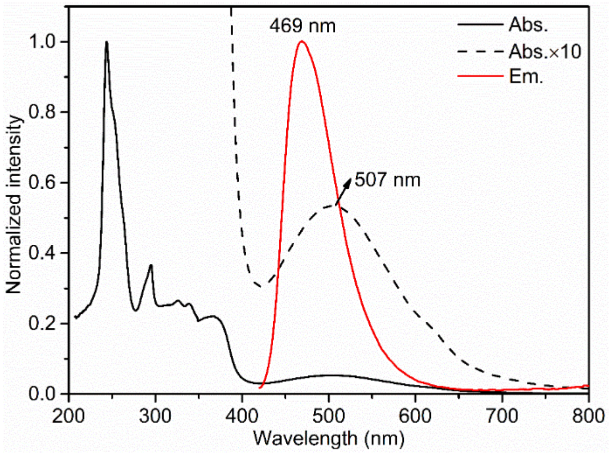

Supplementary Fig. 105. Absorption and emission spectra of **4c** in THF at room temperature. Enlarged portion of absorption spectra (10 fold) are shown.

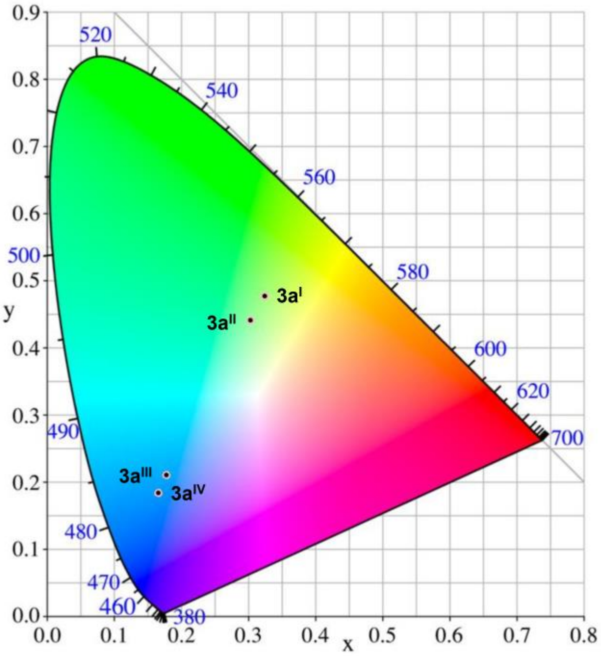

Supplementary Fig. 106. CIE chromaticity coordinates of radicals **3a^{I}–3a^{IV}**, where X is the chromaticity coordinate that represents the proportion of red primary and Y is the chromaticity coordinate that represents the proportion of green primary.

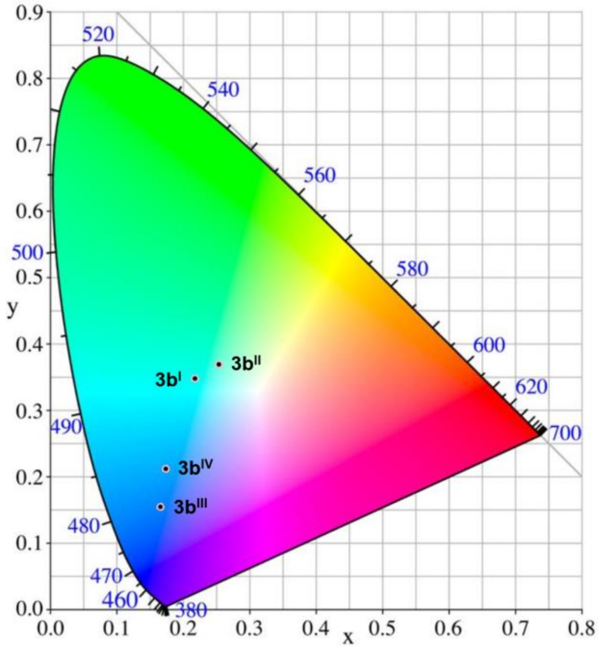

Supplementary Fig. 107. CIE chromaticity coordinates of radicals $\mathbf{3b^{I}}$–$\mathbf{3b^{IV}}$, where X is the chromaticity coordinate that represents the proportion of red primary and Y is the chromaticity coordinate that represents the proportion of green primary.

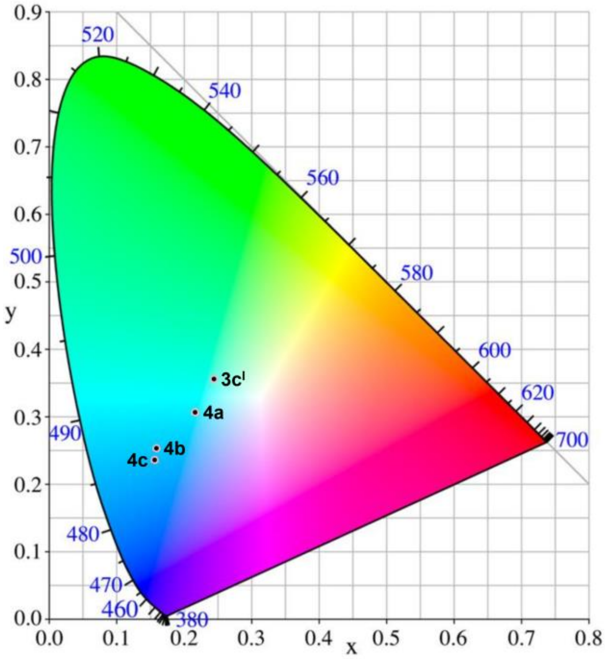

Supplementary Fig. 108. CIE chromaticity coordinates of radicals $\mathbf{3c^{I}}$ and **4a–c**, where X is the chromaticity coordinate that represents the proportion of red primary and Y is the chromaticity coordinate that represents the proportion of green primary.

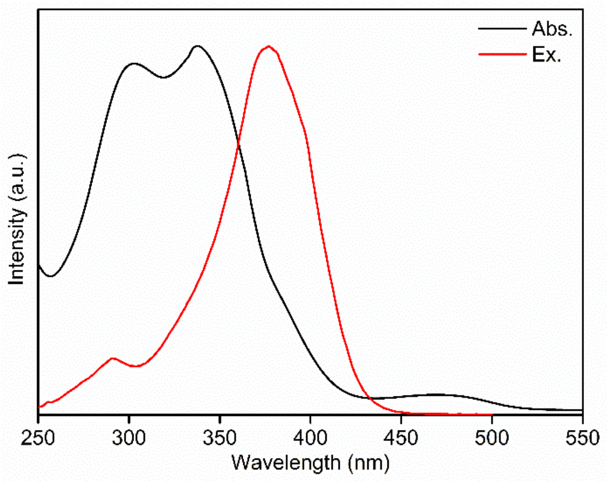

Supplementary Fig. 109. The absorption and excitation spectra of **3a**$^{\mathbf{I}}$ (λ_{em} = 529 nm) in THF at room temperature.

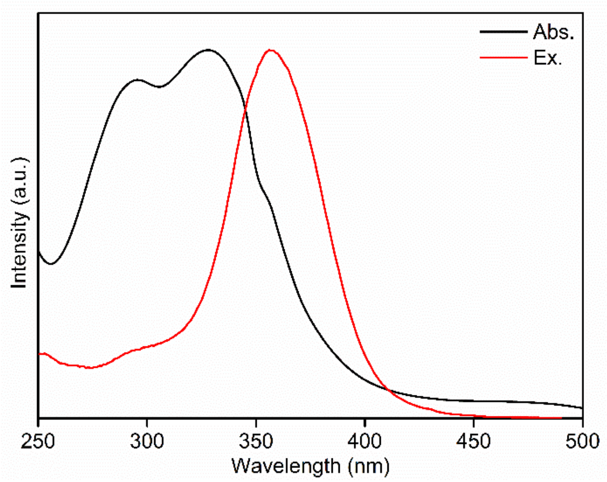

Supplementary Fig. 110. The absorption and excitation spectra of **3a**$^{\mathbf{II}}$ (λ_{em} = 520 nm) in THF at room temperature.

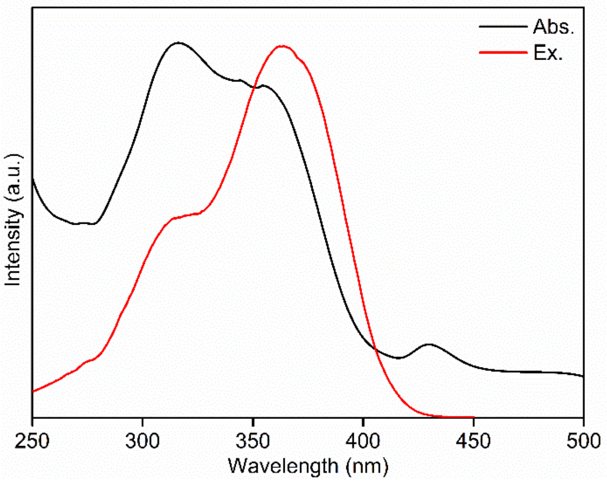

Supplementary Fig. 111. The absorption and excitation spectra of **3a**$^{\mathbf{III}}$ (λ_{em} = 463 nm) in THF at room temperature.

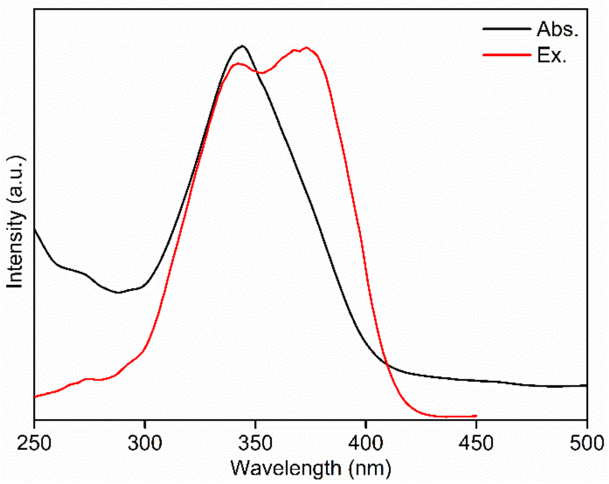

Supplementary Fig. 112. The absorption and excitation spectra of **3a^{IV}** (λ_{em} = 460 nm) in THF at room temperature.

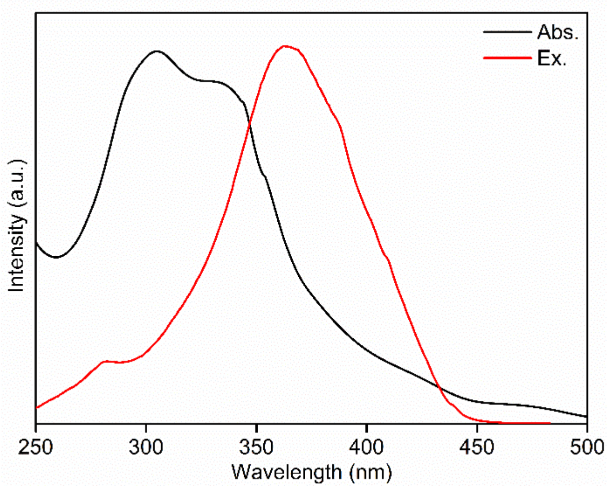

Supplementary Fig. 113. The absorption and excitation spectra of **3b^{I}** (λ_{em} = 490 nm) in THF at room temperature.

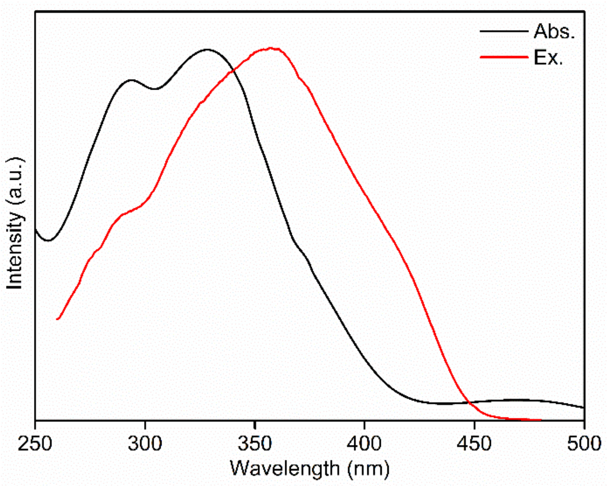

Supplementary Fig. 114. The absorption and excitation spectra of **3b^{II}** (λ_{em} = 499 nm) in THF at room temperature.

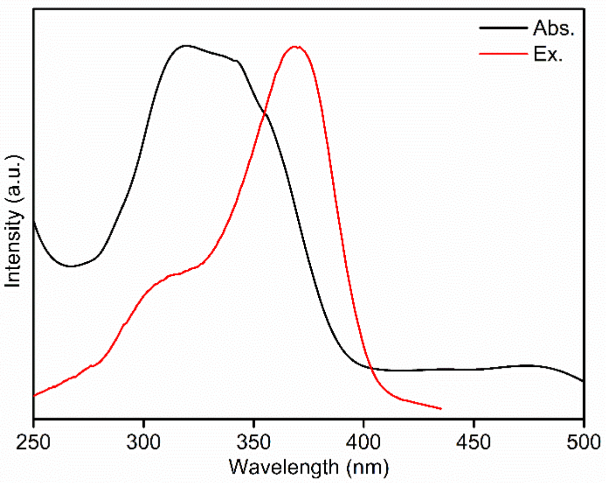

Supplementary Fig. 115. The absorption and excitation spectra of **3b**$^{\mathbf{III}}$ (λ_{em} = 444 nm) in THF at room temperature.

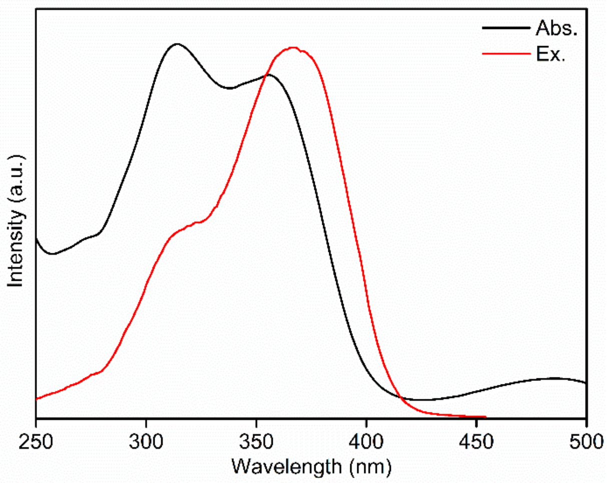

Supplementary Fig. 116. The absorption and excitation spectra of **3b**$^{\mathbf{IV}}$ (λ_{em} = 464 nm) in THF at room temperature.

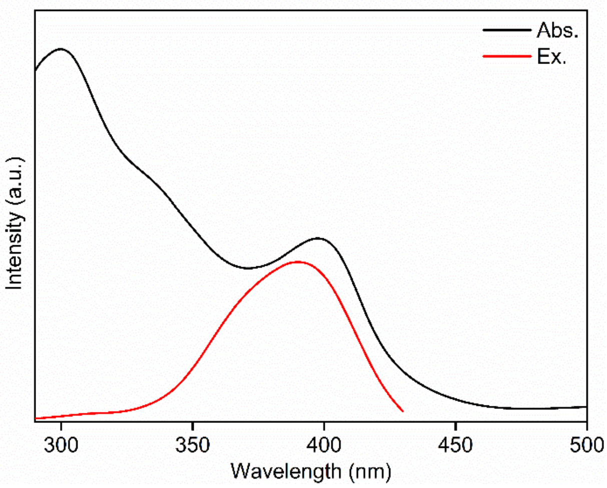

Supplementary Fig. 117. The absorption and excitation spectra of **3c**$^{\mathbf{I}}$ (λ_{em} = 484 nm) in THF at room temperature.

4. Details of Single Crystal X-Ray Diffraction Studies

Table S1. X-Ray crystallographic details of **1a**$^{\mathbf{I}}$ and **1a**$^{\mathbf{II}}$.

Compound	**1a**$^{\mathbf{I}}$	**1a**$^{\mathbf{II}}$
Empirical formula	$C_{47}H_{54}N_3I$	$C_{55}H_{68}N_3O_4F_2Br$
Formula weight	787.83	953.03
Radiation / λ	Mo K_α / (0.71073 Å)	Mo K_α / (0.71073)
Temperature/K	180	170
Crystal system	monoclinic	monoclinic
Space group	$P2_1/c$	$C2/c$
a/Å	10.6380(7)	14.458(2)
b/Å	18.7649(10)	22.289(3)
c/Å	21.3848(11)	16.830(2)
α/°	90	90
β/°	97.819(2)	110.908(4)
γ/°	90	90
V/Å^3	4229.2(4)	5066.5(12)
Z	4	4
P calcg/cm^3	1.237	1.249
μ/mm^{-1}	0.791	0.866
F(000)	1640	2016.0
2Θ range for data collection/°	5.036 to 56.54	4.48 to 51.336
Index ranges	$-12 \leq h \leq 14$, $-25 \leq k \leq 24$, $-27 \leq l \leq 28$	$-13 \leq h \leq 17$, $-27 \leq k \leq 27$, $-20 \leq l \leq 19$
Independent reflections	10401 [R_{int} = 0.0316]	4679 [R_{int} = 0.0581]
Data/restraints/parameters	10401/6/479	4679/0/301
Goodness-of-fit on F^2	1.066	1.057
Final R indexes [I>=2σ (I)]	R_1 = 0.0320, wR_2 = 0.0713	R_1 = 0.0987, wR_2 = 0.2668
Final R indexes [all data]	R_1 = 0.0454, wR_2 = 0.0775	R_1 = 0.1534, wR_2 = 0.2959
Largest diff. peak/hole / e Å^{-3}	0.48/-0.46	1.34/-0.98
CCDC number	2107665	2160168

Table S2. X-Ray crystallographic details of **1a^III^** and **1a^IV^**.

Compound	**1a^III^**	**1a^IV^**
Empirical formula	$C_{49}H_{52}N_3F_6Br$	$C_{49}H_{52}N_5Br$
Formula weight	876.84	790.86
Radiation / λ	Mo K_α / (0.71073)	Mo K_α / (0.71073)
Temperature/K	170	195
Crystal system	tetragonal	monoclinic
Space group	$P4_12_12$	$C2/c$
a/Å	12.1202(6)	11.6253(6)
b/Å	12.1202(6)	26.6663(11)
c/Å	64.304(3)	14.8771(6)
α/°	90	90
β/°	90	108.2900(10)
γ/°	90	90
V/Å^3	9446.2(10)	4379.0(3)
Z	8	4
P calcg/cm^3	1.233	1.200
μ/mm^{-1}	0.929	0.977
F(000)	3648.0	1664.0
2Θ range for data collection/°	4.618 to 51.344	4.194 to 54.18
Index ranges	$-13 \leq h \leq 14$, $-14 \leq k \leq 13$, $-78 \leq l \leq 78$	$-14 \leq h \leq 8$, $-34 \leq k \leq 34$, $-19 \leq l \leq 19$
Independent reflections	8946 [R_{int} = 0.0844]	4807 [R_{int} = 0.0394]
Data/restraints/parameters	8946/1/548	4807/0/256
Goodness-of-fit on F^2	1.026	1.032
Final R indexes [I>=2σ (I)]	R_1 = 0.0502, wR_2 = 0.1154	R_1 = 0.0446, wR_2 = 0.1064
Final R indexes [all data]	R_1 = 0.0952, wR_2 = 0.1336	R_1 = 0.0658, wR_2 = 0.1167
Largest diff. peak/hole / e Å^{-3}	0.35/-0.48	0.55/-0.50
CCDC number	2160169	2160170

Table S3. X-Ray crystallographic details of **1b^{I}** and **1b^{II}**.

Compound	**1b^{I}**	**1b^{II}**
Empirical formula	$C_{45}H_{50}N_3I$	$C_{49}H_{54}N_5F_2Br$
Formula weight	759.78	830.88
Radiation / λ	Cu K_{α} / (1.54178 Å)	Mo K_{α} / (0.71073 Å)
Temperature/K	160	192
Crystal system	monoclinic	triclinic
Space group	$P2_1/c$	P-1
a/Å	10.2334(17)	10.6941(10)
b/Å	19.180(3)	14.0235(14)
c/Å	20.152(3)	16.0131(15)
α/°	90	87.331(3)
β/°	90.650(5)	71.916(3)
γ/°	90	83.307(3)
V/Å^3	3955.2(11)	2267.1(4)
Z	4	2
P calcg/cm^3	1.276	1.217
μ/mm^{-1}	6.604	0.953
F(000)	1576	872.0
2Θ range for data collection/°	6.362 to 136.56	4.718 to 52.776
Index ranges	$-12 \le h \le 12$, $-22 \le k \le 22$, $-24 \le l \le 23$	$-13 \le h \le 13$, $-17 \le k \le 17$, $-20 \le l \le 19$
Independent reflections	7105 [R_{int} = 0.0330]	9141 [R_{int} = 0.0454]
Data/restraints/parameters	7105/0/450	9141/0/524
Goodness-of-fit on F^2	1.072	1.050
Final R indexes [I>=2σ (I)]	R_1 = 0.0363, wR_2 = 0.0959	R_1 = 0.0403, wR_2 = 0.1033
Final R indexes [all data]	R_1 = 0.0379, wR_2 = 0.0973	R_1 = 0.0567, wR_2 = 0.1131
Largest diff. peak/hole / e Å^{-3}	1.23/-1.04	0.54/-0.69
CCDC number	2107667	2160163

Table S4. X-Ray crystallographic details of $\mathbf{1b^{III}}$ and $\mathbf{1b^{IV}}$.

Compound	$\mathbf{1b^{III}}$	$\mathbf{1b^{IV}}$
Empirical formula	$C_{49}H_{51}N_4F_6Br$	$C_{47}H_{48}N_5Br$
Formula weight	889.84	762.81
Radiation / λ	Mo K_α / (0.71073 Å)	Mo K_α / (0.71073 Å)
Temperature/K	193	192
Crystal system	monoclinic	monoclinic
Space group	$C2/c$	$P2_1$/n
a/Å	26.5413(13)	10.7622(3)
b/Å	17.4606(9)	19.7009(6)
c/Å	21.7074(13)	20.2854(7)
α/°	90	90
β/°	112.499(2)	94.9730(10)
γ/°	90	90
V/Å^3	9294.1(9)	4284.8(2)
Z	8	4
P calcg/cm^3	1.272	1.182
μ/mm^{-1}	0.945	0.996
F(000)	3696.0	1600.0
2Θ range for data collection/°	3.86 to 54.176	4.134 to 54.202
Index ranges	$-30 \le h \le 33$, $-22 \le k \le 17$, $-27 \le l \le 25$	$-13 \le h \le 12$, $-21 \le k \le 25$, $-25 \le l \le 25$
Independent reflections	10179 [R_{int} = 0.0551]	9381 [R_{int} = 0.0413]
Data/restraints/parameters	10179/22/568	9381/18/506
Goodness-of-fit on F^2	1.029	1.031
Final R indexes [I>=2σ (I)]	R_1 = 0.0684, wR_2 = 0.1891	R_1 = 0.0636, wR_2 = 0.1573
Final R indexes [all data]	R_1 = 0.1341, wR_2 = 0.2290	R_1 = 0.0986, wR_2 = 0.1773
Largest diff. peak/hole / e Å^{-3}	0.81/-0.44	0.80/-0.53
CCDC number	2160165	2160166

Table S5. X-Ray crystallographic details of **1c^I** and **2a**.

Compound	**1c^I**	**2a**
Empirical formula	$C_{45}H_{52}N_3I$	$C_{49}H_{54}Cl_6IN_3$
Formula weight	761.79	1024.55
Radiation / λ	Mo K_α / (0.71073 Å)	Mo K_α / (0.71073)
Temperature/K	150	150
Crystal system	monoclinic	monoclinic
Space group	$P2_1$/n	$P2_1/c$
a/Å	16.6046(7)	10.6706(5)
b/Å	14.1312(6)	19.8132(7)
c/Å	17.5045(8)	23.1398(9)
α/°	90	90
β/°	98.3550(10)	95.4870(19)
γ/°	90	90
V/Å^3	4063.7(3)	4869.8(3)
Z	4	4
P calcg/cm^3	1.245	1.397
μ/mm^{-1}	0.821	1.023
F(000)	1584	2096
2Θ range for data collection/°	4.656 to 56.56	4.834 to 50.704
Index ranges	$-22 \leq h \leq 18$, $-18 \leq k \leq 18$, $-23 \leq l \leq 22$	$-12 \leq h \leq 11$, $-23 \leq k \leq 23$, $-27 \leq l \leq 27$
Independent reflections	9995 [R_{int} = 0.0268]	8883 [R_{int} = 0.0375]
Data/restraints/parameters	9995/0/450	8883/0/542
Goodness-of-fit on F^2	1.038	1.033
Final R indexes [I>=2σ (I)]	R_1 = 0.0270, wR_2 = 0.0607	R_1 = 0.0355, wR_2 = 0.0880
Final R indexes [all data]	R_1 = 0.0338, wR_2 = 0.0645	R_1 = 0.0464, wR_2 = 0.0957
Largest diff. peak/hole / e Å^{-3}	0.43/-0.71	0.54/-0.73
CCDC number	2107668	2107672

Table S6. X-Ray crystallographic details of **2b** and **2c**.

Compound	**2b**	**2c**
Empirical formula	$C_{45}H_{48}IN_3$	$C_{47}H_{52}Cl_6IN_3$
Formula weight	757.76	998.51
Radiation / λ	Mo K_α / (0.71073)	Mo Kα (λ = 0.71073)
Temperature/K	150	150
Crystal system	monoclinic	monoclinic
Space group	*C*2/*c*	$P2_1/c$
a/Å	24.6751(8)	10.6969(8)
b/Å	19.0836(8)	19.3188(14)
c/Å	18.8533(8)	23.0950(13)
α/°	90	90
β/°	114.6190(10)	95.460(2)
γ/°	90	90
V/Å^3	8070.8(6)	4751.4(6)
Z	8	4
P calcg/cm^3	1.247	1.396
μ/mm^{-1}	0.826	1.046
F(000)	3136	2040
2Θ range for data collection/°	5.394 to 50.73	4.848 to 50.738
Index ranges	-29 ≤ h ≤ 28, -22 ≤ k ≤ 22, -22 ≤ l ≤ 22	-12 ≤ h ≤ 12, -23 ≤ k ≤ 23, -27 ≤ l ≤ 27
Independent reflections	7343 [R_{int} = 0.0330]	8673 [R_{int} = 0.0348]
Data/restraints/parameters	7343/83/455	8673/0/522
Goodness-of-fit on F^2	1.057	1.032
Final *R* indexes [I>=2σ (I)]	R_1 = 0.0398, wR_2 = 0.1121	R_1 = 0.0301, wR_2 = 0.0672
Final *R* indexes [all data]	R_1 = 0.0448, wR_2 = 0.1165	R_1 = 0.0381, wR_2 = 0.0721
Largest diff. peak/hole / e Å^{-3}	1.81/-0.70	0.61/-0.65
CCDC number	2107673	2107674

Table S7. X-Ray crystallographic details of **3a^I^** and **3b^I^**.

Compound	**3a**$^{\mathbf{I}}$	**3b**$^{\mathbf{I}}$
Empirical formula	$C_{47}H_{54}N_3$	$C_{45}H_{50}N_3$
Formula weight	660.93	632.88
Radiation / *λ*	Mo K_α / (0.71073)	Mo K_α / (0.71073)
Temperature/K	180	150
Crystal system	monoclinic	orthorhombic
Space group	*I*2/a	$P2_12_12_1$
a/Å	19.414(6)	10.1623(4)
b/Å	24.871(7)	17.1893(8)
c/Å	24.832(7)	21.5680(9)
α/°	90	90
β/°	103.43(2)	90
γ/°	90	90
V/Å^3	11662(6)	3767.6(3)
Z	12	4
P calcg/cm^3	1.129	1.116
μ/mm^{-1}	0.065	0.064
F(000)	4284	1364
2*Θ* range for data collection/°	4.70 to 50.342	3.03 to 54.252
Index ranges	$-21 \leq h \leq 23$, $-29 \leq k \leq 29$, $-29 \leq l \leq 28$	$-12 \leq h \leq 13$, $-21 \leq k \leq 21$, $-27 \leq l \leq 27$
Independent reflections	10327 [R_{int} = 0.0518]	8263 [R_{int} = 0.0397]
Data/restraints/parameters	10327/40/715	8263/0/441
Goodness-of-fit on F^2	1.015	1.027
Final *R* indexes [I>=2σ (I)]	R_1 = 0.0500, wR_2 = 0.1086	R_1 = 0.0373, wR_2 = 0.0890
Final *R* indexes [all data]	R_1 = 0.0938, wR_2 = 0.1306	R_1 = 0.0462, wR_2 = 0.0950
Largest diff. peak/hole / e Å^{-3}	0.20/-0.19	0.18/-0.19
CCDC number	2107669	2107670

Table S8. X-Ray crystallographic details of **3b^III^** and **3c^I^**.

Compound	**3b^{III}**	**3c^{I}**
Empirical formula	$C_{47}H_{48}N_3F_6$	$C_{45}H_{52}N_3$
Formula weight	768.88	634.89
Radiation / λ	Mo K_α / (0.71073)	Mo K_α / (0.71073)
Temperature/K	197	150
Crystal system	triclinic	orthorhombic
Space group	P-1	$P2_12_12_1$
a/Å	12.6028(17)	10.1950(4)
b/Å	12.6843(17)	17.2008(6)
c/Å	13.6272(18)	21.2573(9)
α/°	86.177(4)	90
β/°	73.715(5)	90
γ/°	78.397(5)	90
V/Å^3	2048.2(5)	3727.7(3)
Z	2	4
P calcg/cm^3	1.247	1.131
μ/mm^{-1}	0.092	0.065
F(000)	810.0	1372
2Θ range for data collection/°	3.43 to 54.11	4.504 to 52.726
Index ranges	$-15 \leq h \leq 16$, $-16 \leq k \leq 16$, $-14 \leq l \leq 17$	$-12 \leq h \leq 10$, $-21 \leq k \leq 18$, $-26 \leq l \leq 26$
Independent reflections	8872 [R_{int} = 0.0298]	7606 [R_{int} = 0.0630]
Data/restraints/parameters	8872/72/567	7606/0/441
Goodness-of-fit on F^2	1.025	1.020
Final R indexes [I>=2σ (I)]	R_1 = 0.0511, wR_2 = 0.1158	R_1 = 0.0505, wR_2 = 0.0952
Final R indexes [all data]	R_1 = 0.0729, wR_2 = 0.1290	R_1 = 0.0953, wR_2 = 0.1146
Largest diff. peak/hole / e Å^{-3}	0.41/-0.36	0.19/-0.18
CCDC number	2160172	2107671

Table S9. X-Ray crystallographic details of **4a** and **4b**.

Compound	**4a**	**4b**
Empirical formula	$C_{50}H_{59}N_3$	$C_{45}H_{48}N_3$
Formula weight	702.00	630.86
Radiation / λ	Mo K_α / (0.71073)	Mo Kα (λ = 0.71073)
Temperature/K	180	190
Crystal system	monoclinic	monoclinic
Space group	$C2/c$	$P2_1/c$
a/Å	10.895(4)	16.5543(9)
b/Å	26.906(7)	11.3572(7)
c/Å	15.974(4)	20.1620(2)
α/°	90	90
β/°	103.207(13)	106.966(2)
γ/°	90	90
V/Å^3	4558(2)	3625.7(4)
Z	4	4
P calcg/cm^3	1.023	1.156
μ/mm^{-1}	0.059	0.067
F(000)	1520	1356
2Θ range for data collection/°	5.338 to 50.716	5.146 to 50.688
Index ranges	-13 ≤ h ≤ 13, -32 ≤ k ≤ 32, -19 ≤ l ≤ 19	-19 ≤ h ≤ 19, -13 ≤ k ≤ 13, -24 ≤ l ≤ 24
Independent reflections	4162 [R_{int} = 0.0384]	6617 [R_{int} = 0.0530]
Data/restraints/parameters	4162/42/290	6617/6/441
Goodness-of-fit on F^2	1.048	1.035
Final R indexes [I>=2σ (I)]	R_1 = 0.0601, wR_2 = 0.1651	R_1 = 0.0534, wR_2 = 0.1204
Final R indexes [all data]	R_1 = 0.0789, wR_2 = 0.1816	R_1 = 0.0790, wR_2 = 0.1370
Largest diff. peak/hole / e Å^{-3}	0.34/-0.24	0.52/-0.39
CCDC number	2107675	2107676

Table S10. X-Ray crystallographic details of **4c**.

Compound	**4c**
Empirical formula	$C_{45}H_{50}N_3$
Formula weight	632.88
Radiation / λ	Mo K_α / (0.71073)
Temperature/K	190
Crystal system	orthorhombic
Space group	$Pna2_1$
a/Å	19.5022(9)
b/Å	11.3064(7)
c/Å	34.698(2)
α/°	90
β/°	90
γ/°	90
V/Å^3	7650.9(8)
Z	4
P calcg/cm^3	1.099
μ/mm^{-1}	0.064
F(000)	2728
2Θ range for data collection/°	4.78 to 50.682
Index ranges	$-22 \le h \le 23$, $-13 \le k \le 13$, $-41 \le l \le 38$
Independent reflections	12309 [R_{int} = 0.0603]
Data/restraints/parameters	12309/50/911
Goodness-of-fit on F^2	1.031
Final R indexes [I>=2σ (I)]	R_1 = 0.0493, wR_2 = 0.1004
Final R indexes [all data]	R_1 = 0.0845, wR_2 = 0.1175
Largest diff. peak/hole / e Å^{-3}	0.16/-0.20
CCDC number	2107677

5. Cartesian coordinates of the optimized geometries by DFT calculations

3a^I (UB3LYP/6-31G(d))

Center Number	Atomic Number	Atomic Type	Coordinates (Angstroms) X	Y	Z
1	7	0	-2.477391	1.069873	-0.319333
2	7	0	-2.477326	-1.069841	0.320147
3	7	0	4.133311	0.000005	-0.000007
4	6	0	-2.151845	2.465959	-0.438009
5	6	0	-3.818888	0.650399	-0.182482
6	6	0	-1.608994	0.000007	0.000274
7	6	0	-2.151754	-2.465984	0.438180
8	6	0	-3.818863	-0.650361	0.183501
9	6	0	-0.189255	0.000013	0.000196
10	6	0	4.825016	-1.080536	-0.599052
11	6	0	2.705691	0.000005	0.000052
12	6	0	1.972761	-1.092152	0.501867
13	1	0	2.507399	-1.949988	0.901247
14	6	0	1.972717	1.092146	-0.501716
15	1	0	2.507312	1.949986	-0.901147
16	6	0	-2.126171	3.051794	-1.725127
17	6	0	-1.916509	3.227511	0.728213
18	6	0	-1.916668	-3.227092	-0.728394
19	6	0	0.590336	-1.102739	0.500660
20	1	0	0.100992	-1.975874	0.903959
21	6	0	0.590289	1.102743	-0.500384
22	1	0	0.100929	1.975876	-0.903669
23	6	0	-4.964144	1.579048	-0.415969
24	1	0	-4.902509	2.471975	0.217509
25	1	0	-5.907886	1.075242	-0.193343
26	1	0	-5.008445	1.927776	-1.454597
27	6	0	-2.125697	-3.052319	1.725063
28	6	0	4.825079	1.080602	0.598863
29	6	0	4.307210	-1.703454	-1.748878
30	1	0	3.384247	-1.331630	-2.181590
31	6	0	-2.346289	-2.240931	3.000552
32	1	0	-2.672207	-1.239222	2.704177
33	6	0	-1.842782	4.419645	-1.818015
34	1	0	-1.807245	4.892046	-2.795786
35	6	0	-1.634766	4.590752	0.576664
36	1	0	-1.444828	5.196401	1.458668
37	6	0	-2.347154	2.239895	-3.000220
38	1	0	-2.673115	1.238346	-2.703349

39	6	0	4.307311	1.703706	1.748608
40	1	0	3.384331	1.331988	2.181378
41	6	0	-4.964104	-1.579011	0.417004
42	1	0	-4.903675	-2.470960	-0.217987
43	1	0	-5.907959	-1.074516	0.196434
44	1	0	-5.007139	-1.929371	1.455122
45	6	0	-1.597151	5.184512	-0.681187
46	1	0	-1.375425	6.244273	-0.776258
47	6	0	-1.842235	-4.420197	1.817353
48	1	0	-1.806440	-4.892972	2.794932
49	6	0	-1.955087	-2.618585	-2.127552
50	1	0	-2.184793	-1.554257	-2.027202
51	6	0	-1.634787	-4.590364	-0.577434
52	1	0	-1.445028	-5.195663	-1.459712
53	6	0	-1.954555	2.619513	2.127609
54	1	0	-2.183852	1.555060	2.027667
55	6	0	-1.036106	2.077758	-3.797180
56	1	0	-0.648862	3.050128	-4.124674
57	1	0	-1.209770	1.468898	-4.692722
58	1	0	-0.260450	1.589619	-3.199383
59	6	0	6.021759	1.575423	0.048051
60	1	0	6.426707	1.115760	-0.847684
61	6	0	-3.445726	2.842237	-3.897692
62	1	0	-4.389015	2.976321	-3.356633
63	1	0	-3.636063	2.183537	-4.752990
64	1	0	-3.152439	3.819990	-4.297446
65	6	0	6.021666	-1.575498	-0.048307
66	1	0	6.426643	-1.115976	0.847489
67	6	0	-1.596846	-5.184617	0.680178
68	1	0	-1.375058	-6.244406	0.774788
69	6	0	4.968596	-2.784795	-2.326281
70	1	0	4.549844	-3.248293	-3.216322
71	6	0	6.162545	-3.264745	-1.783417
72	1	0	6.677803	-4.105536	-2.239276
73	6	0	6.682231	2.648390	0.642275
74	1	0	7.604779	3.013988	0.197762
75	6	0	6.162731	3.264902	1.782931
76	1	0	6.678039	4.105727	2.238673
77	6	0	4.968758	2.785088	2.325862
78	1	0	4.550033	3.248728	3.215842
79	6	0	6.682084	-2.648418	-0.642678
80	1	0	7.604614	-3.014122	-0.198215
81	6	0	-1.035041	-2.079262	3.797275

82	1	0	-0.647912	-3.051772	4.124499
83	1	0	-1.208391	-1.470547	4.692972
84	1	0	-0.259419	-1.591128	3.199431
85	6	0	-3.444679	-2.843537	3.898070
86	1	0	-4.388271	-2.976884	3.357358
87	1	0	-3.634403	-2.185399	4.753939
88	1	0	-3.151561	-3.821693	4.296953
89	6	0	-3.066002	-3.250081	-2.989474
90	1	0	-2.894209	-4.321334	-3.147180
91	1	0	-3.100478	-2.771138	-3.975182
92	1	0	-4.051477	-3.135712	-2.523928
93	6	0	-0.585932	2.728109	2.828276
94	1	0	0.202579	2.266529	2.226332
95	1	0	-0.617459	2.222579	3.800836
96	1	0	-0.309228	3.774120	3.007207
97	6	0	-0.586484	-2.726405	-2.828377
98	1	0	0.201904	-2.264680	-2.226384
99	1	0	-0.618307	-2.220608	-3.800793
100	1	0	-0.309375	-3.772255	-3.007621
101	6	0	-3.065657	3.250935	2.989341
102	1	0	-2.894506	4.322420	3.146161
103	1	0	-3.099597	2.772722	3.975423
104	1	0	-4.051169	3.135569	2.524117

3a^{II} (UB3LYP/6-31G(d))

Center	Atomic	Atomic	Coordinates (Angstroms)		
Number	Number	Type	X	Y	Z
1	7	0	-2.775777	1.042516	-0.400523
2	7	0	-2.775839	-1.042503	0.400568
3	7	0	3.832757	-0.000015	0.000019
4	6	0	-2.446730	2.427139	-0.612189
5	6	0	-4.117515	0.634642	-0.231848
6	6	0	-1.908557	-0.000007	0.000048
7	6	0	-2.446858	-2.427147	0.612206
8	6	0	-4.117553	-0.634587	0.231777
9	6	0	-0.488876	-0.000017	0.000058
10	6	0	4.530518	-1.156138	-0.427631
11	6	0	2.405921	-0.000015	0.000035
12	6	0	1.672532	-1.036550	0.609285
13	1	0	2.206061	-1.849594	1.094523
14	6	0	1.672521	1.036521	-0.609198
15	1	0	2.206041	1.849566	-1.094445

16	6	0	-2.424539	2.927270	-1.935103
17	6	0	-2.200334	3.262345	0.500083
18	6	0	-2.200406	-3.262294	-0.500094
19	6	0	0.290325	-1.048366	0.606319
20	1	0	-0.200349	-1.877139	1.093170
21	6	0	0.290314	1.048335	-0.606207
22	1	0	-0.200373	1.877110	-1.093047
23	6	0	-5.262193	1.544969	-0.530614
24	1	0	-5.192985	2.484286	0.030853
25	1	0	-6.205685	1.063344	-0.262439
26	1	0	-5.312907	1.811327	-1.593020
27	6	0	-2.424688	-2.927338	1.935100
28	6	0	4.530528	1.156107	0.427655
29	6	0	4.038649	-1.931727	-1.492988
30	1	0	3.127799	-1.624155	-1.995466
31	6	0	-2.665459	-2.036633	3.152954
32	1	0	-3.000285	-1.060411	2.788964
33	6	0	-2.129471	4.283414	-2.119418
34	1	0	-2.095711	4.689494	-3.126640
35	6	0	-1.908223	4.610181	0.257624
36	1	0	-1.708305	5.270378	1.097213
37	6	0	-2.665358	2.036506	-3.152906
38	1	0	-3.000032	1.060257	-2.788846
39	6	0	4.038688	1.931692	1.493028
40	1	0	3.127853	1.624117	1.995532
41	6	0	-5.262288	-1.544886	0.530406
42	1	0	-5.193026	-2.484213	-0.031036
43	1	0	-6.205734	-1.063246	0.262098
44	1	0	-5.313149	-1.811224	1.592809
45	6	0	-1.870714	5.119269	-1.036789
46	1	0	-1.639831	6.168291	-1.202557
47	6	0	-2.129606	-4.283487	2.119357
48	1	0	-2.095849	-4.689609	3.126563
49	6	0	-2.236147	-2.749684	-1.937344
50	1	0	-2.472127	-1.682492	-1.910867
51	6	0	-1.908287	-4.610139	-0.257694
52	1	0	-1.708314	-5.270287	-1.097308
53	6	0	-2.236200	2.749839	1.937368
54	1	0	-2.472115	1.682632	1.910950
55	6	0	-1.364182	1.808312	-3.949723
56	1	0	-0.966751	2.754205	-4.336945
57	1	0	-1.554492	1.150636	-4.806534
58	1	0	-0.589630	1.344758	-3.331176

59	6	0	5.711521	1.571296	-0.214848
60	1	0	6.097309	0.993814	-1.048337
61	6	0	-3.765434	2.592972	-4.077839
62	1	0	-4.700863	2.776123	-3.537627
63	1	0	-3.973508	1.881532	-4.885395
64	1	0	-3.463459	3.537820	-4.544434
65	6	0	5.711531	-1.571322	0.214838
66	1	0	6.097342	-0.993837	1.048313
67	6	0	-1.870820	-5.119291	1.036696
68	1	0	-1.639921	-6.168316	1.202420
69	6	0	4.702319	-3.085543	-1.903442
70	1	0	4.327378	-3.686985	-2.725474
71	6	0	5.874253	-3.459713	-1.256957
72	6	0	6.390232	2.713342	0.204234
73	1	0	7.301525	3.037764	-0.288045
74	6	0	5.874282	3.459683	1.256946
75	6	0	4.702367	3.085508	1.903464
76	1	0	4.327449	3.686948	2.725509
77	6	0	6.390232	-2.713368	-0.204262
78	1	0	7.301540	-3.037786	0.287991
79	6	0	-1.364206	-1.808318	3.949610
80	1	0	-0.966633	-2.754172	4.336783
81	1	0	-1.554470	-1.150660	4.806445
82	1	0	-0.589778	-1.344690	3.330963
83	6	0	-3.765344	-2.593262	4.078016
84	1	0	-4.700825	-2.776510	3.537929
85	1	0	-3.973394	-1.881876	4.885626
86	1	0	-3.463190	-3.538092	4.544535
87	6	0	-3.339713	-3.444581	-2.759079
88	1	0	-3.161997	-4.523143	-2.842940
89	1	0	-3.372048	-3.033951	-3.775203
90	1	0	-4.327887	-3.303813	-2.306534
91	6	0	-0.863682	2.896095	2.623296
92	1	0	-0.082056	2.388877	2.049673
93	1	0	-0.893872	2.457590	3.627859
94	1	0	-0.578789	3.949749	2.729697
95	6	0	-0.863542	-2.895800	-2.623129
96	1	0	-0.082016	-2.388567	-2.049382
97	1	0	-0.893648	-2.457227	-3.627665
98	1	0	-0.578564	-3.949426	-2.729571
99	6	0	-3.339898	3.444731	2.758929
100	1	0	-3.162257	4.523309	2.842732
101	1	0	-3.372322	3.034174	3.775080

102	1	0	-4.328014	3.303870	2.306284
103	9	0	6.525504	-4.576580	-1.659172
104	9	0	6.525541	4.576551	1.659144

3a^III^ (UB3LYP/6-31G(d))

Center	Atomic	Atomic	Coordinates (Angstroms)		
Number	Number	Type	X	Y	Z
1	7	0	-3.540572	0.933121	-0.610754
2	7	0	-3.540550	-0.933176	0.610743
3	7	0	3.066175	0.000027	0.000009
4	6	0	-3.205424	2.235034	-1.125297
5	6	0	-4.881927	0.571714	-0.360597
6	6	0	-2.675761	-0.000011	0.000003
7	6	0	-3.205353	-2.235070	1.125301
8	6	0	-4.881914	-0.571840	0.360525
9	6	0	-1.255579	0.000009	0.000013
10	6	0	3.752189	-1.206894	-0.254936
11	6	0	1.634795	0.000025	0.000014
12	6	0	0.904477	-0.865143	0.836750
13	1	0	1.438705	-1.540534	1.499989
14	6	0	0.904467	0.865185	-0.836723
15	1	0	1.438688	1.540577	-1.499966
16	6	0	-3.189285	2.431021	-2.525980
17	6	0	-2.943390	3.290915	-0.224607
18	6	0	-2.943213	-3.290935	0.224621
19	6	0	-0.477213	-0.875915	0.837759
20	1	0	-0.967360	-1.565192	1.508034
21	6	0	-0.477222	0.875941	-0.837732
22	1	0	-0.967377	1.565204	-1.508015
23	6	0	-6.027310	1.399128	-0.841874
24	1	0	-5.961715	2.433749	-0.484728
25	1	0	-6.969884	0.980331	-0.481259
26	1	0	-6.076801	1.440918	-1.936099
27	6	0	-3.189237	-2.431054	2.525987
28	6	0	3.752190	1.206949	0.254947
29	6	0	3.210331	-2.155228	-1.143325
30	1	0	2.277424	-1.933794	-1.649793
31	6	0	-3.457232	-1.300327	3.518002
32	1	0	-3.796856	-0.430797	2.946934
33	6	0	-2.877524	3.709084	-3.004987
34	1	0	-2.847453	3.883968	-4.076785
35	6	0	-2.635244	4.547976	-0.759074

36	1	0	-2.423706	5.374305	-0.086046
37	6	0	-3.457187	1.300277	-3.518001
38	1	0	-3.796711	0.430708	-2.946935
39	6	0	3.210340	2.155283	1.143341
40	1	0	2.277438	1.933850	1.649817
41	6	0	-6.027276	-1.399313	0.841749
42	1	0	-5.961584	-2.433941	0.484642
43	1	0	-6.969852	-0.980592	0.481053
44	1	0	-6.076842	-1.441070	1.935972
45	6	0	-2.598581	4.758475	-2.133912
46	1	0	-2.354627	5.741646	-2.527528
47	6	0	-2.877396	-3.709094	3.005003
48	1	0	-2.847339	-3.883973	4.076803
49	6	0	-2.985729	-3.109614	-1.290266
50	1	0	-3.241013	-2.067229	-1.500126
51	6	0	-2.634994	-4.547973	0.759100
52	1	0	-2.423374	-5.374287	0.086079
53	6	0	-2.985955	3.109589	1.290278
54	1	0	-3.241145	2.067178	1.500125
55	6	0	-2.171585	0.885624	-4.263474
56	1	0	-1.770836	1.717798	-4.854645
57	1	0	-2.382740	0.058524	-4.951811
58	1	0	-1.391656	0.558837	-3.569140
59	6	0	4.965431	1.511196	-0.392593
60	1	0	5.392351	0.802261	-1.093653
61	6	0	-4.564965	1.658380	-4.527811
62	1	0	-5.488967	1.970712	-4.028973
63	1	0	-4.795961	0.791438	-5.157654
64	1	0	-4.258331	2.473428	-5.193677
65	6	0	4.965435	-1.511142	0.392594
66	1	0	5.392361	-0.802206	1.093650
67	6	0	-2.598356	-4.758468	2.133939
68	1	0	-2.354343	-5.741621	2.527564
69	6	0	3.861315	-3.359786	-1.376877
70	1	0	3.435784	-4.073483	-2.075240
71	6	0	5.071295	-3.649809	-0.737809
72	6	0	5.615909	2.714267	-0.147473
73	1	0	6.550348	2.931801	-0.654956
74	6	0	5.071302	3.649863	0.737809
75	6	0	3.861327	3.359841	1.376887
76	1	0	3.435802	4.073539	2.075255
77	6	0	5.615911	-2.714213	0.147469
78	1	0	6.550354	-2.931749	0.654943

79	6	0	-2.171654	-0.885536	4.263442
80	1	0	-1.770805	-1.717663	4.854611
81	1	0	-2.382880	-0.058452	4.951778
82	1	0	-1.391776	-0.558670	3.569087
83	6	0	-4.564946	-1.658535	4.527844
84	1	0	-5.488930	-1.970967	4.029034
85	1	0	-4.796014	-0.791611	5.157686
86	1	0	-4.258209	-2.473546	5.193708
87	6	0	-4.077935	-3.987318	-1.933037
88	1	0	-3.876213	-5.054790	-1.786408
89	1	0	-4.125336	-3.804335	-3.013014
90	1	0	-5.066313	-3.773914	-1.510338
91	6	0	-1.611703	3.381934	1.933082
92	1	0	-0.838337	2.745540	1.492422
93	1	0	-1.651194	3.181102	3.010257
94	1	0	-1.307610	4.427268	1.801556
95	6	0	-1.611422	-3.381826	-1.933005
96	1	0	-0.838139	-2.745355	-1.492310
97	1	0	-1.650882	-3.180999	-3.010183
98	1	0	-1.307233	-4.427129	-1.801462
99	6	0	-4.078275	3.987184	1.933003
100	1	0	-3.876656	5.054676	1.786379
101	1	0	-4.125701	3.804199	3.012978
102	1	0	-5.066614	3.773679	1.510265
103	6	0	5.737932	-4.975851	-0.944711
104	6	0	5.737944	4.975904	0.944704
105	9	0	5.508698	-5.472174	-2.183625
106	9	0	7.079510	-4.901416	-0.781057
107	9	0	5.295655	-5.913903	-0.068542
108	9	0	5.295658	5.913957	0.068540
109	9	0	5.508723	5.472228	2.183621
110	9	0	7.079519	4.901467	0.781036

3a^{IV} (UB3LYP/6-31G(d))

Center	Atomic	Atomic	Coordinates (Angstroms)		
Number	Number	Type	X	Y	Z
1	7	0	-2.926866	0.995154	-0.500090
2	7	0	-2.926988	-0.995052	0.500026
3	7	0	3.675769	-0.000067	0.000047
4	6	0	-2.586719	2.338395	-0.891329
5	6	0	-4.268179	0.609072	-0.294136
6	6	0	-2.063949	0.000020	0.000006

7	6	0	-2.586984	-2.338317	0.891308
8	6	0	-4.268253	-0.608894	0.293880
9	6	0	-0.643330	-0.000017	0.000031
10	6	0	4.359347	-1.168394	-0.392361
11	6	0	2.243456	-0.000051	0.000042
12	6	0	1.514753	-0.935023	0.760492
13	1	0	2.050023	-1.662766	1.364998
14	6	0	1.514782	0.934939	-0.760413
15	1	0	2.050074	1.662668	-1.364916
16	6	0	-2.591116	2.670154	-2.266283
17	6	0	-2.296138	3.297697	0.103569
18	6	0	-2.296352	-3.297621	-0.103572
19	6	0	0.133659	-0.943879	0.762155
20	1	0	-0.358709	-1.682302	1.375856
21	6	0	0.133689	0.943826	-0.762085
22	1	0	-0.358664	1.682261	-1.375791
23	6	0	-5.412659	1.489374	-0.672545
24	1	0	-5.336077	2.477424	-0.203449
25	1	0	-6.354563	1.038784	-0.350620
26	1	0	-5.472290	1.652053	-1.754751
27	6	0	-2.591525	-2.670094	2.266260
28	6	0	4.359367	1.168249	0.392457
29	6	0	3.805046	-2.015956	-1.372230
30	1	0	2.864668	-1.742861	-1.837614
31	6	0	-2.888566	-1.645484	3.360672
32	1	0	-3.241573	-0.730291	2.875334
33	6	0	-2.272241	3.985831	-2.623409
34	1	0	-2.258514	4.264628	-3.673151
35	6	0	-1.979832	4.597174	-0.311244
36	1	0	-1.745681	5.351353	0.434965
37	6	0	-2.888145	1.645538	-3.360692
38	1	0	-3.241146	0.730348	-2.875343
39	6	0	3.805085	2.015815	1.372332
40	1	0	2.864705	1.742732	1.837721
41	6	0	-5.412837	-1.489149	0.672078
42	1	0	-5.336246	-2.477181	0.202943
43	1	0	-6.354667	-1.038488	0.350038
44	1	0	-5.472636	-1.651880	1.754265
45	6	0	-1.964045	4.941343	-1.659137
46	1	0	-1.713957	5.955549	-1.958804
47	6	0	-2.272748	-3.985791	2.623398
48	1	0	-2.259128	-4.264601	3.673139
49	6	0	-2.318938	-2.972836	-1.594435

50	1	0	-2.572170	-1.915230	-1.708866
51	6	0	-1.980153	-4.597120	0.311253
52	1	0	-1.745962	-5.351299	-0.434943
53	6	0	-2.318923	2.972952	1.594438
54	1	0	-2.572211	1.915360	1.708869
55	6	0	-1.616017	1.282260	-4.154938
56	1	0	-1.204477	2.160895	-4.665978
57	1	0	-1.848399	0.530809	-4.919030
58	1	0	-0.836184	0.874492	-3.504315
59	6	0	5.579673	1.541239	-0.207700
60	1	0	6.009840	0.915891	-0.982284
61	6	0	-3.995693	2.120093	-4.321832
62	1	0	-4.911416	2.397112	-3.788035
63	1	0	-4.244713	1.322961	-5.031731
64	1	0	-3.679256	2.991224	-4.906825
65	6	0	5.579652	-1.541400	0.207789
66	1	0	6.009833	-0.916056	0.982368
67	6	0	-1.964515	-4.941308	1.659142
68	1	0	-1.714503	-5.955530	1.958819
69	6	0	4.450941	-3.184484	-1.745864
70	1	0	4.016937	-3.821960	-2.509794
71	6	0	5.674183	-3.547739	-1.153170
72	6	0	6.228391	2.707128	0.170673
73	1	0	7.163526	2.984634	-0.305630
74	6	0	5.674241	3.547573	1.153263
75	6	0	4.450998	3.184333	1.745965
76	1	0	4.017008	3.821814	2.509900
77	6	0	6.228351	-2.707299	-0.170586
78	1	0	7.163486	-2.984816	0.305711
79	6	0	-1.616443	-1.282207	4.154928
80	1	0	-1.204896	-2.160846	4.665957
81	1	0	-1.848833	-0.530770	4.919031
82	1	0	-0.836612	-0.874423	3.504313
83	6	0	-3.996100	-2.120068	4.321814
84	1	0	-4.911824	-2.397093	3.788023
85	1	0	-4.245125	-1.322949	5.031728
86	1	0	-3.679646	-2.991204	4.906791
87	6	0	-3.401553	-3.786294	-2.331355
88	1	0	-3.198020	-4.862460	-2.285692
89	1	0	-3.437532	-3.500175	-3.389108
90	1	0	-4.395300	-3.616807	-1.901353
91	6	0	-0.936247	3.183330	2.242375
92	1	0	-0.168562	2.596544	1.728821

93	1	0	-0.960439	2.875339	3.294334
94	1	0	-0.635338	4.237361	2.212546
95	6	0	-0.936187	-3.183245	-2.242203
96	1	0	-0.168547	-2.596498	-1.728536
97	1	0	-0.960234	-2.875228	-3.294157
98	1	0	-0.635318	-4.237288	-2.212361
99	6	0	-3.401600	3.786472	2.331199
100	1	0	-3.198019	4.862629	2.285534
101	1	0	-3.437726	3.500382	3.388955
102	1	0	-4.395299	3.617013	1.901074
103	6	0	6.338948	-4.753438	-1.539295
104	7	0	6.878620	-5.735946	-1.855170
105	6	0	6.339024	4.753263	1.539386
106	7	0	6.878712	5.735763	1.855259

3b^{I} (UB3LYP/6-31G(d))

Center Number	Atomic Number	Atomic Type	Coordinates (Angstroms) X	Y	Z
1	7	0	2.664167	1.091680	0.251358
2	7	0	2.664356	-1.091367	-0.251370
3	7	0	-3.926260	-0.000179	-0.000171
4	6	0	1.798524	0.000066	0.000021
5	6	0	2.356486	-2.348207	-0.879321
6	6	0	2.356084	2.348385	0.879475
7	6	0	2.055017	-2.390359	-2.258097
8	6	0	2.428650	-3.522225	-0.096814
9	6	0	3.991167	0.654017	0.156092
10	1	0	4.807850	1.332950	0.343877
11	6	0	2.181416	4.748234	0.727217
12	1	0	2.230323	5.665529	0.146636
13	6	0	0.381646	0.000002	-0.000180
14	6	0	-5.811266	-1.426009	0.676248
15	1	0	-6.205560	-1.379525	-0.333726
16	6	0	-4.621501	0.741665	-0.986510
17	6	0	1.866788	-4.810501	-2.080935
18	1	0	1.672474	-5.771148	-2.550675
19	6	0	-2.499253	-0.000123	-0.000226
20	6	0	2.054435	2.390339	2.258205
21	6	0	2.182113	-4.748047	-0.726811
22	1	0	2.231073	-5.665271	-0.146121
23	6	0	2.428195	3.522521	0.097117
24	6	0	-4.621380	-0.742040	0.986229

25	6	0	2.022377	-1.139951	-3.131038
26	1	0	2.195962	-0.273388	-2.487983
27	6	0	1.865894	4.810488	2.081311
28	1	0	1.671382	5.771049	2.551143
29	6	0	-0.386881	-1.213012	0.046143
30	1	0	0.115534	-2.169697	0.089391
31	6	0	-1.769007	-1.204510	0.034189
32	1	0	-2.306533	-2.148233	0.069852
33	6	0	2.753382	-3.490034	1.394302
34	1	0	2.864299	-2.441972	1.687289
35	6	0	-1.769110	1.204329	-0.034686
36	1	0	-2.306716	2.148001	-0.070425
37	6	0	-5.811300	1.425711	-0.676371
38	1	0	-6.205432	1.379295	0.333670
39	6	0	1.803606	-3.641522	-2.833884
40	1	0	1.565177	-3.700505	-3.892283
41	6	0	-6.478943	-2.160581	1.653477
42	1	0	-7.396361	-2.681428	1.390180
43	6	0	-0.386987	1.212946	-0.046618
44	1	0	0.115360	2.169665	-0.089929
45	6	0	-6.479095	2.160271	-1.653531
46	1	0	-7.396444	2.681179	-1.390116
47	6	0	3.991274	-0.653480	-0.156118
48	1	0	4.808066	-1.332291	-0.343872
49	6	0	2.021687	1.139845	3.131016
50	1	0	2.196260	0.273412	2.488049
51	6	0	-4.116734	0.831517	-2.295958
52	1	0	-3.198933	0.309645	-2.546258
53	6	0	-4.116411	-0.831992	2.295595
54	1	0	-3.198541	-0.310191	2.545786
55	6	0	-5.972182	-2.248545	2.952142
56	1	0	-6.492501	-2.828532	3.709160
57	6	0	-4.784378	-1.581943	3.260955
58	1	0	-4.376116	-1.634543	4.267366
59	6	0	1.802769	3.641410	2.834098
60	1	0	1.564159	3.700214	3.892464
61	6	0	-4.784809	1.581459	-3.261249
62	1	0	-4.376713	1.633993	-4.267732
63	6	0	2.753117	3.490546	-1.393958
64	1	0	2.863825	2.442518	-1.687144
65	6	0	4.088733	4.198407	-1.695478
66	1	0	4.046279	5.263238	-1.437294
67	1	0	4.329358	4.123716	-2.762527

68	1	0	4.915536	3.753314	-1.130177
69	6	0	-5.972529	2.248143	-2.952276
70	1	0	-6.492928	2.828123	-3.709244
71	6	0	0.646685	0.940766	3.796854
72	1	0	0.417162	1.750323	4.500094
73	1	0	0.632026	0.000113	4.359920
74	1	0	-0.150256	0.904493	3.047771
75	6	0	1.611668	-4.082559	2.242942
76	1	0	0.670624	-3.549649	2.070600
77	1	0	1.853564	-4.005290	3.309626
78	1	0	1.445486	-5.142256	2.017039
79	6	0	4.088784	-4.198128	1.696171
80	1	0	4.046213	-5.262974	1.438077
81	1	0	4.329186	-4.123390	2.763267
82	1	0	4.915787	-3.753219	1.131016
83	6	0	3.152875	-1.165289	-4.178956
84	1	0	4.135473	-1.256470	-3.702487
85	1	0	3.146123	-0.241078	-4.769098
86	1	0	3.039554	-2.005200	-4.874753
87	6	0	0.647878	-0.941490	-3.798068
88	1	0	0.419051	-1.751360	-4.501167
89	1	0	0.633454	-0.001069	-4.361530
90	1	0	-0.149648	-0.905078	-3.049617
91	6	0	3.151255	1.165596	4.179928
92	1	0	4.134236	1.257105	3.704310
93	1	0	3.144305	0.241394	4.770080
94	1	0	3.037057	2.005469	4.875619
95	6	0	1.611701	4.083504	-2.242684
96	1	0	0.670489	3.550807	-2.070586
97	1	0	1.853761	4.006331	-3.309337
98	1	0	1.445743	5.143211	-2.016673

3b^{II} (UB3LYP/6-31G(d))

Center Number	Atomic Number	Atomic Type	Coordinates (Angstroms) X	Y	Z
1	7	0	-2.961026	1.057691	-0.366287
2	7	0	-2.961016	-1.057685	0.366348
3	7	0	3.630928	0.000001	-0.000011
4	6	0	-2.656033	2.454717	-0.520402
5	6	0	-4.287753	0.636859	-0.214684
6	6	0	-2.094380	0.000004	0.000019
7	6	0	-2.656020	-2.454715	0.520424

8	6	0	-4.287747	-0.636852	0.214779
9	6	0	-0.677671	0.000005	0.000011
10	6	0	4.328981	-1.176188	-0.368953
11	6	0	2.204034	0.000003	-0.000003
12	6	0	1.473835	-1.002958	0.668093
13	1	0	2.011077	-1.782784	1.201243
14	6	0	1.473829	1.002964	-0.668092
15	1	0	2.011067	1.782791	-1.201246
16	6	0	-2.713408	3.013730	-1.816697
17	6	0	-2.375967	3.240946	0.617892
18	6	0	-2.375986	-3.240920	-0.617894
19	6	0	0.091868	-1.014064	0.667631
20	1	0	-0.410026	-1.803833	1.209420
21	6	0	0.091862	1.014073	-0.667615
22	1	0	-0.410036	1.803844	-1.209396
23	6	0	-2.713360	-3.013755	1.816709
24	6	0	4.328988	1.176187	0.368926
25	6	0	3.840884	-2.001023	-1.398313
26	1	0	2.932794	-1.716913	-1.919322
27	6	0	-3.013704	-2.172240	3.054303
28	1	0	-3.098399	-1.129559	2.733903
29	6	0	-2.476048	4.387002	-1.950647
30	1	0	-2.516066	4.841380	-2.936877
31	6	0	-2.132505	4.606157	0.424779
32	1	0	-1.910342	5.233099	1.284003
33	6	0	-3.013788	2.172191	-3.054265
34	1	0	-3.098491	1.129519	-2.733839
35	6	0	3.840902	2.001024	1.398290
36	1	0	2.932816	1.716917	1.919306
37	6	0	-2.182872	5.177683	-0.843337
38	1	0	-1.995374	6.240882	-0.968381
39	6	0	-2.475997	-4.387029	1.950624
40	1	0	-2.515987	-4.841427	2.936846
41	6	0	-2.352767	-2.665166	-2.030585
42	1	0	-2.530362	-1.588645	-1.961710
43	6	0	-2.132518	-4.606135	-0.424815
44	1	0	-1.910379	-5.233059	-1.284058
45	6	0	-2.352704	2.665218	2.030593
46	1	0	-2.530292	1.588694	1.961742
47	6	0	-1.871972	2.241007	-4.086752
48	1	0	-1.733427	3.257490	-4.473177
49	1	0	-2.093971	1.590029	-4.940687
50	1	0	-0.922287	1.913614	-3.650715

51	6	0	5.507303	1.560496	-0.297052
52	1	0	5.890753	0.943935	-1.103186
53	6	0	-4.358700	2.570846	-3.693107
54	1	0	-5.185245	2.476660	-2.979687
55	1	0	-4.579936	1.928759	-4.553845
56	1	0	-4.342586	3.608746	-4.046406
57	6	0	5.507300	-1.560501	0.297017
58	1	0	5.890758	-0.943942	1.103147
59	6	0	-2.182850	-5.177687	0.843290
60	1	0	-1.995347	-6.240889	0.968307
61	6	0	4.505354	-3.173420	-1.750777
62	1	0	4.133287	-3.813108	-2.544754
63	6	0	5.674450	-3.516863	-1.082548
64	6	0	6.186949	2.721452	0.064614
65	1	0	7.096301	3.022335	-0.445851
66	6	0	5.674472	3.516857	1.082512
67	6	0	4.505380	3.173419	1.750749
68	1	0	4.133322	3.813107	2.544730
69	6	0	6.186938	-2.721460	-0.064654
70	1	0	7.096293	-3.022346	0.445804
71	6	0	-1.871873	-2.241096	4.086768
72	1	0	-1.733338	-3.257589	4.473172
73	1	0	-2.093846	-1.590131	4.940720
74	1	0	-0.922189	-1.913711	3.650722
75	6	0	-4.358611	-2.570894	3.693159
76	1	0	-5.185168	-2.476677	2.979757
77	1	0	-4.579823	-1.928828	4.553919
78	1	0	-4.342504	-3.608804	4.046427
79	6	0	-3.483029	-3.262100	-2.892306
80	1	0	-3.363580	-4.344532	-3.019398
81	1	0	-3.482130	-2.807513	-3.890125
82	1	0	-4.465052	-3.085099	-2.439518
83	6	0	-0.979491	2.856280	2.702589
84	1	0	-0.182376	2.412299	2.098313
85	1	0	-0.971102	2.376889	3.688583
86	1	0	-0.746031	3.917709	2.848820
87	6	0	-0.979572	-2.856204	-2.702623
88	1	0	-0.182444	-2.412226	-2.098362
89	1	0	-0.971215	-2.376798	-3.688609
90	1	0	-0.746106	-3.917628	-2.848877
91	6	0	-3.482947	3.262159	2.892334
92	1	0	-3.363503	4.344595	3.019401
93	1	0	-3.482016	2.807592	3.890163

94	1	0	-4.464981	3.085140	2.439578
95	9	0	6.326416	-4.651827	-1.428427
96	9	0	6.326445	4.651818	1.428386
97	1	0	-5.104397	-1.311181	0.418288
98	1	0	-5.104407	1.311189	-0.418170

3b^{III} (UB3LYP/6-31G(d))

Center	Atomic	Atomic	Coordinates (Angstroms)		
Number	Number	Type	X	Y	Z
1	7	0	-3.736171	0.982095	-0.533013
2	7	0	-3.736159	-0.982151	0.532967
3	7	0	2.853103	0.000014	-0.000000
4	6	0	-3.422944	2.327448	-0.935425
5	6	0	-5.062697	0.593039	-0.316876
6	6	0	-2.872387	-0.000022	-0.000022
7	6	0	-3.422910	-2.327490	0.935408
8	6	0	-5.062689	-0.593124	0.316809
9	6	0	-1.455361	-0.000016	-0.000020
10	6	0	3.539767	-1.212531	-0.226366
11	6	0	1.421740	0.000001	-0.000009
12	6	0	0.694536	-0.851705	0.854420
13	1	0	1.232271	-1.509274	1.532415
14	6	0	0.694532	0.851697	-0.854445
15	1	0	1.232265	1.509270	-1.532438
16	6	0	-3.498543	2.650832	-2.308512
17	6	0	-3.111988	3.295972	0.043298
18	6	0	-3.111905	-3.296018	-0.043295
19	6	0	-0.686951	-0.861685	0.856814
20	1	0	-1.188651	-1.525747	1.547354
21	6	0	-0.686955	0.861661	-0.856851
22	1	0	-1.188657	1.525712	-1.547399
23	6	0	-3.498551	-2.650861	2.308496
24	6	0	3.539743	1.212570	0.226376
25	6	0	3.001005	-2.180050	-1.095587
26	1	0	2.069888	-1.970199	-1.610295
27	6	0	-3.835019	-1.612277	3.375633
28	1	0	-3.938869	-0.643783	2.877470
29	6	0	-3.244146	3.975123	-2.684820
30	1	0	-3.294631	4.250233	-3.734700
31	6	0	-2.853285	4.601775	-0.390696
32	1	0	-2.607100	5.366026	0.341222
33	6	0	-3.834952	1.612251	-3.375671

34	1	0	-3.938831	0.643756	-2.877518
35	6	0	3.000947	2.180082	1.095583
36	1	0	2.069822	1.970218	1.610273
37	6	0	-2.918758	4.942479	-1.738517
38	1	0	-2.718063	5.963659	-2.051459
39	6	0	-3.244148	-3.975145	2.684825
40	1	0	-3.294667	-4.250246	3.734706
41	6	0	-3.076034	-2.978472	-1.535006
42	1	0	-3.259811	-1.908049	-1.659399
43	6	0	-2.853199	-4.601813	0.390720
44	1	0	-2.606976	-5.366068	-0.341181
45	6	0	-3.076168	2.978418	1.535008
46	1	0	-3.259967	1.907998	1.659392
47	6	0	-2.706661	1.470435	-4.415988
48	1	0	-2.552237	2.399233	-4.977123
49	1	0	-2.955920	0.684716	-5.138840
50	1	0	-1.757517	1.202417	-3.939796
51	6	0	4.751213	1.501658	-0.431093
52	1	0	5.175693	0.777184	-1.117617
53	6	0	-5.179043	1.925514	-4.062159
54	1	0	-5.997378	1.984027	-3.335484
55	1	0	-5.428156	1.144593	-4.790200
56	1	0	-5.142420	2.881451	-4.597871
57	6	0	4.751229	-1.501600	0.431127
58	1	0	5.175684	-0.777119	1.117660
59	6	0	-2.918714	-4.942505	1.738542
60	1	0	-2.718020	-5.963680	2.051499
61	6	0	3.653212	-3.389145	-1.300832
62	1	0	3.230130	-4.118108	-1.984735
63	6	0	4.861463	-3.664283	-0.652042
64	6	0	5.403070	2.709414	-0.214088
65	1	0	6.336189	2.915239	-0.728780
66	6	0	4.861389	3.664346	0.652073
67	6	0	3.653128	3.389189	1.300838
68	1	0	3.230020	4.118146	1.984730
69	6	0	5.403111	-2.709345	0.214132
70	1	0	6.336223	-2.915156	0.728843
71	6	0	-2.706787	-1.470457	4.416012
72	1	0	-2.552397	-2.399249	4.977168
73	1	0	-2.956084	-0.684730	5.138842
74	1	0	-1.757614	-1.202447	3.939872
75	6	0	-5.179150	-1.925536	4.062045
76	1	0	-5.997444	-1.984038	3.335325
77	1	0	-5.428299	-1.144619	4.790078

78	1	0	-5.142560	-2.881478	4.597750
79	6	0	-4.194396	-3.725762	-2.288443
80	1	0	-4.068351	-4.812726	-2.221011
81	1	0	-4.184807	-3.454657	-3.350807
82	1	0	-5.182100	-3.478093	-1.883727
83	6	0	-1.695329	3.275462	2.150582
84	1	0	-0.906338	2.730695	1.622916
85	1	0	-1.677891	2.972548	3.204090
86	1	0	-1.456601	4.344825	2.110100
87	6	0	-1.695178	-3.275534	-2.150534
88	1	0	-0.906195	-2.730780	-1.622842
89	1	0	-1.677703	-2.972615	-3.204041
90	1	0	-1.456465	-4.344901	-2.110053
91	6	0	-4.194544	3.725720	2.288414
92	1	0	-4.068480	4.812683	2.220997
93	1	0	-4.184993	3.454607	3.350776
94	1	0	-5.182239	3.478071	1.883665
95	6	0	5.529885	-4.993944	-0.828881
96	6	0	5.529784	4.994019	0.828922
97	9	0	5.306645	-5.515048	-2.058609
98	9	0	6.870537	-4.915094	-0.660527
99	9	0	5.083862	-5.914244	0.063833
100	9	0	5.083761	5.914309	-0.063801
101	9	0	5.306512	5.515120	2.058645
102	9	0	6.870440	4.915192	0.660592
103	1	0	-5.878586	1.225273	-0.629135
104	1	0	-5.878569	-1.225374	0.629056

3b^{IV} (UB3LYP/6-31G(d))

Center	Atomic	Atomic	Coordinates (Angstroms)		
Number	Number	Type	X	Y	Z
1	7	0	-3.113413	1.013721	-0.467472
2	7	0	-3.113765	-1.013123	0.467390
3	7	0	3.473883	-0.000304	0.000090
4	6	0	-2.800693	2.378402	-0.802144
5	6	0	-4.439840	0.612823	-0.278156
6	6	0	-2.250832	0.000176	0.000004
7	6	0	-2.801541	-2.377926	0.802034
8	6	0	-4.440051	-0.611798	0.277987
9	6	0	-0.833566	0.000015	0.000030
10	6	0	4.157393	-1.180546	-0.355602
11	6	0	2.041339	-0.000200	0.000070

12	6	0	1.315372	-0.909123	0.795059
13	1	0	1.853518	-1.610528	1.427404
14	6	0	1.315530	0.908840	-0.794928
15	1	0	1.853797	1.610176	-1.427247
16	6	0	-2.881705	2.771398	-2.156462
17	6	0	-2.485785	3.294831	0.224227
18	6	0	-2.486840	-3.294411	-0.224349
19	6	0	-0.065594	-0.917231	0.798340
20	1	0	-0.567827	-1.623437	1.444917
21	6	0	-0.065434	0.917160	-0.798237
22	1	0	-0.567555	1.623469	-1.444796
23	6	0	-2.882878	-2.770971	2.156319
24	6	0	4.157551	1.179842	0.355799
25	6	0	3.604316	-2.056925	-1.310355
26	1	0	2.664937	-1.797664	-1.785637
27	6	0	-3.222421	-1.788854	3.274515
28	1	0	-3.328344	-0.796370	2.826631
29	6	0	-2.629150	4.113508	-2.464889
30	1	0	-2.683768	4.442330	-3.498938
31	6	0	-2.230092	4.621595	-0.142920
32	1	0	-1.980262	5.346825	0.626420
33	6	0	-3.221431	1.789333	-3.274649
34	1	0	-3.327776	0.796915	-2.826718
35	6	0	3.604568	2.056304	1.310530
36	1	0	2.665138	1.797177	1.785785
37	6	0	-2.300994	5.031109	-1.471177
38	1	0	-2.102151	6.067185	-1.731804
39	6	0	-2.630828	-4.113187	2.464703
40	1	0	-2.685697	-4.442048	3.498727
41	6	0	-2.441584	-2.899200	-1.697109
42	1	0	-2.609016	-1.820979	-1.765547
43	6	0	-2.231653	-4.621283	0.142757
44	1	0	-1.981978	-5.346557	-0.626593
45	6	0	-2.440856	2.899688	1.697015
46	1	0	-2.608710	1.821534	1.765495
47	6	0	-2.093232	1.698085	-4.320803
48	1	0	-1.935643	2.654377	-4.832677
49	1	0	-2.344331	0.951836	-5.083681
50	1	0	-1.145006	1.402898	-3.858915
51	6	0	5.376942	1.534395	-0.256954
52	1	0	5.806427	0.885828	-1.012607
53	6	0	-4.564993	2.140061	-3.943840
54	1	0	-5.382724	2.164644	-3.214586

55	1	0	-4.816593	1.396776	-4.709355
56	1	0	-4.526273	3.121162	-4.431613
57	6	0	5.376718	-1.535272	0.257182
58	1	0	5.806274	-0.886769	1.012851
59	6	0	-2.302861	-5.030845	1.470982
60	1	0	-2.104411	-6.067006	1.731577
61	6	0	4.250365	-3.236483	-1.647657
62	1	0	3.817168	-3.896708	-2.392459
63	6	0	5.472579	-3.581741	-1.042503
64	6	0	6.026050	2.711266	0.085272
65	1	0	6.960572	2.974307	-0.400329
66	6	0	5.473050	3.580859	1.042721
67	6	0	4.250771	3.235775	1.647842
68	1	0	3.817643	3.896065	2.392627
69	6	0	6.025673	-2.712231	-0.085033
70	1	0	6.960145	-2.975404	0.400592
71	6	0	-2.094351	-1.698074	4.320850
72	1	0	-1.937188	-2.654451	4.832695
73	1	0	-2.345299	-0.951777	5.083730
74	1	0	-1.145946	-1.403204	3.859127
75	6	0	-4.566209	-2.139146	3.943480
76	1	0	-5.383837	-2.163400	3.214099
77	1	0	-4.817666	-1.395816	4.708999
78	1	0	-4.527907	-3.120288	4.431202
79	6	0	-3.567732	-3.590403	-2.491462
80	1	0	-3.455295	-4.680898	-2.482066
81	1	0	-3.552517	-3.262844	-3.537692
82	1	0	-4.553127	-3.352490	-2.075540
83	6	0	-1.062449	3.182660	2.324683
84	1	0	-0.267369	2.677118	1.767678
85	1	0	-1.038674	2.824920	3.360686
86	1	0	-0.837721	4.255623	2.340519
87	6	0	-1.063211	-3.182666	-2.324628
88	1	0	-0.268004	-2.677462	-1.767497
89	1	0	-1.039175	-2.824875	-3.360607
90	1	0	-0.838892	-4.255714	-2.340500
91	6	0	-3.566834	3.591367	2.491195
92	1	0	-3.453978	4.681819	2.481753
93	1	0	-3.551871	3.263860	3.537444
94	1	0	-4.552270	3.353809	2.075167
95	6	0	6.137682	-4.798743	-1.391140
96	7	0	6.677601	-5.790386	-1.676388
97	6	0	6.138311	4.797771	1.391368

98	7	0	6.678361	5.789341	1.676624
99	1	0	-5.255688	-1.262283	0.550672
100	1	0	-5.255253	1.263560	-0.550911

3c^I (UB3LYP/6-31G(d))

Center	Atomic	Atomic	Coordinates (Angstroms)		
Number	Number	Type	X	Y	Z
1	7	0	-2.653714	-1.127475	0.157125
2	7	0	-2.653612	1.127657	-0.156834
3	7	0	3.899337	-0.000065	-0.000165
4	6	0	-2.424216	-3.555061	0.161202
5	6	0	-2.306030	-2.333195	0.866127
6	6	0	-2.423877	3.555223	-0.161214
7	6	0	-1.819261	0.000050	0.000096
8	6	0	4.599059	-0.697550	-1.016985
9	6	0	-2.135759	-4.747033	0.835535
10	1	0	-2.216876	-5.692800	0.306245
11	6	0	-1.934770	2.312148	-2.231573
12	6	0	-2.847988	3.606430	1.304227
13	1	0	-2.994795	2.573716	1.634295
14	6	0	4.598933	0.697416	1.016793
15	6	0	-2.305974	2.333263	-0.866029
16	6	0	-2.135432	4.747087	-0.835740
17	1	0	-2.216337	5.692923	-0.306540
18	6	0	5.776418	1.409537	0.725814
19	1	0	6.154714	1.421782	-0.291273
20	6	0	-1.740683	4.740308	-2.169744
21	1	0	-1.513184	5.674190	-2.677179
22	6	0	-0.402682	-0.000000	-0.000001
23	6	0	0.363083	1.201541	0.168093
24	1	0	-0.142682	2.147358	0.308984
25	6	0	-2.848614	-3.606067	-1.304168
26	1	0	-2.995536	-2.573312	-1.634049
27	6	0	2.474606	-0.000065	-0.000158
28	6	0	5.777018	-1.408910	-0.726032
29	1	0	6.155566	-1.420613	0.290965
30	6	0	1.745299	-1.195494	-0.157793
31	1	0	2.282691	-2.130414	-0.289189
32	6	0	-1.644714	3.532942	-2.854062
33	1	0	-1.348055	3.536776	-3.899454
34	6	0	4.114124	-0.708461	-2.336446
35	1	0	3.207076	-0.161726	-2.572812

36	6	0	0.363009	-1.201575	-0.168191
37	1	0	-0.142807	-2.147377	-0.309015
38	6	0	-1.644489	-3.533163	2.853887
39	1	0	-1.347620	-3.537140	3.899219
40	6	0	-1.740733	-4.740443	2.169458
41	1	0	-1.513230	-5.674405	2.676744
42	6	0	-4.185281	-4.353056	-1.481682
43	1	0	-4.098832	-5.407322	-1.192582
44	1	0	-4.506229	-4.321924	-2.529860
45	1	0	-4.979045	-3.908354	-0.870269
46	6	0	6.452287	2.096772	1.731543
47	1	0	7.360179	2.641202	1.483743
48	6	0	-1.934539	-2.312267	2.231591
49	6	0	-1.861575	1.032113	-3.062231
50	1	0	-2.094346	0.184359	-2.413317
51	6	0	1.745366	1.195399	0.157550
52	1	0	2.282819	2.130291	0.288890
53	6	0	6.453050	-2.096120	-1.731670
54	1	0	7.361307	-2.639937	-1.483857
55	6	0	-1.861040	-1.032330	3.062377
56	1	0	-2.093825	-0.184483	2.413585
57	6	0	5.965058	-2.107908	-3.040343
58	1	0	6.491366	-2.651428	-3.819921
59	6	0	4.789191	1.411758	3.331061
60	1	0	4.397198	1.404161	4.345149
61	6	0	4.789225	-1.412629	-3.331050
62	1	0	4.396995	-1.405612	-4.345051
63	6	0	4.114274	0.707559	2.336346
64	1	0	3.207578	0.160226	2.572688
65	6	0	-1.754856	4.221418	2.199481
66	1	0	-0.815818	3.662980	2.121806
67	1	0	-2.070118	4.207225	3.249791
68	1	0	-1.548543	5.263819	1.929725
69	6	0	-0.447199	0.790492	-3.625262
70	1	0	-0.158589	1.574729	-4.335381
71	1	0	-0.410440	-0.167429	-4.157537
72	1	0	0.296420	0.765791	-2.823649
73	6	0	5.964558	2.107805	3.040326
74	1	0	6.490729	2.651351	3.819982
75	6	0	-4.184661	4.353386	1.481864
76	1	0	-4.098300	5.407604	1.192556
77	1	0	-4.505413	4.322426	2.530107
78	1	0	-4.978521	3.908550	0.870677

79	6	0	-4.045064	-0.687625	0.326896
80	1	0	-4.308053	-0.623705	1.393728
81	1	0	-4.738136	-1.387423	-0.150914
82	6	0	-1.755595	-4.220849	-2.199700
83	1	0	-0.816597	-3.662327	-2.122133
84	1	0	-2.071051	-4.206574	-3.249950
85	1	0	-1.549131	-5.263259	-1.930093
86	6	0	-0.446536	-0.790906	3.625163
87	1	0	-0.157860	-1.575231	4.335158
88	1	0	-0.409587	0.166965	4.157515
89	1	0	0.296927	-0.766198	2.823404
90	6	0	-2.905711	1.043931	-4.197827
91	1	0	-3.921378	1.188250	-3.811417
92	1	0	-2.882226	0.095036	-4.747037
93	1	0	-2.709084	1.848290	-4.916295
94	6	0	-4.045089	0.688016	-0.326062
95	1	0	-4.308504	0.624167	-1.392787
96	1	0	-4.737884	1.387895	0.152041
97	6	0	-2.904970	-1.044156	4.198162
98	1	0	-3.920716	-1.188398	3.811933
99	1	0	-2.881329	-0.095300	4.747431
100	1	0	-2.708252	-1.848576	4.916540

4a (UB3LYP/6-31G(d))

Center	Atomic	Atomic	Coordinates (Angstroms)		
Number	Number	Type	X	Y	Z
1	7	0	-2.551600	1.106667	-0.149438
2	7	0	4.043713	-0.000090	0.000023
3	6	0	2.620243	-0.000068	0.000008
4	6	0	-2.217667	2.420619	-0.632792
5	6	0	4.862122	0.490110	1.018753
6	6	0	-0.265359	-0.000021	-0.000011
7	6	0	-3.893041	0.668233	-0.102017
8	6	0	-1.686078	0.000019	-0.000025
9	6	0	1.893816	1.203160	0.039508
10	1	0	2.431502	2.147343	0.063711
11	6	0	6.222658	0.314903	0.651690
12	6	0	0.511023	1.211336	0.049683
13	1	0	0.018367	2.170571	0.084593
14	6	0	-2.199389	3.498934	0.282486
15	6	0	-5.037934	1.609504	-0.279359
16	1	0	-5.981688	1.058833	-0.273725

17	1	0	-4.973709	2.155791	-1.227963
18	1	0	-5.083252	2.359498	0.518865
19	6	0	4.501369	1.058100	2.244378
20	1	0	3.458021	1.175820	2.518769
21	6	0	7.231306	0.730995	1.529043
22	1	0	8.277836	0.604019	1.262907
23	6	0	-1.957233	2.610528	-2.008066
24	6	0	-1.887563	4.771960	-0.209643
25	1	0	-1.855062	5.614994	0.474725
26	6	0	6.877956	1.304978	2.747033
27	1	0	7.652441	1.632760	3.435030
28	6	0	5.524985	1.462501	3.097901
29	1	0	5.270340	1.907354	4.056516
30	6	0	-2.463131	3.313358	1.776137
31	1	0	-2.808474	2.285934	1.927274
32	6	0	-1.647311	3.904417	-2.444718
33	1	0	-1.434725	4.074017	-3.496696
34	6	0	-2.004048	1.469181	-3.020465
35	1	0	-2.258532	0.550578	-2.484616
36	6	0	-1.609039	4.976018	-1.558164
37	1	0	-1.363817	5.971705	-1.918273
38	6	0	-3.099366	1.700441	-4.080453
39	1	0	-4.085925	1.826111	-3.619945
40	1	0	-3.151514	0.844994	-4.764192
41	1	0	-2.896277	2.593752	-4.682532
42	6	0	-3.563418	4.258408	2.297368
43	1	0	-3.252592	5.308441	2.248660
44	1	0	-3.788526	4.032729	3.346188
45	1	0	-4.491442	4.162661	1.722946
46	6	0	-0.632291	1.244885	-3.686290
47	1	0	-0.326778	2.116110	-4.277978
48	1	0	-0.676699	0.383791	-4.363719
49	1	0	0.143221	1.054234	-2.938664
50	6	0	-1.173363	3.488985	2.604382
51	1	0	-0.398985	2.779046	2.298321
52	1	0	-1.381621	3.327031	3.668825
53	1	0	-0.766048	4.501225	2.494525
54	7	0	-2.551703	-1.106554	0.149369
55	6	0	-2.217926	-2.420528	0.632768
56	6	0	4.862129	-0.490316	-1.018686
57	6	0	-3.893104	-0.668005	0.101861
58	6	0	1.893777	-1.203271	-0.039511
59	1	0	2.431434	-2.147471	-0.063713

60	6	0	6.222663	-0.315152	-0.651592
61	6	0	0.510984	-1.211404	-0.049709
62	1	0	0.018301	-2.170623	-0.084643
63	6	0	-2.199712	-3.498860	-0.282490
64	6	0	-5.038087	-1.609177	0.279143
65	1	0	-5.981796	-1.058428	0.273420
66	1	0	-4.973982	-2.155441	1.227769
67	1	0	-5.083407	-2.359192	-0.519060
68	6	0	4.501387	-1.058294	-2.244320
69	1	0	3.458042	-1.175981	-2.518734
70	6	0	7.231318	-0.731276	-1.528922
71	1	0	8.277846	-0.604333	-1.262762
72	6	0	-1.957598	-2.610445	2.008062
73	6	0	-1.888045	-4.771910	0.209675
74	1	0	-1.855590	-5.614957	-0.474678
75	6	0	6.877978	-1.305248	-2.746920
76	1	0	7.652468	-1.633053	-3.434900
77	6	0	5.525009	-1.462728	-3.097819
78	1	0	5.270373	-1.907572	-4.056441
79	6	0	-2.463340	-3.313272	-1.776160
80	1	0	-2.808535	-2.285804	-1.927333
81	6	0	-1.647830	-3.904358	2.444751
82	1	0	-1.435319	-4.073964	3.496744
83	6	0	-2.004351	-1.469080	3.020444
84	1	0	-2.258697	-0.550455	2.484569
85	6	0	-1.609618	-4.975977	1.558215
86	1	0	-1.364517	-5.971684	1.918353
87	6	0	-3.099766	-1.700205	4.080362
88	1	0	-4.086309	-1.825764	3.619791
89	1	0	-3.151859	-0.844750	4.764094
90	1	0	-2.896821	-2.593538	4.682459
91	6	0	-3.563726	-4.258178	-2.297445
92	1	0	-3.253043	-5.308253	-2.248714
93	1	0	-3.788749	-4.032475	-3.346278
94	1	0	-4.491768	-4.162304	-1.723073
95	6	0	-0.632614	-1.244929	3.686358
96	1	0	-0.327235	-2.116185	4.278072
97	1	0	-0.676973	-0.383826	4.363779
98	1	0	0.142969	-1.054369	2.938783
99	6	0	-1.173543	-3.489089	-2.604320
100	1	0	-0.399086	-2.779253	-2.298220
101	1	0	-1.381709	-3.327125	-3.668780
102	1	0	-0.766376	-4.501385	-2.494418

4b (UB3LYP/6-31G(d))

Center Number	Atomic Number	Atomic Type	Coordinates (Angstroms) X	Y	Z
1	7	0	-2.737371	1.086896	0.266414
2	7	0	-2.737793	-1.086633	-0.264798
3	7	0	3.840830	-0.000234	-0.000016
4	6	0	-1.872603	-0.000007	0.000627
5	6	0	-2.426338	2.490995	0.297146
6	6	0	-2.427105	-2.490746	-0.296761
7	6	0	-0.455119	-0.000184	0.000512
8	6	0	2.417706	-0.000236	0.000203
9	6	0	0.311728	1.059617	0.595721
10	1	0	-0.191861	1.884214	1.081289
11	6	0	0.311539	-1.060066	-0.594785
12	1	0	-0.192137	-1.884714	-1.080173
13	6	0	-2.121451	3.168549	-0.903251
14	6	0	-4.063974	0.654621	0.155656
15	1	0	-4.880366	1.345252	0.295453
16	6	0	-4.064211	-0.654015	-0.153439
17	1	0	-4.880837	-1.344459	-0.292791
18	6	0	-2.500287	-3.164342	-1.536398
19	6	0	-2.498770	3.165473	1.536333
20	6	0	4.659551	0.970899	-0.579873
21	6	0	1.694285	1.048745	0.596698
22	1	0	2.235251	1.856764	1.081841
23	6	0	-2.121902	-3.169214	0.903030
24	6	0	1.694110	-1.049208	-0.596068
25	1	0	2.234949	-1.857264	-1.081291
26	6	0	-2.835177	-2.443319	-2.839676
27	1	0	-2.931072	-1.377044	-2.614898
28	6	0	4.659753	-0.971379	0.579541
29	6	0	-2.248727	-4.541523	-1.551068
30	1	0	-2.299531	-5.082569	-2.492054
31	6	0	-2.246797	4.542598	1.549951
32	1	0	-2.297025	5.084282	2.490600
33	6	0	-1.864394	4.542970	-0.829403
34	1	0	-1.623974	5.087743	-1.738182
35	6	0	-1.925894	5.226801	0.381277
36	1	0	-1.727055	6.294796	0.413235
37	6	0	-2.091252	2.469564	-2.259156
38	1	0	-2.278234	1.404843	-2.096378
39	6	0	-2.090848	-2.471094	2.259377
40	1	0	-2.277141	-1.406155	2.097269

41	6	0	6.019981	-0.622631	0.368859
42	6	0	6.019852	0.622131	-0.369706
43	6	0	-1.865316	-4.543668	0.828130
44	1	0	-1.624673	-5.089137	1.736432
45	6	0	-1.927531	-5.226632	-0.382992
46	1	0	-1.729045	-6.294667	-0.415788
47	6	0	-1.711463	-2.589806	-3.883972
48	1	0	-0.759207	-2.209568	-3.499427
49	1	0	-1.960490	-2.023722	-4.789329
50	1	0	-1.563065	-3.635282	-4.178276
51	6	0	4.300676	-2.121115	1.289749
52	1	0	3.257953	-2.371660	1.456122
53	6	0	-2.833333	2.445440	2.840223
54	1	0	-2.929153	1.378989	2.616247
55	6	0	4.300220	2.120649	-1.289930
56	1	0	3.257437	2.371210	-1.455906
57	6	0	-4.184522	-2.919088	-3.412865
58	1	0	-4.156111	-3.984194	-3.671578
59	1	0	-4.433013	-2.360897	-4.323205
60	1	0	-4.999340	-2.774742	-2.694249
61	6	0	5.324565	2.928681	-1.777396
62	1	0	5.070490	3.828383	-2.332017
63	6	0	7.029302	1.453077	-0.870287
64	1	0	8.075524	1.200023	-0.716954
65	6	0	6.677210	2.604080	-1.569457
66	1	0	7.452293	3.257066	-1.961127
67	6	0	7.029608	-1.453580	0.869075
68	1	0	8.075775	-1.200536	0.715352
69	6	0	5.325194	-2.929153	1.776841
70	1	0	5.071319	-3.828845	2.331572
71	6	0	-3.211798	2.995600	-3.178229
72	1	0	-4.198027	2.867147	-2.718148
73	1	0	-3.208314	2.453584	-4.131369
74	1	0	-3.082839	4.061400	-3.400795
75	6	0	6.677765	-2.604568	1.568392
76	1	0	7.452985	-3.257561	1.959779
77	6	0	-4.182625	2.921543	3.413269
78	1	0	-4.154232	3.986866	3.671103
79	1	0	-4.430916	2.364061	4.324097
80	1	0	-4.997559	2.776569	2.694913
81	6	0	-1.709495	2.592794	3.884268
82	1	0	-0.757239	2.212409	3.499865
83	1	0	-1.958349	2.027283	4.790029

84	1	0	-1.561174	3.638479	4.177868
85	6	0	-0.712770	2.590605	-2.936107
86	1	0	-0.468916	3.633507	-3.171305
87	1	0	-0.702693	2.028803	-3.877425
88	1	0	0.077484	2.192556	-2.292382
89	6	0	-3.211515	-2.996926	3.178386
90	1	0	-4.197829	-2.866780	2.718947
91	1	0	-3.206947	-2.456053	4.132169
92	1	0	-3.083698	-4.063136	3.399620
93	6	0	-0.712256	-2.593446	2.935879
94	1	0	-0.469058	-3.636617	3.170555
95	1	0	-0.701540	-2.032065	3.877437
96	1	0	0.078090	-2.195656	2.292108

4c (UB3LYP/6-31G(d))

Center	Atomic	Atomic	Coordinates (Angstroms)		
Number	Number	Type	X	Y	Z
1	7	0	-2.662597	1.135284	-0.081384
2	7	0	3.855287	0.000104	0.000381
3	7	0	-2.662860	-1.135006	0.079636
4	6	0	-2.095208	2.589798	1.856511
5	6	0	-2.085580	1.435119	2.856130
6	1	0	-2.284501	0.507772	2.314439
7	6	0	-1.841920	0.000058	-0.000567
8	6	0	-2.376792	2.426662	0.478784
9	6	0	5.339438	-0.543447	3.383449
10	1	0	5.085640	-0.703076	4.428225
11	6	0	6.033794	0.122657	-0.712728
12	6	0	-2.376537	-2.426719	-0.479412
13	6	0	6.033630	-0.122528	0.713973
14	6	0	4.314677	-0.392629	2.452128
15	1	0	3.272276	-0.427231	2.751847
16	6	0	-0.425777	0.000003	-0.000284
17	6	0	4.673898	-0.189839	1.116264
18	6	0	4.674155	0.190010	-1.115321
19	6	0	6.691687	-0.491025	3.000959
20	1	0	7.467040	-0.614733	3.751942
21	6	0	0.330947	1.134015	-0.440376
22	1	0	-0.185552	2.020008	-0.786767
23	6	0	0.330851	-1.133945	0.440122
24	1	0	-0.185691	-2.019973	0.786369
25	6	0	-2.439170	-3.545176	0.387837

26	6	0	-1.850366	3.885058	2.330665
27	1	0	-1.623957	4.028566	3.383772
28	6	0	2.432931	0.000082	0.000199
29	6	0	-2.093708	-2.590674	-1.856788
30	6	0	1.713815	1.130746	-0.428963
31	1	0	2.258134	2.012061	-0.756576
32	6	0	-1.848305	-3.886205	-2.329916
33	1	0	-1.620920	-4.030354	-3.382723
34	6	0	4.315240	0.392836	-2.451263
35	1	0	3.272908	0.427468	-2.751219
36	6	0	-2.747592	-3.396344	1.875847
37	1	0	-2.735715	-2.326027	2.104837
38	6	0	-1.912355	4.992906	1.491870
39	1	0	-1.728717	5.988208	1.887852
40	6	0	7.043797	0.278501	-1.669576
41	1	0	8.089800	0.230385	-1.377092
42	6	0	-2.083249	-1.436586	-2.857057
43	1	0	-2.282390	-0.508903	-2.316028
44	6	0	-2.214287	4.819820	0.144904
45	1	0	-2.265229	5.687750	-0.506917
46	6	0	-2.213994	-4.819635	-0.143871
47	1	0	-2.265361	-5.687162	0.508448
48	6	0	5.340210	0.543638	-3.382357
49	1	0	5.086650	0.703286	-4.427189
50	6	0	7.043415	-0.278377	1.671050
51	1	0	8.089484	-0.230291	1.378795
52	6	0	-1.910886	-4.993523	-1.490473
53	1	0	-1.726766	-5.989049	-1.885668
54	6	0	-2.438806	3.545662	-0.387809
55	6	0	-0.712022	1.272669	3.535107
56	1	0	0.078067	1.119415	2.794121
57	1	0	-0.721862	0.407941	4.209432
58	1	0	-0.452102	2.154661	4.132185
59	6	0	1.713730	-1.130600	0.429156
60	1	0	2.257985	-2.011884	0.756966
61	6	0	-2.745869	3.397806	-1.876209
62	1	0	-2.733857	2.327644	-2.105885
63	6	0	6.692373	0.491179	-2.999560
64	1	0	7.467901	0.614886	-3.750364
65	6	0	-1.682380	-4.061343	2.768611
66	1	0	-1.653901	-5.148396	2.631819
67	1	0	-1.902720	-3.869442	3.825454
68	1	0	-0.681091	-3.670939	2.556966

69	6	0	-4.065293	-0.722092	0.256109
70	1	0	-4.362905	-0.770893	1.313239
71	1	0	-4.727008	-1.375913	-0.317056
72	6	0	-4.064956	0.722528	-0.259097
73	1	0	-4.361564	0.771348	-1.316500
74	1	0	-4.727105	1.376452	0.313439
75	6	0	-3.204498	1.598288	3.905257
76	1	0	-3.056085	2.495238	4.517421
77	1	0	-3.222336	0.734617	4.580839
78	1	0	-4.189770	1.680918	3.431720
79	6	0	-1.679714	4.063315	-2.767482
80	1	0	-1.651110	5.150241	-2.629720
81	1	0	-1.899161	3.872346	-3.824679
82	1	0	-0.678708	3.672500	-2.555267
83	6	0	-4.151382	-3.937780	2.214314
84	1	0	-4.929244	-3.440488	1.624044
85	1	0	-4.379936	-3.784602	3.275931
86	1	0	-4.219245	-5.012992	2.009604
87	6	0	-4.149274	3.939585	-2.215674
88	1	0	-4.927727	3.442027	-1.626405
89	1	0	-4.376850	3.787017	-3.277587
90	1	0	-4.217252	5.014686	-2.010428
91	6	0	-3.201520	-1.600185	-3.906821
92	1	0	-3.052981	-2.497637	-4.518222
93	1	0	-3.218622	-0.736992	-4.583028
94	1	0	-4.187122	-1.682148	-3.433871
95	6	0	-0.709230	-1.274700	-3.535224
96	1	0	0.080463	-1.121645	-2.793770
97	1	0	-0.718368	-0.410040	-4.209645
98	1	0	-0.449239	-2.156841	-4.132054

6. Abstracted results of TD-DFT calculations

3a^{I} (wB97X/6-31G(d,p))

179A is SOMO

State	Energies (eV)	λ (nm)	*f*	D<S^2>	Dominant Excitations	
1	2.2114	560.66	0.0087	0.0447	A(179)->A(180)	30.6%
					A(179)->A(182)	10.9%
					A(179)->A(188)	53.3%
2	2.6671	464.86	0.0062	0.0202	A(179)->A(180)	14.2%
					A(179)->A(181)	66.0%
					A(179)->A(182)	13.6%
3	2.7969	443.30	0.0870	0.0254	A(179)->A(184)	57.2%
					A(179)->A(187)	9.6%
					A(179)->A(191)	19.9%
4	2.9998	413.31	0.0375	0.0287	A(179)->A(183)	24.6%
					A(179)->A(184)	33.5%
					A(179)->A(187)	12.0%
					A(179)->A(191)	17.4%
5	3.1659	391.62	0.0002	0.0366	A(179)->A(180)	10.9%
					A(179)->A(182)	20.1%
					A(179)->A(185)	44.6%
					A(179)->A(188)	14.6%
6	3.1870	389.03	0.0047	0.0279	A(179)->A(183)	71.2%
7	3.3851	366.26	0.0203	0.9270	A(178)->A(182)	9.3%
					A(179)->A(180)	12.4%
					A(179)->A(181)	14.5%
					A(179)->A(182)	21.7%
8	3.5680	347.49	0.0250	0.2004	A(179)->A(189)	77.2%
9	3.7838	327.67	0.8589	0.0596	A(179)->A(187)	9.8%
					A(177)->A(179)	18.2%
					A(178)->A(179)	51.8%
10	3.8245	324.18	0.0018	0.6603	A(178)->A(185)	13.0%
					A(179)->A(182)	8.4%
					A(179)->A(185)	30.1%
					A(179)->A(188)	15.1%

3a^{II} (wB97X/6-31G(d,p))

187A is SOMO

State	Energies (eV)	λ (nm)	*f*	D<S^2>	Dominant Excitations	
1	2.1788	569.04	0.0083	0.0458	A(187)->A(188)	26.0%
					A(187)->A(191)	9.5%
					A(187)->A(196)	55.5%
2	2.6730	463.84	0.0068	0.0207	A(187)->A(189)	86.4%
3	2.7800	445.98	0.0978	0.0268	A(187)->A(193)	52.8%

					A(187)->A(195)	11.7%
					A(187)->A(199)	21.7%
4	3.0043	412.69	0.0321	0.0296	A(187)->A(192)	22.4%
					A(187)->A(193)	37.0%
					A(187)->A(195)	12.7%
					A(187)->A(199)	15.7%
5	3.1543	393.07	0.0004	0.0450	A(187)->A(191)	65.4%
					A(187)->A(196)	13.0%
6	3.1823	389.60	0.0032	0.0271	A(187)->A(192)	73.7%
7	3.3934	365.36	0.0208	0.8971	A(186)->A(190)	19.8%
					A(187)->A(190)	44.5%
8	3.5003	354.21	0.0011	0.6011	A(186)->A(188)	17.9%
					A(187)->A(188)	43.6%
					A(187)->A(196)	18.0%
9	3.5740	346.90	0.0244	0.2047	A(187)->A(197)	78.1%
10	3.7659	329.23	0.7975	0.0883	A(187)->A(195)	9.6%
					A(185)->A(187)	16.3%
					A(186)->A(187)	51.3%

3a^{III} (wB97X/6-31G(d,p))

211A is SOMO

State	Energies (eV)	λ (nm)	*f*	D<S^2>	Dominant Excitations	
1	2.1706	571.19	0.0080	0.0425	A(211)->A(213)	23.7%
					A(211)->A(215)	10.2%
					A(211)->A(220)	55.6%
2	2.7359	453.18	0.0088	0.0230	A(211)->A(214)	84.5%
3	2.7718	447.30	0.1550	0.0274	A(211)->A(216)	42.9%
					A(211)->A(222)	35.4%
4	2.9154	425.27	0.0296	0.3631	A(210)->A(212)	12.1%
					A(211)->A(212)	73.8%
5	3.0466	406.97	0.0274	0.0290	A(211)->A(217)	46.9%
					A(211)->A(218)	12.2%
					A(211)->A(219)	12.6%
					A(211)->A(222)	13.5%
6	3.2149	385.66	0.0003	0.0380	A(211)->A(215)	71.6%
					A(211)->A(220)	14.0%
7	3.2326	383.54	0.0026	0.0243	A(211)->A(216)	32.9%
					A(211)->A(217)	42.6%
					A(211)->A(218)	10.1%
8	3.6306	341.50	0.0235	0.2395	A(211)->A(213)	10.9%
					A(211)->A(220)	9.0%
					A(211)->A(221)	60.6%
9	3.6384	340.77	0.0047	0.7183	A(211)->A(213)	31.5%

					A(211)->A(221)	15.4%
					A(210)->A(212)	9.8%
10	3.6632	338.46	0.0216	1.1825	A(211)->A(212)	11.1%
					A(211)->A(213)	15.0%
					A(210)->A(212)	21.5%

3a^{IV} (wB97X/6-31G(d,p))

191A is SOMO

State	Energies (eV)	λ (nm)	f	D<S^2>	Dominant Excitations	
1	2.1213	584.48	0.0124	0.0471	A(191)->A(192)	9.2%
					A(191)->A(194)	19.9%
					A(191)->A(196)	10.2%
					A(191)->A(200)	51.4%
2	2.4130	513.81	0.0350	0.1971	A(191)->A(192)	76.2%
3	2.6878	461.29	0.2306	0.0396	A(191)->A(193)	29.2%
					A(191)->A(199)	12.9%
					A(191)->A(204)	38.9%
4	2.7615	448.98	0.0068	0.0233	A(191)->A(195)	81.8%
					A(191)->A(196)	11.1%
5	3.0434	407.38	0.0215	0.0428	A(191)->A(199)	74.4%
6	3.2342	383.35	0.0003	0.0374	A(191)->A(196)	70.1%
					A(191)->A(200)	13.2%
7	3.2383	382.87	0.0023	0.0345	A(191)->A(198)	91.9%
8	3.3111	374.45	0.0189	1.7928	A(189)->A(192)	11.0%
					A(190)->A(192)	13.6%
					A(189)->A(192)	13.8%
					A(190)->A(192)	31.2%
9	3.5089	353.35	0.0993	0.3893	A(191)->A(193)	32.1%
					A(191)->A(204)	16.2%
					A(190)->A(191)	15.9%
10	3.5731	346.99	0.0000	0.2102	A(191)->A(194)	62.8%
					A(191)->A(200)	20.4%

3b^{I} (wB97X/6-31G(d,p))

171A is SOMO

State	Energies (eV)	λ (nm)	f	D<S^2>	Dominant Excitations	
1	2.2373	554.18	0.0073	0.0467	A(171)->A(172)	39.0%
					A(171)->A(180)	49.5%
2	2.7269	454.67	0.1212	0.0279	A(171)->A(175)	35.5%
					A(171)->A(177)	16.7%
					A(171)->A(179)	11.6%
					A(171)->A(182)	24.4%
3	2.748	451.19	0.0144	0.0262	A(171)->A(174)	84.7%

4	2.9689	417.6	0.0013	0.0822	A(171)->A(172)	12.1%
					A(171)->A(173)	11.8%
					A(171)->A(176)	26.8%
					A(171)->A(180)	15.6%
					A(171)->A(181)	26.4%
5	3.0682	404.09	0.026	0.0323	A(171)->A(175)	56.2%
					A(171)->A(179)	14.9%
					A(171)->A(182)	15.9%
6	3.2125	385.94	0.0039	0.0276	A(171)->A(177)	79.2%
7	3.4069	363.92	0.0218	0.9805	A(170)->A(173)	20.8%
					A(171)->A(173)	38.2%
8	3.4895	355.3	0.0109	0.1162	A(171)->A(176)	13.0%
					A(171)->A(181)	58.9%
9	3.8542	321.69	0.0018	0.7074	A(170)->A(176)	16.0%
					A(171)->A(176)	32.0%
					A(171)->A(180)	15.3%
10	3.8725	320.17	0.7656	0.1344	A(169)->A(171)	18.0%
					A(170)->A(171)	51.5%

3b$^{\mathbf{II}}$ (wB97X/6-31G(d,p))

179A is SOMO

State	Energies (eV)	λ (nm)	f	D<S^2>	Dominant Excitations	
1	2.2034	562.69	0.0078	0.0472	A(179)->A(180)	27.6%
					A(179)->A(181)	11.7%
					A(179)->A(188)	52.5%
2	2.7434	451.94	0.1195	0.0274	A(179)->A(184)	36.2%
					A(179)->A(185)	12.5%
					A(179)->A(187)	13.5%
					A(179)->A(190)	25.1%
3	2.7607	449.11	0.0116	0.024	A(179)->A(181)	40.5%
					A(179)->A(182)	19.9%
					A(179)->A(183)	31.1%
4	2.9755	416.68	0.0019	0.0837	A(179)->A(181)	30.6%
					A(179)->A(183)	14.3%
					A(179)->A(188)	15.2%
					A(179)->A(189)	25.6%
5	3.0677	404.16	0.025	0.0317	A(179)->A(184)	57.6%
					A(179)->A(187)	13.9%
					A(179)->A(190)	14.9%
6	3.2258	384.35	0.0026	0.025	A(179)->A(185)	83.0%
7	3.4251	361.98	0.0177	0.9711	A(178)->A(182)	16.5%
					A(178)->A(183)	9.0%
					A(179)->A(182)	31.5%

					A(179)->A(183)	15.7%
8	3.4996	354.29	0.0111	0.2752	A(179)->A(181)	10.4%
					A(179)->A(183)	11.5%
					A(179)->A(189)	46.8%
9	3.5482	349.43	0.0042	0.5213	A(178)->A(180)	14.9%
					A(179)->A(180)	33.1%
					A(179)->A(188)	19.2%
					A(179)->A(189)	13.5%
10	3.8418	322.72	0.0315	0.8005	A(178)->A(186)	22.7%
					A(179)->A(186)	49.2%

3b^{III} (wB97X/6-31G(d,p))

203A is SOMO

State	Energies (eV)	λ (nm)	f	D<S^2>	Dominant Excitations	
1	2.1837	567.77	0.0073	0.0435	A(203)->A(205)	25.1%
					A(203)->A(206)	11.4%
					A(203)->A(212)	53.2%
2	2.7065	458.1	0.1696	0.026	A(203)->A(208)	49.7%
					A(203)->A(214)	37.1%
3	2.8139	440.62	0.0169	0.0306	A(203)->A(206)	37.9%
					A(203)->A(207)	51.4%
4	2.9529	419.87	0.026	0.3959	A(202)->A(204)	14.4%
					A(203)->A(204)	70.5%
5	3.0329	408.8	0.0034	0.0838	A(203)->A(206)	33.3%
					A(203)->A(207)	18.9%
					A(203)->A(212)	16.0%
					A(203)->A(213)	24.5%
6	3.1066	399.1	0.0242	0.0323	A(203)->A(208)	24.6%
					A(203)->A(209)	21.9%
					A(203)->A(210)	13.0%
					A(203)->A(211)	16.6%
					A(203)->A(214)	14.6%
7	3.2714	378.99	0.0021	0.0232	A(203)->A(209)	73.6%
					A(203)->A(210)	11.1%
8	3.556	348.67	0.0112	0.1221	A(203)->A(207)	20.4%
					A(203)->A(213)	59.8%
9	3.661	338.67	0.0237	1.2874	A(203)->A(204)	9.6%
					A(203)->A(205)	16.1%
					A(202)->A(204)	26.6%
10	3.6887	336.12	0.0054	0.6272	A(202)->A(205)	9.8%
					A(203)->A(205)	38.4%
					A(203)->A(212)	15.4%
					A(202)->A(204)	9.0%

3b^{IV} (wB97X/6-31G(d,p))

183A is SOMO

State	Energies (eV)	λ (nm)	*f*	D<S^2>	Dominant Excitations	
1	2.1478	577.27	0.0118	0.0477	A(183)->A(186)	20.7%
					A(183)->A(187)	13.1%
					A(183)->A(192)	50.3%
2	2.4704	501.88	0.036	0.2227	A(182)->A(184)	9.1%
					A(183)->A(184)	75.9%
3	2.6595	466.19	0.2267	0.0353	A(183)->A(185)	26.4%
					A(183)->A(190)	10.5%
					A(183)->A(196)	40.3%
4	2.8555	434.2	0.0104	0.0277	A(183)->A(187)	32.9%
					A(183)->A(188)	58.2%
5	3.0715	403.66	0.0024	0.0817	A(183)->A(187)	36.3%
					A(183)->A(188)	17.3%
					A(183)->A(192)	15.2%
					A(183)->A(195)	24.3%
6	3.1048	399.33	0.0214	0.0523	A(183)->A(189)	18.9%
					A(183)->A(190)	56.7%
7	3.2983	375.91	0.0017	0.0344	A(183)->A(191)	89.3%
8	3.3209	373.35	0.0204	1.7689	A(182)->A(184)	15.7%
					A(181)->A(184)	9.3%
					A(182)->A(184)	35.7%
9	3.5496	349.29	0.035	0.639	A(182)->A(185)	11.3%
					A(183)->A(185)	29.8%
					A(183)->A(196)	18.4%
10	3.5842	345.92	0.0097	0.155	A(183)->A(186)	16.1%
					A(183)->A(187)	11.2%
					A(183)->A(188)	15.3%
					A(183)->A(195)	46.3%

3c^{I} (wB97X/6-31G(d,p))

172A is SOMO

State	Energies (eV)	λ (nm)	*f*	D<S^2>	Dominant Excitations	
1	2.5308	489.90	0.0060	0.0549	A(172)->A(173)	18.3%
					A(172)->A(174)	21.8%
					A(172)->A(181)	50.8%
2	2.9114	425.86	0.1388	0.0499	A(172)->A(176)	20.5%
					A(172)->A(178)	12.0%
					A(172)->A(180)	17.3%
					A(172)->A(183)	31.8%
3	3.2201	385.03	0.0127	0.0393	A(172)->A(175)	79.0%
4	3.4317	361.30	0.0007	0.0684	A(172)->A(176)	56.6%

					A(172)->A(180)	14.7%
					A(172)->A(183)	8.2%
5	3.5342	350.82	0.0145	1.3517	A(171)->A(173)	20.8%
					A(171)->A(174)	12.8%
					A(172)->A(173)	15.1%
					A(172)->A(174)	14.4%
					A(171)->A(173)	9.7%
6	3.6142	343.04	0.0017	0.0428	A(172)->A(176)	17.0%
					A(172)->A(178)	76.2%
7	3.6410	340.53	0.0021	0.0609	A(172)->A(177)	72.5%
					A(172)->A(181)	13.6%
8	4.0184	308.54	0.3551	1.0447	A(171)->A(172)	27.0%
					A(171)->A(180)	17.5%
9	4.0238	308.13	0.0021	1.0709	A(171)->A(173)	9.0%
					A(171)->A(174)	12.4%
					A(172)->A(174)	14.0%
					A(172)->A(181)	12.1%
10	4.0637	305.10	0.0006	1.9717	A(168)->A(178)	6.8%
					A(168)->A(178)	7.2%

4a (wB97X/6-31G(d,p))
178A is SOMO

State	Energies (eV)	λ (nm)	f	D<S^2>	Dominant Excitations	
1	2.2310	555.73	0.0066	0.0423	A(178)->A(180)	11.8%
					A(178)->A(181)	18.7%
					A(178)->A(185)	63.7%
2	2.7683	447.87	0.0076	0.0219	A(178)->A(180)	75.1%
					A(178)->A(181)	17.7%
3	2.8608	433.39	0.1110	0.0200	A(178)->A(183)	51.5%
					A(178)->A(189)	28.8%
4	3.1138	398.18	0.0360	0.0227	A(178)->A(182)	23.1%
					A(178)->A(183)	37.4%
					A(178)->A(189)	21.1%
5	3.2607	380.23	0.0003	0.0384	A(178)->A(180)	8.6%
					A(178)->A(182)	55.4%
					A(178)->A(185)	25.1%
6	3.2882	377.06	0.0076	0.0234	A(178)->A(182)	72.9%
					A(178)->A(189)	9.3%
7	3.3398	371.23	0.0048	0.3692	A(177)->A(179)	12.3%
					A(178)->A(179)	77.4%
8	3.6421	340.42	0.0000	1.9834	A(175)->A(179)	33.5%
					A(175)->A(179)	33.0%
9	3.6499	339.69	0.0245	0.2037	A(178)->A(188)	73.8%

10	3.8255	324.10	0.0174	1.6360	A(176)->A(179)	10.2%
					A(177)->A(179)	14.0%
					A(178)->A(179)	14.6%
					A(176)->A(179)	12.9%
					A(177)->A(179)	31.3%

4b (wB97X/6-31G(d,p))

170A is SOMO

State	Energies (eV)	λ (nm)	*f*	D<S^2>	Dominant Excitations	
1	2.2383	553.92	0.0059	0.0432	A(170)->A(172)	31.1%
					A(170)->A(177)	60.6%
2	2.8015	442.56	0.1343	0.0197	A(170)->A(174)	35.9%
					A(170)->A(175)	10.4%
					A(170)->A(178)	11.6%
					A(170)->A(181)	30.9%
3	2.8490	435.18	0.0127	0.0269	A(170)->A(172)	20.1%
					A(170)->A(173)	70.2%
4	3.0658	404.41	0.0020	0.0843	A(170)->A(172)	33.7%
					A(170)->A(173)	8.4%
					A(170)->A(177)	23.7%
					A(170)->A(179)	26.7%
5	3.1698	391.14	0.0309	0.0249	A(170)->A(174)	59.1%
					A(170)->A(178)	9.5%
					A(170)->A(181)	18.5%
6	3.3231	373.10	0.0038	0.0277	A(170)->A(175)	82.6%
7	3.3640	368.57	0.0068	0.4295	A(169)->A(171)	15.9%
					A(170)->A(171)	72.9%
8	3.5901	345.35	0.0116	0.1064	A(170)->A(172)	11.6%
					A(170)->A(173)	15.3%
					A(170)->A(179)	58.5%
9	3.6426	340.37	0.0000	1.9820	A(168)->A(171)	33.4%
					A(168)->A(171)	32.9%
10	3.8498	322.06	0.0109	1.5803	A(169)->A(171)	15.1%
					A(170)->A(171)	17.3%
					A(169)->A(171)	36.9%

4c (wB97X/6-31G(d,p))

171A is SOMO

State	Energies (eV)	λ (nm)	*f*	D<S^2>	Dominant Excitations	
1	2.5936	478.04	0.0027	0.0501	A(171)->A(173)	34.0%
					A(171)->A(178)	58.8%
2	3.0306	409.11	0.1652	0.0356	A(171)->A(174)	14.9%
					A(171)->A(176)	19.4%

					A(171)->A(179)	14.4%
					A(171)->A(181)	35.4%
3	3.4447	359.93	0.0247	0.0862	A(171)->A(173)	29.4%
					A(171)->A(175)	44.6%
					A(171)->A(178)	16.8%
4	3.6162	342.86	0.0012	1.2060	A(170)->A(172)	34.3%
					A(171)->A(172)	33.3%
					A(170)->A(172)	17.8%
5	3.6292	341.63	0.0051	0.1869	A(171)->A(174)	61.7%
					A(171)->A(179)	8.4%
					A(171)->A(181)	9.4%
6	3.3231	340.42	0.0000	1.9911	A(169)->A(172)	33.9%
					A(169)->A(172)	33.6%
7	3.7724	328.66	0.0078	0.0545	A(171)->A(173)	30.8%
					A(171)->A(175)	48.4%
					A(171)->A(178)	15.3%
8	3.8124	325.21	0.0032	0.0770	A(171)->A(174)	12.8%
					A(171)->A(176)	70.7%
9	4.0229	308.19	0.0203	0.7714	A(171)->A(172)	50.2%
					A(170)->A(172)	30.2%
10	4.0773	304.08	0.0065	1.8716	A(165)->A(173)	6.8%
					A(165)->A(173)	7.2%

7. References

1. Fang, J.-K. et al. Synthesis and spectroscopic study of diphenylamino-substituted phenylene-(poly)ethynylenes: Remarkable effect of acetylenic conjugation modes. *Tetrahedron Lett*. **51**, 917–920 (2010).

2. Jafarpour, L., Stevens, E. D. & Nolan, S. P. A sterically demanding nucleophilic carbene: 1,3-Bis(2,6-diisopropylphenyl)imidazol-2-ylidene). Thermochemistry and catalytic application in olefin metathesis. *J. Organomet. Chem*. **606**, 49–54 (2000).

3. Arduengo, A. J. et al. Imidazolylidenes, imidazolinylidenes and imidazolidines. *Tetrahedron* **55**, 14523–14534 (1999).

4. Stoll, S. & Schweiger, A. EasySpin, a comprehensive software package for spectral simulation and analysis in EPR. *J. Magn. Reson.* **178**, 42–45 (2006).

5. Dolomanov, O. V., Bourhis, L. J., Gildea, R. J., Howard, J. A. K. & Puschmann, H. OLEX2: A complete structure solution, refinement and analysis program. *J. Appl. Cryst.* **42**, 339–341 (2009).

6. Bourhis, L. J., Dolomanov, O. V., Gildea, R. J., Howard, J. A. K. & Puschmann, H. The anatomy of a comprehensive constrained, restrained refinement program for the modern computing environment - Olex2 dissected. *Acta Cryst. A* **71**, 59–75 (2015).

7. Sheldrick, G. M. Crystal structure refinement with SHELXL. *Acta Crystallogr., Sect. C* **71**, 3–8 (2015).

8. Gaussian 09 rev. E01 (Gaussian, Inc., 2009).

9. Becke, A. D. Density-functional thermochemistry. III. The role of exact exchange. *J. Chem. Phys.* **98**, 5648–5652 (1993).

10. Zhang, Y. et al. BDF: A relativistic electronic structure program package. *J. Chem. Phys.* **152**, 064113 (2020).

11. Rizzo, F. et al. From blue to green: Fine-tuning of photoluminescence and electrochemiluminescence in bifunctional organic dyes. *J. Am. Chem. Soc.* **139**, 2060–2069 (2017).

12. Lumpi, D. et al. Substituted triphenylamines as building blocks for star shaped organic electronic materials. *New J. Chem.* **39**, 1840–1851 (2015).
